# Identification of endogenous retroviral reading frames in the human genome

**DOI:** 10.1186/1742-4690-1-32

**Published:** 2004-10-11

**Authors:** Palle Villesen, Lars Aagaard, Carsten Wiuf, Finn Skou Pedersen

**Affiliations:** 1 Bioinformatics Research Center, University of Aarhus, Høegh-Guldbergs Gade 10, Bldg. 090, DK-8000 Aarhus, Denmark; 2 Department of Molecular Biology, University of Aarhus, C. F. Møllers Allé, Bldg. 130, DK-8000 Aarhus, Denmark; 3 Department of Medical Microbiology and Immunology, University of Aarhus, DK-8000 Aarhus, Denmark

## Abstract

**Background:**

Human endogenous retroviruses (HERVs) comprise a large class of repetitive retroelements. Most HERVs are ancient and invaded our genome at least 25 million years ago, except for the evolutionary young HERV-K group. The far majority of the encoded genes are degenerate due to mutational decay and only a few non-HERV-K loci are known to retain intact reading frames. Additional intact HERV genes may exist, since retroviral reading frames have not been systematically annotated on a genome-wide scale.

**Results:**

By clustering of hits from multiple BLAST searches using known retroviral sequences we have mapped 1.1% of the human genome as retrovirus related. The coding potential of all identified HERV regions were analyzed by annotating viral open reading frames (vORFs) and we report 7836 loci as verified by protein homology criteria. Among 59 intact or almost-intact viral polyproteins scattered around the human genome we have found 29 envelope genes including two novel *gammaretroviral *types. One encodes a protein similar to a recently discovered zebrafish retrovirus (ZFERV) while another shows partial, C-terminal, homology to Syncytin (HERV-W/FRD).

**Conclusions:**

This compilation of HERV sequences and their coding potential provide a useful tool for pursuing functional analysis such as RNA expression profiling and effects of viral proteins, which may, in turn, reveal a role for HERVs in human health and disease. All data are publicly available through a database at .

## Background

It has become evident that the human genome harbors a fairly small number of genes, and exons account for little over 1% of our DNA. This stands in stark contrast to various types of repetitive DNA, and it has been estimated that transposable elements alone take up almost half of our genome [1]. Among such multi-copy elements are human endogenous retroviruses (HERVs). These represent stably inherited copies of integrated retroviral genomes (so-called provirus structures) that have entered our ancestors' genome. It has been estimated that HERVs and related sequences such as solitary long terminal repeat structures (solo-LTRs) and retrotransposon-like (*env*-deficient) elements constitute approximately 8% of the human genome [1].

Phylogenetic analysis of the retroviral polymerase gene (*pol*) [2] and envelope genes (*env*) [3] have identified at least 26 distinct HERV groups. However, less well-defined sequence comparisons suggest that there may be well over 100 different HERV groups [4,5]. Within the family of *Retroviridae *most of the seven genera are represented by endogenous members, and HERVs are divided into class I, II and III depending on sequence relatedness to *gammaretroviruses*, *betaretroviruses *or *spumaviruses*, respectively. Many HERVs are named according to tRNA usage (i.e. HERV-K has a primer binding site that matches a lysine tRNA), while others have been more or less provisionally named by their discoverer. It seems increasingly clear that the nomenclature for endogenous retroviruses (ERVs) needs to be revised to accommodate such wide diversity. Furthermore, it is evident that many more ERVs are yet to be discovered as retroviral elements are present in most, if not all, vertebrates and even in some invertebrates [6,7].

With a single exception (HERV-K) all HERV groups are ancient (i.e. entered the genome prior to human speciation) and entered our genome at least 25 million years ago [6,8,9] presumably as an infection of the germ-line. Alternatively, it is possible that ERVs have evolved from pre-existing genomic elements such as LTR-retrotransposons [10]. After colonization most HERV groups have spread within the genome either by re-infection or intracellular transposition [11,12] and have reached copy numbers ranging from a few to several hundreds [13]. The vast majority of these provirus copies are non-functional due to the accumulation of debilitating mutations. Indeed, no replication-competent HERVs have yet been described, although fully intact members of the HERV-K group have been reported [14]. Other mammalian species such as mouse, cat and pig harbor modern replication-competent ERVs that to a large extent may interact with related exogenous viruses [15,16].

The presence of endogenous retroviral sequences in our genome has several possible implications: *i*) replication and (random) insertion of new proviral structures, *ii*) effect on adjacent cellular genes, *iii*) long range genomic effects and *iv*) expression of viral proteins (or RNA). Since the majority of HERVs are highly defective no *de novo *insertions have been observed and presumably HERV mobilization very rarely results in spontaneous genetic disorders or gene knock-outs as seen with other active retrotransposons such as L1 elements [17]. However, existing HERV loci have been shown to alter gene expression by providing alternative transcription initiation, new splice sites or premature polyadenylation sites [18]. Moreover, the presence of enhancers and hormone-responsive elements in the LTR structure of existing HERVs may up- or down-regulate the transcription of flanking cellular genes. It has been speculated that transcription initiation from HERVs/solo-LTRs into neighboring genes in the antisense orientation might interfere with gene expression. Alternatively, gene transcripts encompassing antisense viral sequences could down-regulate HERV expression. The human C4 gene may provide an example of the latter, where antisense HERV-K sequences are generated and display an effect on a heterologous target [19]. Such effects may possibly rely on formation of dsRNA and RNA interference. On a genome scale the presence of closely related sequences may trigger events of ectopic recombination and hence lead to chromosomal rearrangements. Sequence analysis of provirus flanking-DNA suggests that this has occurred during primate evolution [20]. The frequency and significance of such events in human disorders are not clear at present. Finally, HERVs may express viral proteins. The common retroviral genes, *gag*, *(pro)*, *pol *and *env *lead to expression of 3 viral polyproteins (Gag, Gag-Pol and Env) that are processed by a viral or host protease into the active structural and enzymatic subunits. Although most HERV genes are no longer intact, a small fraction has escaped mutational decay. For a subgroup of HERV-K (HDTV) all proteins can apparently be expressed and particle formation has been detected in teratocarcinoma cell lines [13]. Furthermore, HERV-K (HDTV) also directs expression of a small accessory protein Rec (formerly cORF) that up-regulates nucleo-cytoplasmic transport of unspliced viral RNA [21,22]. Loci from other HERV groups have maintained a single intact open reading frame, such as the *env *genes from HERV-H [23], HERV-W [24] and HERV-R (ERV3) [25]. Conservation of an open reading frame during primate evolution clearly suggests some biological function. Animal studies have demonstrated that ERV proteins may in fact serve a useful role for the host either by preventing new retroviral infection or by adopting a physiological role. Syncytin, an Env-derived protein that mediates cell-cell fusion during human placenta formation, provides a striking example of the latter [26,27]. Recently, a second Env protein, dubbed Syncytin 2, proposed to have a similar cell-fusion role [28] was identified. Env proteins may also inhibit cell entry of related exogenous retroviruses that use a common surface receptor, and a Gag-derived protein restricts incoming retroviruses in mice [29].

In the literature, expression of HERVs has frequently been linked with human disease including various cancers and a number of autoimmune disorders [30]. While causal links between disease and HERV activity have yet to be established, it is clear from animal models that expression of endogenous retroviral proteins can affect cell proliferation and invoke or modulate immune responses. A few recent examples include *i*) the possible association of Rec (HERV-K) with germ-cell tumors [31], *ii*) the immunosuppressive abilities of HERV-H Env in a murine cancer model resulting in disturbed tumor clearance [32] and *iii*) the possible superantigenic (SAg) properties of envelopes from HERV-K and HERV-W [33,34] and the increased activity of such proviruses in multiple sclerosis [34], rheumatoid arthritis [35], schizophrenia [36] and type-1 diabetes [33]. SAg expression from the HERV-K18 locus may furthermore be induced by INF-α and thus viral infection such as Epstein-Barr virus [37,38]. One major problem in verifying putative disease association is the multi-copy nature of HERVs and the ambiguous assignment to individual provirus; a problem that can be solved by properly annotating the human genome.

Among Env-associated effects the mechanism of SAg-like activity is believed to involve true epitope-independent stimulation of T-cells, while the mechanism of action of the immunosuppressive CKS-17-like domain is still unknown. This immunosuppressive peptide region maps to the envelope gene [39] and may significantly alter the pathogenic properties of retrovirus and even enhance cancer development. Phylogenetic analysis suggests that a CKS17-like motif arose early in the evolution of retrovirus and is widespread in many current HERV lineages [3], thus identification of novel envelope genes attracts particular attention.

Computer-assisted identification of HERV loci has previously been reported. These include searching conserved amino-acid motifs within the *pol *gene [2,40] and *env *gene [3], detection of full-length *env *genes by nucleotide similarity [41] and compiling of LTR- or ERV-classified repeats as reported by RepeatMasker analysis [4,5,42]. Currently only Paces *et al. *[5,42] provide a searchable database where individual loci are mapped as chromosomal coordinates [43]. However, except for detection of 16 full-length *env *genes in a recent survey by de Parseval *et al *[41] and a detailed analysis of intactness of HERV-H- related proviruses [40], no one has systematically detected HERV regions and scanned them for content of viral open reading frames. In this paper we report mapping of 7836 regions in the human genome that show sequence resemblance to known retroviral genomes which cover the majority of large proviral structures or HERV loci, and, importantly, provide a detailed annotation of all viral open reading frames.

## Results

**Figure 1:**
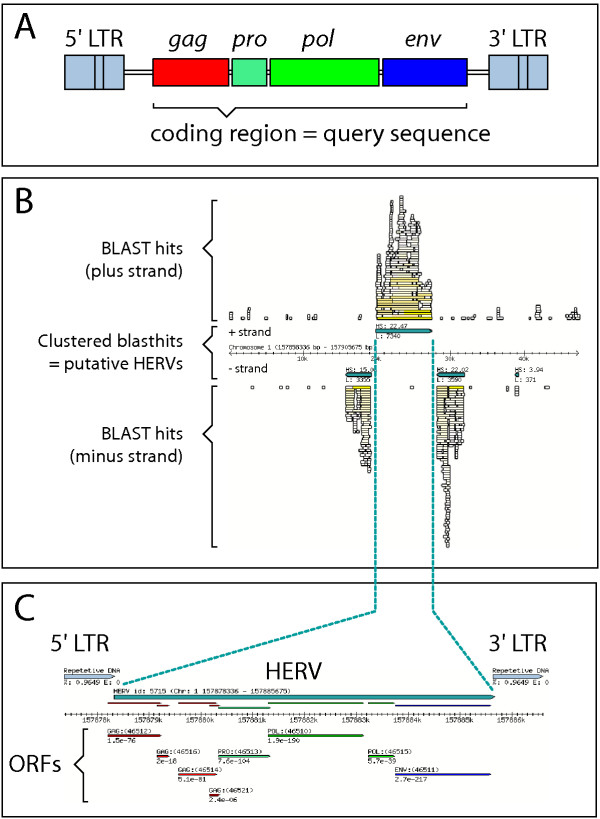
**A: **Genomic organization of simple retroviruses when present as a provirus (DNA) integrated in the host genome. The regulatory long terminal repeats (LTRs) flank the internal three major genes *gag*, *pol *and *env*. A fourth gene *pro *is present between *gag *and *pol *for some retroviruses, while part of either *gag *or *pol *in others. **B: **Individual BLAST hits (white and yellow boxes) on either strand of the human genome were clustered into HERV regions (blue boxes) or discarded by using a score function. Finally, only HERV regions with at least one retroviral ORF were kept (see Materials and Methods). In the example illustrated HERV ID 5715 was presumably inserted into an existing HERV locus with the opposite orientation. HERV ID 5715 is located in the first intron of the CD48 gene (antisense direction) and is also known as HERV-K18 or IDDMK_1,2_22. **C: **HERV ID 5715 with graphical vORF annotation. Putative LTR structures are indicated and all ORFs (stop-codon to stop-codon fragments above 62 aa) are mapped and annotated by homology criteria

In order to screen the human genome for HERV-related sequences we have performed multiple nucleotide BLAST searches and subsequently clustered neighboring hits into larger regions up to about 10 kb in size (Figure 1A/1B). The query sequences cover all known retroviral genera and include both endogenous and exogenous strains from various host organisms. To avoid detection of solo-LTR structures we used the coding regions as query (Figure 1A). The corresponding DNA sequences were scanned for the presence of all viral open reading frames (vORFs, here defined as a stop codon to stop codon fragment above 62 codons) with significant homology to known retroviral proteins (E<0.0005) and annotated as Gag, Pol or Env. From our initial BLAST-identified regions we detect 7836 genuine HERV-related regions in which at least one, mostly several vORF can be detected. The majority of these HERV regions correspond quite well to the internal parts of a provirus locus. However, the insertion of other repetitive elements inside a provirus will produce a mosaic structure that is less well-defined. In terms of our HERV regions this may lead to either "partition" of a provirus into two or more consecutive HERV regions (as illustrated by the "provirus into provirus" insertion depicted in Figure 1B) or enclosure of minor stretches of non-retroviral DNA (such as Alu elements or small microsatellites) within the sequences of some HERV regions. Hence, the precise boundaries of the retrovirus-related DNA (as often defined by nucleotide similarity alone) must be manually inspected and the flanking LTRs must be identified in order to deduce the exact proviral structure. To assist in LTR determination we have scanned for flanking direct repeats and included LTR elements as identified by RepeatMasker analysis [4]. Due to these exceptions we shall refer to our data as "HERV regions" although they in most cases correspond to individual HERV loci.

The average region size is 4300 nucleotides and the ~7800 HERV regions cover ~1.1% of the human genome. All data are publicly available as a searchable database at  Our data include *i*) chromosomal coordinates and sequence information of the 7836 HERV regions, *ii*) annotation of ~38000 retroviral ORFs within these regions and *iii*) graphical visualization of individual HERV regions (Figure 1C) or larger chromosomal window. All DNA and predicted vORF sequences can be retrieved and is linked to external genome browsers for further analysis.

### Skewed chromosomal distribution and few intragenic HERVs

The 7836 HERV regions (~2.7 per Mb) are not uniformly distributed among the 22+2 chromosomes (χ^2 ^test, P~0). Table 1 summarizes the genome distribution statistics, from which it is clear that chromosomes 2, 7, 9, 10, 15, 16, 17, 20 and 22 are less densely populated, while chromosomes 4, 19, X and Y have higher density than expected from a random distribution. In particular, the Y chromosome stands out with more than 14 HERVs per Mb. The distribution of HERVs per Mb along each of the chromosomes is also not uniform, perhaps except at chromosome 21 (Table 1). Furthermore, we observe local "hotspots", most prominent for chromosomes 19, X and Y. For instance, a 5 Mb window in chromosome Y (position 18–23 Mb) encompasses 120 HERV regions. Moreover, there are a number of cases where HERVs have presumably been inserted right next to or even into an existing locus. HERV ID 5715 provides a nice example of the latter, where a HERV-K member presumably has integrated into an existing HERV-K (Figure 1B). We also detect the perfect HERV-K tandem repeat previously reported [44]. However, in contrast to Reus et al. [44] we find a single in-frame stop codon within both *gag *genes (HERV ID 26658–9. W). We also find other examples of closely situated HERV loci as for instance HERV ID 44313 that is composed of two proviruses of distinct origin (HERV-K and a γ-retrovirus-like sequence) both severely degenerated.

**Table 1:** Genomic distribution of HERV regions

Chr.	Length (Mb)	Windows analyzed^a^	Observed HERVs	Expected HERVs	χ^2 ^test^b^	χ^2 ^test within chr.^c^
1	246	228	654	614.7	0.0987	<0.0001
2	243	242	534	652.4	1.3E-06	<0.0001
3	199	197	581	531.1	0.0250	<0.0001
4	192	190	641	512.2	4.0E-09	<0.0001
5	181	180	446	485.3	0.0656	<0.0001
6	171	169	496	455.6	0.0513	<0.0001
7	159	157	342	423.3	4.9E-05	0.0003
8	146	145	396	390.9	0.7923	<0.0001
9	136	120	258	323.5	2.0E-04	<0.0001
10	135	135	304	364.0	1.3E-03	<0.0001
11	134	133	379	358.6	0.2695	<0.0001
12	132	132	393	355.9	0.0440	<0.0001
13	113	98	239	264.2	0.1146	<0.0001
14	105	88	205	237.3	0.0335	<0.0001
15	100	83	135	223.8	1.7E-09	<0.0001
16	90	82	101	221.1	2.6E-16	<0.0001
17	82	80	98	215.7	4.4E-16	<0.0001
18	76	77	167	207.6	0.0043	0.0001
19	64	57	259	153.7	9.4E-18	<0.0001
20	64	62	76	167.2	1.0E-12	<0.0001
21	47	36	85	97.1	0.2181	0.0588
22	49	36	55	97.1	1.7E-05	<0.0001
X	154	152	629	409.8	9.7E-29	<0.0001
Y	50	25	359	67.4	1.1E-278	<0.0001
TOTAL	3068	2905	7832	7832^d^		

^a ^Only windows overlapping with NCBI GoldenPath (release 34)

^b ^Single chromosomes tested against group of other chromosomes. P-values below the significance level 0.00208 (0.05/24, Bonferroni corrected) are underlined.

^c ^The genomic positions of HERVs were χ^2 ^tested against a random distribution using 10000 simulations for each chromosome.

^d ^Four additional HERV regions are located in the DR51 haplotype of the HLA region on chromosome 6 and not counted here.

**Figure 2:**
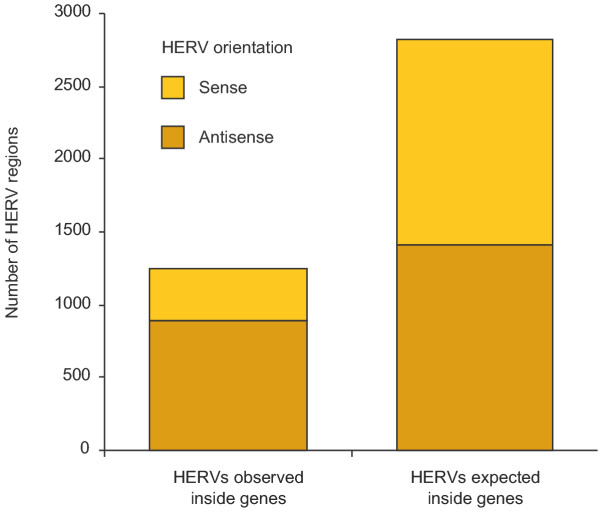
Number of HERV regions located inside genes, and their orientation relative to the gene. The expected number assumes a random genomic distribution.

The number of HERV regions that are located within (non-HERV) genes are significantly reduced as compared to a random distribution (χ^2 ^test, P < 10^-300^), and only 13% of our 7836 HERV regions are situated inside a gene, despite that 33% of the genome is spanned by genes (Figure 2). In total 813 genes (see Additional file 1) carry one or more HERV regions within their predicted boundaries and as such provide a valuable set of genes that may show altered expression due to the presence of internally located proviruses. There is a strong bias (χ^2 ^test, P < 10^-52^) for intragenic HERVs to be orientated antisense relative to the gene (Figure 2). HERV sequences located between genes are equally distributed between the two strands, and the orientation does not depend on the distance from the gene (data not shown).

### Limited number of intact viral open reading frames

**Figure 3:**
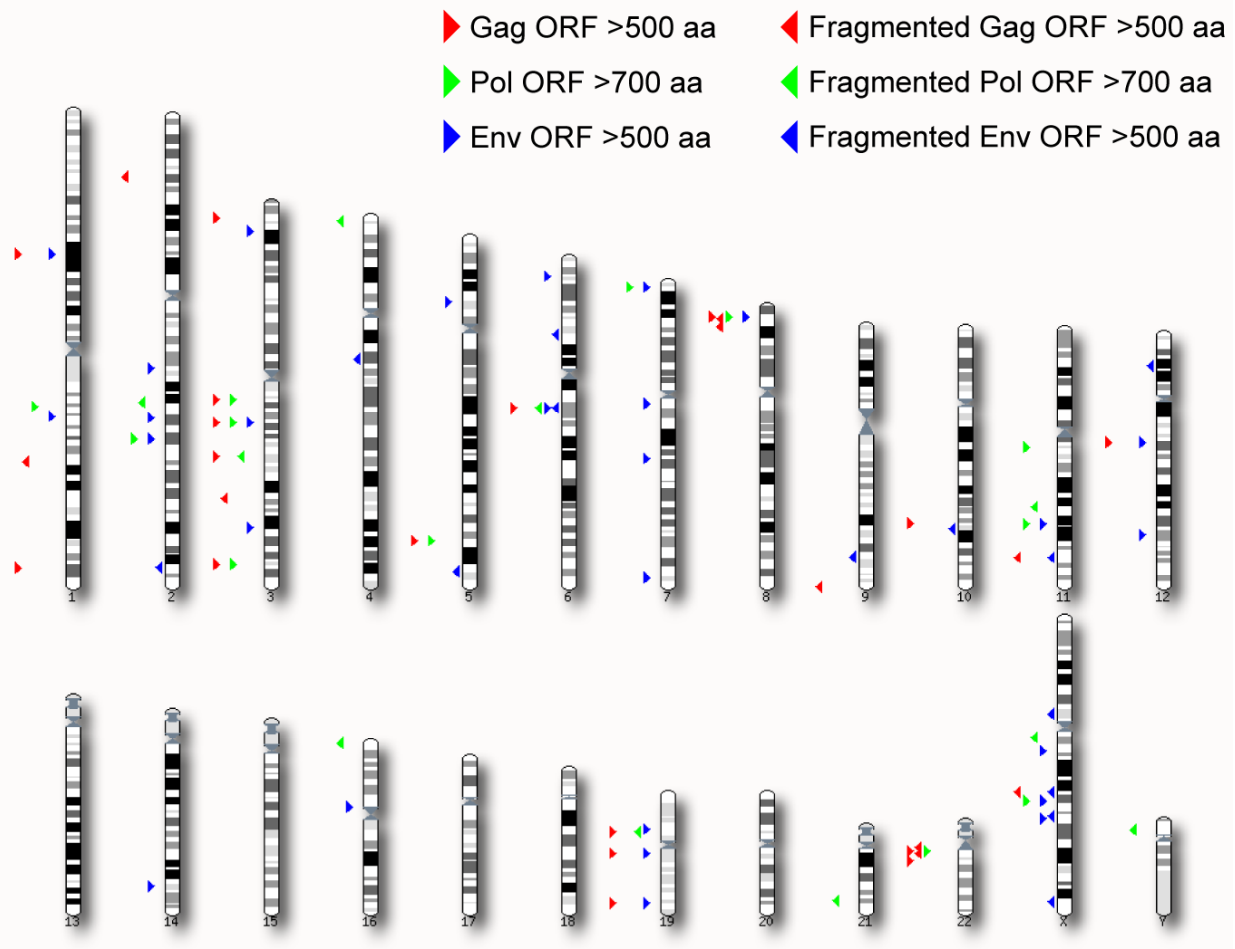
Genomic distribution of all Gag (red) and Env (blue) ORFs above 500 aa and Pol (green) ORFs above 700 aa. Right-pointing triangles denote intact ORFs, while left-pointing triangles denote ORFs that are almost-intact besides a single stop codon or frame-shift mutation.

Of the ~38000 retroviral ORFs 25% are classified as Gag, 7% as Pro, 55% as Pol and 13% as Env proteins. This correlates well with the expected size of the *gag*, *pro*, *pol *and *env *genes, although Pol may be slightly overrepresented. The far majority of the vORFs (stop to stop) are short (Table 2) and presumably do not encode any functional proteins, although a role in cellular processes cannot be excluded. Long vORFs on the other hand may still retain their original viral function. In total 42 HERV regions encompass either a Gag or an Env ORF above 500 codons or a Pol ORF above 700 codons (which approach the size of intact viral proteins) and together they count 17 Gag, 13 Pol and 29 Env proteins (Table 2 and Figure 3). Only two HERV-K related loci (HERV ID 13983 and 29013) carry long reading frames for all viral genes. However, none of them are completely intact. In fact, 41 of the above 59 long vORFs, are all *betaretroviral *and stem from the HERV-K group. Interestingly, 15 of the remaining 18 non-*betaretroviral *ORFs are envelope proteins (see below). Our method only detects a single non-*betaretroviral *Gag ORF above 500 codons (which is located in a *gammaretroviral *structure, HERV ID 44200–1), while two long Pol ORFs are both present in full-length HERV-Fc and HERV-H elements (HERV ID 1178 and 10816) that also harbour intact *env *genes [41,45].

**Table 2:** Distribution of vORF lengths (stop codon to stop codon)

vORF size (aa/codons)	Gag	Pro	Pol	Env	HERV regions
63 – 100	4820	1322	10390	2354	6795
100 – 200	4015	1002	9110	2278	5803
200 – 300	643	165	1426	361	1894
300 – 400	160	54	286	81	527
400 – 500	33	3	70	24	123
500 – 600	1		20	12	33
600 – 700	4		10	9	22
700 – 800	10		4	7	15
800 – 900			1	1	2
900 – 1000	1		5		6
> 1000	1		3		4

If one extends the search criteria and scans the human genome for retroviral genes where a single mutation (one nucleotide insertion, deletion or substitution) either removes premature termination or restores the correct reading frame, the number of long Gag, Pol and Env proteins increases two-fold to 27, 23 and 43, respectively (Figure 3).

### Novel envelope genes identified

Our method detects 29 Env ORFs (stop to stop) above 500 codons (Table 3), which comprise a few seemingly intact or almost-intact *env *genes in the human genome not previously reported. One particularly interesting locus (HERV ID 40701) shows similarity to a recently reported full-length endogenous retrovirus from Zebrafish (*Danio rerio*), dubbed ZFERV [46]. A phylogenetic analysis of the Zebrafish ERV suggested that it is distinct from existing retrovirus genera being most similar to *gammaretroviruses *[46]. An analysis of a short Gag and Pol ORF upstream of the Env gene (HERV ID 40701) confirms the relatedness to *gammaretroviruses *(weak similarity to Feline leukemia virus). Also, two loci (HERV ID 44200–1 and 44204–5) harbor novel Env-like ORFs that C-terminally show homology to Env from HERV-W/syncytin-1 [26,27] and HERV-RFD/syncytin-2 [28], while the N-terminal sequences show no clear homology. The identified ORFs are highly similar (96% aa identities) except for a small C-terminal truncation and both genes are located within a narrow 40 kb region at chromosome 19 (Table 3). Interestingly, both these loci are positive in our EST mapping analysis (see below). Furthermore, among the 29 Env ORFs, five turned out to carry a specific 292 bp deletion (indicative for type 1 HERV-K-HML-2) that fuses the *pol *and *env *reading frames. The same deletion is present in the HERV-K18 Env locus that has been reported to have SAg-like activity [37].

**Table 3:** Previously and newly identified long Env ORFs in the human genome

Gene^a^	Bibliographic name	Chromosomal position of locus (NCBI release 34)	Length^c^	ORF ID	Comment	EST matches^d^
HERV H- like Env		Chr. X 70307525–70316940 (+1)	474	4769	N-term unknown Minor C-term deletion	
EnvF(c)1		Chr. X 95868842–95875915 (+1)	583	8944	Intact^a^	
HERV-W Env		Chr. X 105067535–105070015 (-1)	475	24413	Minor N-term deletion	3
HERV-K Env (type 1)		Chr. 1 75266332–75270814 (+1)	586	42910	In frame pol-env fusion	3
HERV-K Env (type 1)	K18-SAg IDDMK_1,2_22	Chr. 1 157878336–157885675 (+1)	560	46511	In frame pol-env fusion	
EnvH3	EnvH/p59	Chr. 2 155926784–155933168 (+1)	554	70149	Intact^a^	
HERV-K Env (type 1)		Chr. 2 130813720–130815944 (-1)	687	80419	In frame pol-env fusion	
EnvH1	EnvH/p62 H19	Chr. 2 166767087–166774769 (-1)	583	82113	Intact^a^	
EnvR(b)		Chr. 3 16781208–16788508 (+1)	513	86185	Intact^a^	
HERV-K Env (type 1)		Chr. 3 114064939–114072223 (-1)	597	103885	In frame pol-env fusion C-term deletion	
EnvH2	EnvH/p60	Chr. 3 167860265–167867997 (-1)	562	107739	Intact^a^	
HERV-K-like Env		Chr. 5 34507318–34513254 (-1)	475	153615	N- and C-term deletion	
EnvFRD	Syncytin 2	Chr. 6 11211667–11219905 (-1)	537	171089	Intact^a^	16
EnvK4	HERV-K109	Chr. 6 78422690–78431275 (-1)	697	174741	Intact^a^	
EnvK2^b^	HML-2.HOM HERV-K108	Chr. 7 4367317–4383401 (-1)	698	188263 188274	Intact^a^	4
EnvR	Erv3	Chr. 7 63862984–63871411 (-1)	605	191393	Intact^a^	17
EnvW	Syncytin (1)	Chr. 7 91710047–91718755 (-1)	537	192333	Intact^a^	100
EnvF(c)2		Chr. 7 152498159–152502575 (-1)	545	195475	Intact^a^	1
EnvK6	HERV-K115	Chr. 8 7342682–7353583 (-1)	698	204173	Intact^a^	
HERV-K Env		Chr. 11 101104479–101112064 (+1)	661	240932	Minor C-term deletion	6
HERV-K-like Env		Chr. 12 104204746–104209814 (+1)	658	255589	Minor C-term deletion	
EnvK1		Chr. 12 57008431–57016689 (-1)	697	260042	Intact^a^	
ZFERV-like Env		Chr. 14 91072914–91085655 (-1)	664	285129		
HERV-K Env (type 1)		Chr. 16 35312483–35314318 (+1)	550	293143	In frame pol-env fusion	
EnvT		Chr. 19 20334642–20343232 (+1)	664	310016	Intact^a^	
HERV-W/FRD-like Env		Chr. 19 58210000–58211244 (+1)	477	312172	N-term unknown Minor C-term deletion	3
HERV-W/FRD-like Env		Chr. 19 58244133–58246051 (+1)	535	312208	N-term unknown	3
EnvK3	HERV-K (C19)	Chr. 19 32821287–32829201 (-1)	698	314652	Intact^a^	

^a ^Nomenclature for verified and complete *env *genes as in de Parseval et al. [41]. Note that EnvK5 (HERV-113) at Chr. 19 [14] is not present in the NCBI release 34 of the human genome.

^b ^EnvK2 is organized as a tandem repeat.

^c ^ORF length from start to stop codon.

^d ^Number of ESTs that map to the same genomic region (see text).

### EST matching to HERV regions with long ORFs

We mapped 265 ESTs to one of the 42 HERV regions that encode a long Gag, Pol or Env ORF (Figure 3). The EST GenBank accession number, the matching HERV ID and the source organ and tissue type are provided as supplementary material (see Additional file 2). Briefly, 20 of the 42 HERV regions were found to have matching ESTs suggesting transcriptional activity. For the long envelope genes we have included the number of EST matches in Table 3. Our analysis reveals that besides "activity" of members of the HERV-K group, only HERV-Fc(2), HERV-R (Erv3) and a few HERV-W/FRD members (including Syncytin-1 and -2) have unambiguous EST matches. By far, Syncytin-1, dominates with 100 EST matches, followed by Syncytin-2 and HERV-R. Syncytin-1 and Syncytin-2 were predominantly found in placental EST libraries (see Additional file 2), which is also true for 5 of 17 HERV-R ESTs. Interestingly, among the two (partial) HERV-W/FRD-like *env *genes four of 6 ESTs are also derived from placental tissues.

## Discussion

We report a mapping of 7836 loci in the human genome that show nucleotide sequence similarity to retroviral genomes and importantly, we provide a detailed analysis of their coding potential by annotation of all viral ORFs (stop-codon to stop-codon fragments longer than 62). This compilation of HERV regions and their corresponding viral ORFs is available as a searchable database [47]. A graphical example is provided in Figure 1C. In total our HERV regions (which exclude flanking LTRs) amount to 1.1 % of the human genome, a number that agrees well with previous reports [1,42].

The vast majority of the mapped HERV regions contain several frame-shift mutations or in-frame stop codons that truncate the viral ORFs and thus testify to their old association with the human genome. In fact, we detect only 42 proviruses that have retained Gag, Pol or Env ORFs in the size range that approach full-length proteins (Figure 3 and Table 2). As expected the majority are part of the evolutionary young HERV-K (HML-2) group. Neither of these HERV-K loci are completely intact, although one potential replication-competent locus (HERV-K113, polymorphic for humans and not present in the NCBI34 genome) has been reported [14]. Alternatively, complementation among HERV-K loci may open up for infectious particle formation, and clearly defines interesting candidates to investigate experimentally. Moreover, assuming a high error-rate during transcription or retrotransposition, one cannot exclude that almost-intact loci may occasionally revert to their original functional state and become replication-competent. Based on our data about 34 *gag*, *pol *or *env *genes can be restored by a single point mutation or a single insertion-deletion event.

Within our list of intact or almost-intact viral ORFs in the human genome, we detect only a single *gag *gene and two *pol *genes that are not from the HERV-K group. However, among the 29 long envelope genes 15 are *gammaretroviral *(Table 3). The fragmented, pseudogene nature of the *gag *and *pol *genes (small ORFs) in several of these provirus loci strongly suggests that selection has preserved the *env *genes. In case of *syncytin-1 *and *-2 *(HERV-W and HERV-FRD members, respectively) evolutionary conservation can be understood in functional terms, since the encoded envelope proteins have been suggested to play an essential role in placental development by causing trophoblast syncytia formation [28,48]. Compelling evolutionary evidence for purifying selection in these genes has recently been gathered to support this hypothesis [28,49,50].

Concerning other ancient loci such as HERV-R (erv3) no evidence for a physiological role has yet been established despite a remarkable conservation and expression of the *env *gene. Potential cellular roles for envelope genes that may drive purifying selection include *i*) protection from infection by related retroviruses by receptor interference as demonstrated for the murine *fv4 *locus [51], *ii*) mediator of organized cell-cell fusion like the *syncytin *genes [26-28] and *iii*) a hypothesized role in preventing the immune response against the developing embryo by means of the immunosuppressive domain [52].

Two seemingly intact *env *genes not detected in the recent survey of intact human envelope genes [41] are equally interesting in terms of possible functional conservation. One is located on chromosome 14q32.12 and this novel gene shows low but significant similarity to a recently reported endogenous retrovirus from Zebrafish (ZFERV [46]). BLAST analysis of the protein coding regions suggests that this HERV group belong to the *gammaretroviral *genera. Whether this gene is still active or whether the encoded protein still maintains function and/or plays a cellular role is yet to be established. Although we were unable to detect any unambiguous EST matches to this gene (Table 3), RT-PCR analysis indicates low RNA abundance in a few human tissues including placenta (Kjeldbjerg AL, Aagaard L, Villesen P and Pedersen FS, unpublished). A second seemingly intact novel *env *gene is found on chromosome 19q13.41, and interestingly a C-terminal truncated "twin" gene is located just 40 kb away. Both genes appear to be active as judged by EST data (Table 3) mostly in placental tissue (see Additional file 2). We have been able to confirm this by RT-PCR analysis (unpublished), and ongoing expression analysis aims at clarifying the activity and function of these novel genes.

Among the long *betaretroviral env *genes five turned out to carry a specific 292 bp deletion that fuses the pol and env reading frames. This deletion variant of the HERV-K (HML-2) group is indicative of the type 1 genomes [53] that despite the lack of functional proteins have been mobilized quite efficiently. Alternatively, recombination or gene conversion may have conserved this HERV-K deletion variant [11,54]. It is noteworthy that the Env protein from one of these Ä292-genes, HERV-K18, is reported to have SAg-like activity [37], and a similar function of the other four K18 SAg-like genes is an open question.

Although our analysis is extensive it is most likely not exhaustive. The sensitivity is obviously limited by our query sequences, and some ancient HERVs may have suffered from the mutational decay to a degree which makes is impossible to detect them by homology. For instance, the ZFERV-related *env *gene reported by us was only detected due to inclusion of the ZFERV sequence [46], and although available data such as HERVd [43] also points to this region it is reported as a number of incomplete HERVs. Similarly, nucleotide based searches (as RepeatMasker and BLAST detection) only partially detect the novel HERV-W/RFD-like envelope genes and the intact envelope genes among HERV-Fc family even though these proviruses are fairly intact as suggested by a recent mobilization of HERV-Fc in the primate lineage [45]. Thus, inclusion of more retroviral query sequences as our vORF validated HERV data may likely improve detection methods in an iterative manner ("phylogenetic walking") as previously applied by Tristem [2]. Finally, screening the human genome in silico does not guarantee detection of polymorphic HERV loci in which the empty pre-integration site is still segregating in the human population. Indeed, an experimental survey has recently detected two such polymorphic loci in the human population (HERV-K113 and 115 [14]), and like HERV-K113 other recently acquired proviruses may escape our attention.

In general, our analysis of the genomic positions of our ~7800 HERV regions revealed three distinct patterns, which all confirm earlier reported results: *i*) there is an unequal distribution of HERVs between chromosomes and along the genome. In particular the Y chromosome stands out with a five-fold excess of our vORF positive (internal) HERV sequences (Table 1), and it has thus been dubbed "a chromosomal graveyard" [55]. This agrees well with previous genome surveys of LTR/ERV-related elements and the phenomenon may likely be associated with the high level of heterochromatin and low levels of recombination [55-58]. *ii*) HERVs are underrepresented within genes and *iii*) HERVs found in introns are predominantly orientated in the antisense direction (Figure 2). This pattern is well known [56,58] and expected due to selection against gene disruption or interference by retroviral regulatory elements such as promoters, splice sites and polyadenylation signals. This selection may have counteracted a preference for proviral integration (and retrotransposition) near or inside genes as suggested by recent studies for several retroviral genera [59,60].

## Conclusion

Initially, HERV discovery was driven by the search for replication-competent viruses and their possible association with human cancers as established in other species. Recent research has demonstrated that the presence of endogenous retroviral sequences in our genome has a number of complex functional and evolutionary consequences and cannot simply be regarded as "junk" DNA. The increased complexity and diversity of HERVs as testified by the identification of two novel *env *genes in this survey make expression analysis and functional assessment a difficult task. To aid this process our genome-wide HERV data as well as predictions of Gag, Pol and Env reading frames in these loci are a useful resource and our data can be searched and visualized at  Clearly, the 42 HERVs encompassing intact or near-intact *gag*, *pol *and *env *genes as described here are interesting experimental objects, although less intact viral proteins may also hold biological activity. In the near future use of comparative genomics and mapping of allele polymorphisms will most certainly enhance identification of endogenous retroviruses and reveal selection patterns that may eventually decipher a role for these genes in human health and/or disease.

## Methods

In order to identify HERV regions in the human genome we performed BLAST searches using sensitive parameters. BLAST hits were saved in a database and subsequently clustered into putative HERV loci. These putative loci were then scanned for viral Open Reading Frames (vORFs) and the presence of flanking direct repeat sequences (putative LTRs). Subsequently, ORFs were categorized based on a library of known retroviral proteins and non-retroviral proteins.

### Identifying HERV regions

In order to cover as many different HERV families as possible we compiled a query set of 237 publicly available sequences from Genbank, published papers and Repbase sequences [4]. These sequences cover all known retroviral genera and include both endogenous and exogenous strains from various host organisms (the query set is available upon request). Each query sequence was manually edited, removing LTR elements in order to avoid detection of solo LTRs. BLAST searches against contigs from the NCBI release 34 of the human genome were performed using WU-BLAST (Gish, W. (1996–2003) ), with default parameters except for W = 8, E = 0.001, V = 1000000, B = 1000000. Search results were stored in a MySql database and mapped to chromosomal positions using Ensembl Bioperl packages [61].

Overlapping BLAST hits were clustered into putative HERV regions allowing a gap of 500 nucleotides between hits. A region-score was calculated based on the sum of e-value weighted hitlengths divided by region length. Only regions longer than 300 nucleotides and a region-score > 3.0 (threshold based on empirical tests) were kept, resulting in 45658 putative HERV region.

Detection of direct flanking repeats (putative LTRs) were done by comparing a window before and after the HERV region.

### ORF finding and categorization

For the 45658 putative HERV loci, we scanned the DNA sequence (including 1000 bases flanking the locus) for forward open reading frames (stop-codon to stop-codon) of lengths > 62 aminoacids (aa). Stop-codon-to-stop-codon fragments were chosen to accommodate the use of non-conventional translational initiation by retroviruses at the internal *pro *and *pol *genes (by means of ribosomal frame-shifting and terminations suppression). Therefore the predicted proteins in particular for gag and env genes may contain incorrect N-terminal regions that must be removed by looking for appropriate start codons. ORF lengths below 63 aa were discarded as the probability of finding ORFs less than 63 aa in a random sequence increases to more than 0.05 (assuming equal codon frequencies).

All ORFs were then assigned to a category by FASTA searching against a library of known retroviral proteins (RV) and known non-retroviral proteins (NON_RV). RV proteins were downloaded from NCBI and categorized into either GAG, POL, PRO, ENV, ACC (accessory protein) or UNWANTED (for unwanted or unknown proteins). NON_RV proteins consists of all human SwissProt proteins of length 400–700 aa not including the words "endogenous, virus, envelope, env-, env, gag-, gag, pol-, pol, reverse". The final library consisted of 6260 records (3454 RV proteins + 2806 NON_RV proteins). ORF was assigned to the same category as the highest scoring hit. All loci with a significant RV ORF (vORF) were flagged as HERVs (E < 0.0005) – this data set consists of 7836 loci. Manual inspection of long ORF above 400 codons revealed that two envelope ORFs (ORF ID 86185 and 312172) were (mis)categorized as non-significant (NonS) due to low sequence similarity to our retroviral protein library.

### EST matching to individual proviruses

In order to match the human ESTs to the vORF positive HERV regions we first performed an all against all search using NCBI MegaBLAST [62]. The output was filtered so that only the best matching pairs (HERV-EST) were kept and put into a database. The ESTs that matched the HERV regions encompassing a long ORF were subsequently assigned to a human genomic region using EST mapping data from UCSC Genome Browser [63]. ESTs that unambiguously mapped to the same genomic region as the HERV regions of interest were counted as positive EST matches.

## List of abbreviations used

ERV Endogenous retrovirus

EST Expressed sequence tag

HERV Human endogenous retrovirus

LTR Long terminal repeat

vORF Viral open reading frame

## Competing interests

The authors declare that they have no competing interests.

## Authors' contributions

The study was conceived by LAA and FSP; PV and LAA participated in designing and coordinating the study; PV carried out all programming and compilation of , while LAA prepared query sequences, detailed analysis of the results and drafted the manuscript and PV and CW performed the statistical analysis. All authors read and approved the final manuscript.

## Supplementary Material

Additional File 1 Table 1. Genes with one or more vORF HERVs inside. Genes were selected from Ensembl (Current Release 21.34d.1) and compared with all HERV regions containing a retroviral ORF. 813 genes (642 with descriptions) contained 1182 HERVs (969) overlapping the gene chromosomal coordinates (exons + introns). The HERV score is a measure of the density of retroviral blast hits in the region. Click here for file

Additional File 2 Table 2. ESTs matching HERVs containing a long viral ORF. ESTs were compared to HERVs using megaBLAST. Only ESTs that best matched the target HERV were kept. Finally, ESTs mapping conclusively to the same genomic regions as the target HERV were kept. EST library information (organ and tissue) was parsed from Genbank. The positions are in NCBI35 coordinates due to overly stringent settings of EST mappings in the NCBI34 mapping at UCSC. HERV positions were lifted to NCBI35 coordinates using the "lift genome annotations" tool at Click here for file

## References

[B1] International Human Genome Sequencing Consortium (IHGSC) (2001). Initial sequencing and analysis of the human genome. Nature.

[B2] Tristem M (2000). Identification and characterization of novel human endogenous retrovirus families by phylogenetic screening of the Human Genome Mapping Project database. J Virol.

[B3] Benit L, Dessen P, Heidmann T (2001). Identification, phylogeny, and evolution of retroviral elements based on their envelope genes. J Virol.

[B4] Jurka J (2000). Repbase update: A database and an electronic journal of repetitive elements. Trends Genet.

[B5] Paces J, Pavlicek A, Zika R, Kapitonov VV, Jurka J, Paces V (2004). HERVd: the Human Endogenous RetroViruses Database: update. Nucleic Acids Res.

[B6] Boeke JD, Stoye JP, Coffin JM, Hughes SH, Varmus HE (1997). Retrotransposons, endogenous retroviruses, and the evolution of retroelements. In Retroviruses.

[B7] Herniou E, Martin J, Miller K, Cook J, Wilkinson M, Tristem M (1998). Retroviral diversity and distribution in vertebrates. J Virol.

[B8] Barbulescu M, Turner G, Seaman MI, Deinard AS, Kidd KK, Lenz J (1999). Many human endogenous retrovirus K (HERV-K) proviruses are unique to humans. Curr Biol.

[B9] Shih A, Coutavas EE, Rush MG (1991). Evolutionary implications of primate endogenous retroviruses. Virology.

[B10] Temin HM (1980). Origin of retroviruses from cellular moveable genetic elements. Cell.

[B11] Belshaw R, Pereira V, Katzourakis A, Talbot G, Paces J, Burt A, Tristem M (2004). Long-term reinfection of the human genome by endogenous retroviruses. Proc Natl Acad Sci USA.

[B12] Tchenio T, Heidmann T (1992). High-frequency intracellular transposition of a defective mammalian provirus detected by an in situ colorimetric assay. J Virol.

[B13] Lower R, Lower J, Kurth R (1996). The viruses in all of us: Characteristics and biological significance of human endogenous retrovirus sequences. Proc Natl Acad Sci USA.

[B14] Turner G, Barbulescu M, Su M, Jensen-Seaman MI, Kidd KK, Lenz J (2001). Insertional polymorphisms of full-length endogenous retroviruses in humans. Curr Biol.

[B15] Mikkelsen JG, Pedersen FS (2000). Genetic reassortment and patch repair by recombination in retroviruses. J Biomed Sci.

[B16] Rasmussen HB (1997). Interactions between exogenous and endogenous retroviruses. J Biomed Sci.

[B17] Kazazian HH (1999). An estimated frequency of endogenous insertional mutations in humans. Nat Genet.

[B18] Brosius J (1999). RNAs from all categories generate retrosequences that may be exapted as novel genes or regulatory elements. Gene.

[B19] Schneider PM, Witzel-Schlomp K, Rittner C, Zhang L (2001). The endogenous retroviral insertion in the human complement C4 gene modulates the expression of homologous genes by antisense inhibition. Immunogenetics.

[B20] Hughes JF, Coffin JM (2001). Evidence for genomic rearrangements mediated by human endogenous retroviruses during primate evolution. Nat Genet.

[B21] Magin C, Lower R, Lower J (1999). cORF and RcRE, the Rev/Rex and RRE/RxRE homologues of the human endogenous retrovirus family HTDV/HERV-K. J Virol.

[B22] Yang J, Bogerd HP, Peng S, Wiegand H, Truant R, Cullen BR (1999). An ancient family of human endogenous retroviruses encodes a functional homolog of the HIV-1 Rev protein. Proc Natl Acad Sci USA.

[B23] Lindeskog M, Mager DL, Blomberg J (1999). Isolation of a human endogenous retroviral HERV-H element with an open env reading frame. Virology.

[B24] Blond JL, Beseme F, Duret L, Bouton O, Bedin F, Perron H, Mandrand B, Mallet F (1999). Molecular characterization and placental expression of HERV-W, a new human endogenous retrovirus family. J Virol.

[B25] Cohen M, Powers M, Oconnell C, Kato N (1985). The nucleotide-sequence of the Env gene from the human provirus ERV3 and isolation and characterization of an ERV3-Specific cDNA. Virology.

[B26] Blond JL, Lavillette D, Cheynet V, Bouton O, Oriol G, Chapel-Fernandes S, Mandrand B, Mallet F, Cosset FL (2000). An envelope glycoprotein of the human endogenous retrovirus HERV-W is expressed in the human placenta and fuses cells expressing the type D mammalian retrovirus receptor. J Virol.

[B27] Mi S, Lee X, Li X, Veldman GM, Finnerty H, Racie L, LaVallie E, Tang XY, Edouard P, Howes S, Keith JC, McCoy JM (2000). Syncytin is a captive retroviral envelope protein involved in human placental morphogenesis. Nature.

[B28] Blaise S, de Parseval N, Benit L, Heidmann T (2003). Genomewide screening for fusogenic human endogenous retrovirus envelopes identifies syncytin 2, a gene conserved on primate evolution. Proc Natl Acad Sci USA.

[B29] Best S, Le Tissier P, Towers G, Stoye JP (1996). Positional cloning of the mouse retrovirus restriction gene Fv1. Nature.

[B30] Lower R (1999). The pathogenic potential of endogenous retroviruses: facts and fantasies. Trends Microbiol.

[B31] Boese A, Sauter M, Galli U, Best B, Herbst H, Mayer J, Kremmer E, Roemer K, Mueller-Lantzsch N (2000). Human endogenous retrovirus protein cORF supports cell transformation and associates with the promyelocytic leukemia zinc finger protein. Oncogene.

[B32] Mangeney M, de Parseval N, Thomas G, Heidmann T (2001). The full-length envelope of an HERV-H human endogenous retrovirus has immunosuppressive properties. J Gen Virol.

[B33] Conrad B, Weissmahr RN, Boni J, Arcari R, Schupbach J, Mach B (1997). A human endogenous retroviral superantigen as candidate autoimmune gene in type I diabetes. Cell.

[B34] Perron H, Jouvin-Marche E, Michel M, Ounanian-Paraz A, Camelo S, Dumon A, Jolivet-Reynaud C, Marcel F, Souillet Y, Borel E, Gebuhrer L, Santoro L, Marcel S, Seigneurin JM, Marche PN, Lafon M (2001). Multiple sclerosis retrovirus particles and recombinant envelope trigger an abnormal immune response in vitro, by inducing polyclonal Vbeta16 T-lymphocyte activation. Virology.

[B35] Gaudin P, Ijaz S, Tuke PW, Marcel F, Paraz A, Seigneurin JM, Mandrand B, Perron H, Garson JA (2000). Infrequency of detection of particle-associated MSRV/HERV-W RNA in the synovial fluid of patients with rheumatoid arthritis. Rheumatology.

[B36] Karlsson H, Bachmann S, Schroder J, McArthur J, Torrey EF, Yolken RH (2001). Retroviral RNA identified in the cerebrospinal fluids and brains of individuals with schizophrenia. Proc Natl Acad Sci USA.

[B37] Stauffer Y, Marguerat S, Meylan F, Ucla C, Sutkowski N, Huber B, Pelet T, Conrad B (2001). Interferon-alpha-induced endogenous superantigen. a model linking environment and autoimmunity. Immunity.

[B38] Sutkowski N, Conrad B, Thorley-Lawson DA, Huber BT (2001). Epstein-Barr virus transactivates the human endogenous retrovirus HERV-K18 that encodes a superantigen. Immunity.

[B39] Cianciolo GJ, Copeland TD, Oroszlan S, Snyderman R (1985). Inhibition of lymphocyte proliferation by a synthetic peptide homologous to retroviral envelope proteins. Science.

[B40] Jern P, Sperber GO, Blomberg J (2004). Definition and variation of human endogenous retrovirus H. Virology.

[B41] de Parseval N, Lazar V, Casella JF, Benit L, Heidmann T (2003). Survey of human genes of retroviral origin: Identification and transcriptome of the genes with coding capacity for complete envelope proteins. J Virol.

[B42] Paces J, Pavlicek A, Paces V (2002). HERVd: database of human endogenous retroviruses. Nucleic Acids Res.

[B43] Human endogenous retrovirus database. http://herv.img.cas.cz.

[B44] Reus K, Mayer J, Sauter M, Zischler H, Muller-Lantzsch N, Meese E (2001). Genomic organization of the human endogenous retrovirus HERV-K(HML-2.HOM) (ERVK6) on chromosome 7. Genomics.

[B45] Benit L, Calteau A, Heidmann T (2003). Characterization of the low-copy HERV-Fc family: evidence for recent integrations in primates of elements with coding envelope genes. Virology.

[B46] Shen CH, Steiner LA (2004). Genome structure and thymic expression of an endogenous retrovirus in zebrafish. J Virol.

[B47] RetroSearch: database of ORF annotated human endogenous retroviruses. http://www.retrosearch.dk.

[B48] Frendo JL, Olivier D, Cheynet V, Blond JL, Bouton O, Vidaud M, Rabreau M, Evain-Brion D, Mallet F (2003). Direct involvement of HERV-W Env glycoprotein in human trophoblast cell fusion and differentiation. Mol Cell Biol.

[B49] Bonnaud B, Bouton O, Oriol G, Cheynet V, Duret L, Mallet F (2004). Evidence of Selection on the Domesticated ERVWE1 env Retroviral Element Involved in Placentation. Mol Biol Evol.

[B50] Mallet F, Bouton O, Prudhomme S, Cheynet V, Oriol G, Bonnaud B, Lucotte G, Duret L, Mandrand B (2004). The endogenous retroviral locus ERVWE1 is a bona fide gene involved in hominoid placental physiology. Proc Natl Acad Sci USA.

[B51] Gardner MB, Kozak CA, O'Brien SJ (1991). The Lake Casitas wild mouse: evolving genetic resistance to retroviral disease. Trends Genet.

[B52] Harris JR (1998). Placental endogenous retrovirus (ERV): structural, functional, and evolutionary significance. Bioessays.

[B53] Lower R, Lower J, Tondera-Koch C, Kurth R (1993). A general method for the identification of transcribed retrovirus sequences (R-U5 PCR) reveals the expression of the human endogenous retrovirus loci HERV-H and HERV-K in teratocarcinoma cells. Virology.

[B54] Costas J (2001). Evolutionary dynamics of the human endogenous retrovirus family HERV-K inferred from full-length proviral genomes. J Mol Evol.

[B55] Kjellman C, Sjogren HO, Widegren B (1995). The Y-chromosome – a graveyard for endogenous retroviruses. Gene.

[B56] Medstrand P, van de Lagemaat LN, Mager DL (2002). Retroelement distributions in the human genome: variations associated with age and proximity to genes. Genome Res.

[B57] Pavlicek A, Paces J, Elleder D, Hejnar J (2002). Processed pseudogenes of human endogenous retroviruses generated by LINEs: their integration, stability, and distribution. Genome Res.

[B58] Smit AF (1999). Interspersed repeats and other mementos of transposable elements in mammalian genomes. Curr Opin Genet Dev.

[B59] Mitchell RS, Beitzel BF, Schroder AR, Shinn P, Chen H, Berry CC, Ecker JR, Bushman FD (2004). Retroviral DNA Integration: ASLV, HIV, and MLV Show Distinct Target Site Preferences. PLoS Biol.

[B60] Schroder AR, Shinn P, Chen H, Berry C, Ecker JR, Bushman F (2002). HIV-1 integration in the human genome favors active genes and local hotspots. Cell.

[B61] Ensembl Genome Browser. http://www.ensembl.org.

[B62] Zhang Z, Schwartz S, Wagner L, Miller W (2000). A greedy algorithm for aligning DNA sequences. J Computational Biology.

[B63] UCSC Genome Browser. http://genome.ucsc.edu.

