# Supplementary material for: Identification of endogenous retroviral reading frames in the human genome
Source: Retrovirology. 2004 Oct 11;1:32. doi: 10.1186/1742-4690-1-32 (PMC524368; doi:10.1186/1742-4690-1-32)
Supplement: Additional File 1 — Table 1. Genes with one or more vORF HERVs inside. Genes were selected from Ensembl (Current Release 21.34d.1) and compared with all HERV regions containing a retroviral ORF. 813 genes (642 with descriptions) contained 1182 HERVs (969) overlapping the gene chromosomal coordinates (exons + introns). The HERV score is a measure of the density of retroviral blast hits in the region. [file 1742-4690-1-32-S1.pdf]

Additional File 1

Table 1. Genes with one or more vORF HERVs inside.

Genes were selected from Ensembl (Current Release 21.34d.1) and compared with all HERV regions containing a retroviral ORF. 813 genes (642 with descriptions) contained 1182 HERVs (969) overlapping the gene chromosomal coordinates (exons + introns). The HERV score is a measure of the density of retroviral blast hits in the region.

Chr	Ensembl id	Description	Strand	HERV id	HERV score
1	ENSG00000000971.3	"Complement factor H precursor (H factor 1). [Source:SWISSPROT;Acc:P08603]"	same	6119	4.95864
1				6120	12.9745
1	ENSG00000048707.2	"vacuolar protein sorting 13D. [Source:RefSeq;Acc:NM_015378]"	opposite	6468	5.53156
1				6469	8.632
1	ENSG00000051415.5	Alpha-amylase	same	5459	21.7604
1			opposite	7018	22.2868
1	ENSG00000057468.1	"MutS protein homolog 4. [Source:SWISSPROT;Acc:O15457]"	same	5207	3.74618
1	ENSG00000067208.3	"EVI-5 homolog. [Source:SPTREMBL;Acc:O60447]"	opposite	5363	4.4899
1	ENSG00000075391.4	"Ras GTPase-activating protein nGAP (RAS protein activator like 1). [Source:SWISSPROT;Acc:Q9UJF2]"	same	5846	5.08548
1			opposite	7413	13.9851
1	ENSG00000078900.2	"Tumor protein p73 (p53-like transcription factor) (p53-related protein). [Source:SWISSPROT;Acc:O15350]"	same	4897	3.0776
1			opposite	6449	12.333
1	ENSG00000116141.5	"MAP/microtubule affinity-regulating kinase 1. [Source:RefSeq;Acc:NM_018650]"	opposite	7796	5.5134
1	ENSG00000116711.2	"Cytosolic phospholipase A2 (CPLA2) [Includes: Phospholipase A2 (EC 3.1.1.4) (Phosphatidylcholine 2-acylhydrolase); Lysophospholipase (EC 3.1.1.5)]. [Source:SWISSPROT;Acc:P47712]"	opposite	7447	26.2564
1	ENSG00000117020.4	RAC-gamma serine/threonine protein kinase (EC 2.7.1.-) (RAC-PK-gamma) (Protein kinase Akt-3) (Protein kinase B	opposite	6400	1.64645
1	ENSG00000117069.3	Alpha-N-acetylgalactosaminide alpha-2	opposite	6772	7.2391
1				6773	14.7155
1	ENSG00000117091.1	"B-lymphocyte activation marker BLAST-1 precursor (BCM1 surface antigen) (Leucocyte antigen MEM-102) (TCT.1) (Antigen CD48). [Source:SWISSPROT;Acc:P09326]"	same	7300	15.0697
1				7301	22.0265
1			opposite	5715	22.3467
1	ENSG00000117322.5	"Complement decay-accelerating factor precursor (CD55 antigen). [Source:SWISSPROT;Acc:P08174]"	opposite	7747	2.55454
1				7749	16.3125
1	ENSG00000117501.3	(blank)	opposite	7361	5.3024
1	ENSG00000117528.1	ATP-binding cassette	same	5371	20.8658
1	ENSG00000121904.5	"CUB and sushi multiple domains protein 2. [Source:SWISSPROT;Acc:Q7Z408]"	opposite	4986	4.51576
1	ENSG00000123838.1	"C4b-binding protein alpha chain precursor (C4bp) (Proline-rich protein) (PRP). [Source:SWISSPROT;Acc:P04003]"	opposite	7743	7.05896
1				7744	3.58426
1	ENSG00000132122.1	"spermatogenesis associated 6. [Source:RefSeq;Acc:NM_019073]"	opposite	5037	13.7339
1	ENSG00000132676.2	"Mitochondrial 28S ribosomal protein S29 (S29mt) (MRP-S29) (Death- associated protein 3) (DAP-3) (Ionizing radiation resistance	opposite	7267	19.8556

Chr	Ensembl id	Description	Strand	HERV id	HERV score
		conferring protein). [Source:SWISSPROT;Acc:P51398]"			
1				7268	22.8178
1	ENSG00000134250.5	"Neurogenic locus notch homolog protein 2 precursor (Notch 2) (hN2). [Source:SWISSPROT;Acc:Q04721]"	opposite	5612	3.38852
1	ENSG00000134263.3	"Synaptonemal complex protein 1 (SCP-1 protein). [Source:SWISSPROT;Acc:Q15431]"	opposite	7132	6.20943
1	ENSG00000134716.2	"Cytochrome P450 2J2 (EC 1.14.14.1) (CYPIIJ2) (Arachidonic acid epoxygenase). [Source:SWISSPROT;Acc:P51589]"	same	6625	1.28276
1	ENSG00000135747.3	"Zinc finger protein 124 (HZF-16). [Source:SWISSPROT;Acc:Q15973]"	opposite	6419	19.177
1				6421	11.2121
1	ENSG00000137968.3	(blank)	opposite	5201	13.7351
1	ENSG00000142623.1	"Protein-arginine deiminase type I (EC 3.5.3.15) (Peptidylarginine deiminase I). [Source:SWISSPROT;Acc:Q9ULC6]"	opposite	6486	5.6369
1	ENSG00000142892.3	GPI-anchor transamidase (EC 3.-.-.-) (GPI transamidase) (Phosphatidylinositol-glycan biosynthesis	opposite	5218	7.02944
1	ENSG00000143151.4	(blank)	opposite	7334	17.7782
1	ENSG00000143190.5	POU domain	opposite	7339	23.4733
1	ENSG00000143199.4	"soluble adenylyl cyclase; adenylate cyclase; ATP pyrophosphate-lyase; 3'"	opposite	5763	8.07764
1	ENSG00000143207.5	"constitutive photomorphogenic protein. [Source:RefSeq;Acc:NM_022457]"	opposite	5836	8.4055
1	ENSG00000143341.2	"hemicentin; fibulin 6. [Source:RefSeq;Acc:NM_031935]"	opposite	7442	12.8011
1	ENSG00000143344.3	"Ral guanine nucleotide dissociation stimulator-like 1 (RalGDS-like 1). [Source:SWISSPROT;Acc:Q9NZL6]"	same	5863	4.50732
1			opposite	7433	21.9469
1	ENSG00000143457.1	"GPP34-related protein. [Source:RefSeq;Acc:NM_018178]"	opposite	5668	24.7978
1	ENSG00000143469.2	"synaptotagmin XIV. [Source:RefSeq;Acc:NM_153262]"	opposite	7758	15.534
1	ENSG00000143552.1	(blank)	opposite	5683	18.1017
1	ENSG00000143653.2	(blank)	opposite	7928	8.00952
1	ENSG00000143669.1	"Lysosomal trafficking regulator (Beige homolog). [Source:SWISSPROT;Acc:Q99698]"	opposite	6353	3.84261
1	ENSG00000143850.1	"phosphoinositol 3-phosphate-binding protein-3. [Source:RefSeq;Acc:NM_014935]"	opposite	6161	7.20463
1	ENSG00000152763.4	(blank)	same	6647	5.02799
1	ENSG00000153936.5	"heparan sulfate 2-O-sulfotransferase 1. [Source:RefSeq;Acc:NM_012262]"	same	5330	7.39945
1	ENSG00000154198.3	(blank)	opposite	5024	3.40297
1	ENSG00000154358.5	obscurin	opposite	7846	7.27418
1	ENSG00000154370.2	"Tripartite motif protein 11 (BIA1 protein). [Source:SWISSPROT;Acc:Q96F44]"	same	7846	7.27418
1	ENSG00000157077.3	"Mothers against decapentaplegic homolog interacting protein (Madh-interacting protein) (Smad anchor for receptor activation) (Receptor activation anchor) (hSARA) (Novel serine protease) (NSP). [Source:SWISSPROT;Acc:O95405]"	opposite	6593	20.1584
1	ENSG00000162374.4	"ELAV-like protein 4 (Paraneoplastic encephalomyelitis antigen HuD) (Hu-antigen D). [Source:SWISSPROT;Acc:P26378]"	opposite	6589	3.89602
1	ENSG00000162390.5	"Brown fat inducible thioesterase (EC 3.1.2.-) (BFIT) (Adipose associated thioesterase). [Source:SWISSPROT;Acc:Q8WXI4]"	same	5078	15.1492
1	ENSG00000162618.1	"ETL protein (EGF-TM7-latrophilin-related protein). [Source:SPTREMBL;Acc:Q9HBW9]"	opposite	5240	7.45848
1	ENSG00000162623.3	(blank)	opposite	6754	12.549
1	ENSG00000162779.5	(blank)	opposite	7418	9.67356
1	ENSG00000162787.3	"regulator of G-protein signalling like 1. [Source:RefSeq;Acc:NM_181572]"	opposite	7430	4.02892
1	ENSG00000162852.4	(blank)	opposite	7925	7.23395
1				7926	9.36071
1				7927	50.514
1	ENSG00000162901.1	(blank)	same	7747	2.55454
1	ENSG00000162946.4	"Disrupted in schizophrenia 1 protein. [Source:SWISSPROT;Acc:Q9NRI5]"	opposite	7854	18.5469
1	ENSG00000163386.4	(blank)	opposite	7208	2.86689
1	ENSG00000163564.2	"interferon-inducible protein X alpha 1 isoform. [Source:RefSeq;Acc:NM_152501]"	opposite	7291	7.29365
1				7292	4.38674

Chr	Ensembl id	Description	Strand	HERV id	HERV score
1	ENSG00000163565.4	"Gamma-interferon-inducible protein Ifi-16 (Interferon-inducible myeloid differentiation transcriptional activator) (IFI 16). [Source:SWISSPROT;Acc:Q16666]"	opposite	7293	5.363
1	ENSG00000171502.3	collagen	opposite	5306	3.37317
1				5311	4.62643
1	ENSG00000171621.4	"SPRY domain-containing SOCS box protein SSB-1. [Source:RefSeq;Acc:NM_025106]"	same	4918	4.7225
1				4919	4.07078
1	ENSG00000171967.4	(blank)	opposite	6542	6.33719
1	ENSG00000172260.2	"neuronal growth regulator 1; a kindred of IgLON. [Source:RefSeq;Acc:NM_173808]"	opposite	5146	11.05
1				5151	3.97414
1	ENSG00000173662.5	"Krueppel-related zinc finger protein 3 (HKR3 protein). [Source:SWISSPROT;Acc:P10074]"	opposite	6460	4.69689
1				6461	17.2918
1	ENSG00000175511.4	"3 beta-hydroxysteroid dehydrogenase/delta 5-->4-isomerase type I (3Beta-HSD I) (Trophoblast antigen FDO161G) [Includes: 3-beta-hydroxy- delta(5)-steroid dehydrogenase (EC 1.1.1.145) (3-beta-hydroxy-5-ene steroid dehydrogenase) (Progesterone reductase); St"	opposite	7161	13.1115
1				7162	3.73029
1	ENSG00000175709.4	"Zinc finger protein 73 (Zinc finger protein 186) (hZNF2). [Source:SWISSPROT;Acc:O43830]"	opposite	5618	11.5428
1	ENSG00000177275.2	"Seven transmembrane helix receptor. [Source:SPTREMBL;Acc:Q8NGZ0]"	opposite	7940	11.2885
1	ENSG00000177381.2	"Seven transmembrane helix receptor. [Source:SPTREMBL;Acc:Q8NGY8]"	opposite	7938	5.35657
1	ENSG00000177462.2	"Olfactory receptor 5AV1. [Source:SWISSPROT;Acc:Q8NHC6]"	opposite	7936	7.18044
1				7938	5.35657
1				7940	11.2885
1	ENSG00000177535.3	"Olfactory receptor 2C3. [Source:SWISSPROT;Acc:Q8N628]"	same	7935	0.970044
1				7936	7.18044
1				7938	5.35657
1			opposite	6429	4.97612
1	ENSG00000178961.3	"TNNI3 interacting kinase; cardiac ankyrin repeat kinase. [Source:RefSeq;Acc:NM_015978]"	same	5192	6.574
1	ENSG00000179397.4	(blank)	same	6406	8.26898
1				6407	4.98223
1	ENSG00000180287.4	(blank)	opposite	6386	15.1809
1				6389	11.7697
1	ENSG00000183682.2	"Bone morphogenetic protein 8B precursor (BMP-8B) (BMP-8) (Osteogenic protein 2) (OP-2). [Source:SWISSPROT;Acc:P34820]"	same	4999	0.915857
1	ENSG00000183831.1	(blank)	opposite	5812	10.1077
1	ENSG00000184155.3	"Olfactory receptor 10J5. [Source:SWISSPROT;Acc:Q8NHC4]"	same	7295	5.21188
1			opposite	5713	4.6983
1				5714	4.62897
1	ENSG00000184588.4	cAMP-specific 3'	opposite	6643	5.08327
1	ENSG00000186140.3	"myelin protein zero-like 1; protein zero related. [Source:RefSeq;Acc:NM_003953]"	opposite	7343	3.86411
1				7344	4.39683
1	ENSG00000186160.1	"cytochrome P450 4Z1. [Source:RefSeq;Acc:NM_178134]"	opposite	6575	3.40258
1	ENSG00000187080.2	"Olfactory receptor 2AK2. [Source:SWISSPROT;Acc:Q8NG84]"	opposite	7945	3.01645
1				7946	3.25452
1				7947	3.08341
1				7949	13.9709
1	ENSG00000187481.1	"3 beta-hydroxysteroid dehydrogenase (Fragment). [Source:SPTREMBL;Acc:Q9UD07]"	opposite	7162	3.73029
2	ENSG00000003436.3	"Tissue factor pathway inhibitor precursor (TFPI) (Lipoprotein- associated coagulation inhibitor) (LACI) (Extrinsic pathway inhibitor) (EPI).	opposite	9349	9.76648

Chr	Ensembl id	Description	Strand	HERV id	HERV score
		[Source:SWISSPROT;Acc:P10646]"			
2	ENSG00000018510.2	Alkyldihydroxyacetonephosphate synthase	opposite	10875	6.93216
2	ENSG00000019169.2	"Macrophage receptor MARCO (Macrophage receptor with collagenous structure). [Source:SWISSPROT;Acc:Q9UEW3]"	opposite	10478	6.70417
2	ENSG00000028116.2	"Serine/threonine protein kinase VRK2 (EC 2.7.1.37) (Vaccinia-related kinase 2). [Source:SWISSPROT;Acc:Q86Y07]"	opposite	10066	12.4216
2	ENSG00000030419.3	"Zinc finger protein Helios. [Source:SWISSPROT;Acc:Q9UKS7]"	opposite	9609	3.96927
2	ENSG00000049323.1	Latent transforming growth factor beta binding protein	opposite	9887	15.0095
2	ENSG00000055332.3	Interferon-induced	opposite	8163	20.3552
2	ENSG00000064036.4	"Bone morphogenetic protein receptor type II precursor (EC 2.7.1.37) (BMP type II receptor) (BMPR-II). [Source:SWISSPROT;Acc:Q13873]"	same	9534	21.7813
2	ENSG00000066032.4	"Alpha-2 catenin (Alpha-catenin related protein) (Alpha N-catenin). [Source:SWISSPROT;Acc:P26232]"	opposite	10195	5.8346
2	ENSG00000068615.4	(blank)	opposite	8535	2.10288
2	ENSG00000071909.4	"myosin IIIB. [Source:RefSeq;Acc:NM_138995]"	opposite	10854	9.36172
2				10857	10.7429
2	ENSG00000075292.2	"NP220 nuclear protein; CTCL tumor antigen se33-1. [Source:RefSeq;Acc:NM_014497]"	same	8371	16.5015
2	ENSG00000075568.3	"RW1 protein (Fragment). [Source:SWISSPROT;Acc:Q92545]"	opposite	8588	22.7733
2				8589	15.622
2	ENSG00000077232.3	"ER-resident protein ERdj5; macrothioredoxin; J-domain-containing protein disulfide isomerase-like protein. [Source:RefSeq;Acc:NM_018981]"	opposite	10899	2.91039
2				10900	8.63685
2	ENSG00000081479.3	"Low-density lipoprotein receptor-related protein 2 precursor (Megalin) (Glycoprotein 330) (gp330). [Source:SWISSPROT;Acc:P98164]"	opposite	9159	12.4373
2	ENSG00000084754.1	Trifunctional enzyme alpha subunit	opposite	8102	5.0564
2	ENSG00000088179.1	Protein tyrosine phosphatase	opposite	10483	3.29305
2	ENSG00000091436.5	"mixed lineage kinase-related kinase MRK-beta; mixed lineage kinase with a leucine zipper and a sterile alpha motif; mixed lineage kinase-related kinase. [Source:RefSeq;Acc:NM_133646]"	opposite	10867	2.54379
2	ENSG00000115020.5	"FYVE finger-containing phosphoinositide kinase (EC 2.7.1.68) (1- phosphatidylinositol-4-phosphate 5-kinase) (PIP5K) (PtdIns(4)P-5-kinase) (p235) (Fragment). [Source:SWISSPROT;Acc:Q9Y2I7]"	opposite	11149	4.73584
2	ENSG00000115239.5	"Ankyrin repeat and SOCS box containing protein 3 (ASB-3). [Source:SWISSPROT;Acc:Q9Y575]"	opposite	8280	5.38243
2	ENSG00000115252.5	Calcium/calmodulin-dependent 3'	opposite	9233	6.18146
2				9234	3.20005
2				9235	2.23039
2				9236	6.9653
2	ENSG00000115339.2	"polypeptide N-acetylgalactosaminyltransferase 3; protein-UDP acetylgalactosaminyltransferase. [Source:RefSeq;Acc:NM_004482]"	same	10818	3.53648
2	ENSG00000115669.4	"Sulfotransferase 1C1 (EC 2.8.2.-) (SULT1C#1) (ST1C2) (humSULTC2). [Source:SWISSPROT;Acc:O00338]"	opposite	10410	7.66806
2				10411	3.17108
2	ENSG00000115760.1	"Baculoviral IAP repeat-containing protein 6 (Ubiquitin-conjugating BIR-domain enzyme apollon). [Source:SWISSPROT;Acc:Q9NR09]"	opposite	9884	3.26139
2	ENSG00000115840.2	Calcium-binding mitochondrial carrier protein Aralar1 (Mitochondrial aspartate glutamate carrier 1) (Solute carrier family 25	opposite	9174	4.52661
2				9175	4.60236
2	ENSG00000115919.2	"Kynureninase (EC 3.7.1.3) (L-kynurenine hydrolase). [Source:SWISSPROT;Acc:Q16719]"	opposite	10640	6.0457
2				10641	17.6009
2				10642	21.5473
2	ENSG00000115980.2	"Annexin A4 (Annexin IV) (Lipocortin IV) (Endonexin I) (Chromobindin 4) (Protein II) (P32.5) (Placental anticoagulant protein II) (PAP-II) (PP4-X) (35-beta calcimedin) (Carbohydrate-binding protein P33/P41) (P33/41). [Source:SWISSPROT;Acc:P09525]"	opposite	10105	9.68452
2	ENSG00000118965.3	"WD repeat domain 35. [Source:RefSeq;Acc:NM_020779]"	opposite	8073	15.0479
2	ENSG00000123596.5	"Formin binding protein 3 (Huntingtin yeast partner A) (Huntingtin- interacting protein HYPA/FBP11) (Fas-ligand associated factor 1) (NY-	opposite	8986	15.3264

Chr	Ensembl id	Description	Strand	HERV id	HERV score
		REN-6 antigen) (HSPC225). [Source:SWISSPROT;Acc:O75400]"			
2	ENSG00000135929.1	Cytochrome P450 27	opposite	11242	5.44303
2	ENSG00000138081.5	"F-box only protein 11 (Vitiligo-associated protein VIT-1). [Source:SWISSPROT;Acc:Q86XK2]"	opposite	8194	4.6315
2	ENSG00000138400.2	(blank)	opposite	9543	4.27293
2	ENSG00000143797.2	(blank)	same	9788	21.048
2	ENSG00000143951.5	(blank)	same	10083	4.46253
2			opposite	8333	3.81265
2				8335	11.8262
2	ENSG00000144039.4	"similar to POSSIBLE GUSTATORY RECEPTOR CLONE PTE01. [Source:RefSeq;Acc:NM_145242]"	same	8369	35.2145
2				8370	23.1174
2	ENSG00000144229.1	(blank)	same	8868	7.55411
2	ENSG00000144278.2	"UDP-N-acetyl-alpha-D-galactosamine:polypeptide N-acetylgalactosaminyltransferase 13; GalNAc transferase 13. [Source:RefSeq;Acc:NM_052917]"	opposite	10733	1.63746
2				10736	5.87998
2	ENSG00000144290.1	solute carrier family 4	opposite	10788	1.94963
2				10789	7.30948
2	ENSG00000144331.5	"zinc finger protein 533. [Source:RefSeq;Acc:NM_152520]"	opposite	9200	2.40279
2	ENSG00000144339.1	"transmembrane protein with EGF-like and two follistatin-like domains 2; transmembrane protein TENB2; tomoregulin; putative transmembrane protein with EGF-like and two follistatin-like domains 2. [Source:RefSeq;Acc:NM_016192]"	opposite	9403	5.93519
2				9404	11.4631
2	ENSG00000144362.2	(blank)	opposite	10852	2.91819
2	ENSG00000144366.5	GULP	opposite	11002	2.16342
2	ENSG00000144451.4	"PF20; sperm-associated WD repeat protein; WD repeat domain 29. [Source:RefSeq;Acc:NM_024532]"	same	9612	4.24042
2				9615	9.56672
2			opposite	11208	4.61491
2				11209	4.1076
2				11215	11.146
2	ENSG00000144452.3	ATP-binding cassette	opposite	9622	18.5713
2	ENSG00000152256.1	[Pyruvate dehydrogenase [lipoamide]] kinase isozyme 1	opposite	10863	9.98566
2	ENSG00000153107.3	"Anaphase promoting complex subunit 1 (APC1) (Cyclosome subunit 1) (Protein Tsg24) (Mitotic checkpoint regulator). [Source:SWISSPROT;Acc:Q9H1A4]"	opposite	8671	7.9406
2	ENSG00000155754.3	(blank)	same	11139	5.25937
2	ENSG00000157985.4	"Centaurin gamma 2. [Source:SWISSPROT;Acc:Q9UPQ3]"	opposite	11307	5.68328
2	ENSG00000162944.1	(blank)	opposite	9522	23.7498
2				9523	9.85501
2	ENSG00000163067.3	"zinc finger protein 2. [Source:RefSeq;Acc:NM_021088]"	opposite	10344	5.3431
2				10347	1.35124
2	ENSG00000163082.4	"Sphingosine-1-phosphate phosphatase 2 (EC 3.1.3.-) (Sphingosine-1- phosphatase 2) (SPPase2) (Spp2) (hSPP2). [Source:SWISSPROT;Acc:Q8IWX5]"	opposite	11256	7.22338
2	ENSG00000163464.2	"High affinity interleukin-8 receptor A (IL-8R A) (IL-8 receptor type 1) (CXCR-1) (CDw128a). [Source:SWISSPROT;Acc:P25024]"	same	11237	24.8766
2				11238	19.8714
2			opposite	9642	12.7854
2	ENSG00000168702.4	"low density lipoprotein-related protein 1B; low density lipoprotein receptor related protein-deleted in tumor. [Source:RefSeq;Acc:NM_018557]"	opposite	8904	19.0724

Chr	Ensembl id	Description	Strand	HERV id	HERV score
2				8907	3.70431
2	ENSG00000170396.2	(blank)	opposite	10926	3.58223
2	ENSG00000172071.1	"Eukaryotic translation initiation factor 2-alpha kinase 3 precursor (EC 2.7.1.-) (PRKR-like endoplasmic reticulum kinase) (Pancreatic eIF2-alpha kinase) (HsPEK). [Source:SWISSPROT;Acc:Q9NZJ5]"	opposite	8546	3.61768
2	ENSG00000172554.3	"Gamma-2-syntrophin (G2SYN) (Syntrophin 5) (SYN5). [Source:SWISSPROT;Acc:Q9NY99]"	opposite	9749	3.02119
2	ENSG00000172954.2	"HSRG1849. [Source:RefSeq;Acc:NM_182551]"	same	8111	7.36761
2	ENSG00000173163.1	"MURR1; copper metabolism gene MURR1; chromosome 2 open reading frame 5 (MURR1). [Source:RefSeq;Acc:NM_152516]"	opposite	10075	2.50642
2				10077	17.7549
2				10078	7.81869
2	ENSG00000173692.1	"26S proteasome non-ATPase regulatory subunit 1 (26S proteasome regulatory subunit S1) (26S proteasome subunit p112). [Source:SWISSPROT;Acc:Q99460]"	opposite	11298	10.8286
2	ENSG00000175497.4	"dipeptidylpeptidase 10; dipeptidyl peptidase IV-related protein 3. [Source:RefSeq;Acc:NM_020868]"	opposite	10438	3.10468
2				10439	5.70719
2				10441	7.43805
2	ENSG00000175701.1	(blank)	opposite	8663	0.966058
2	ENSG00000175772.4	(blank)	opposite	10420	4.05274
2	ENSG00000176120.2	(blank)	opposite	8658	8.56404
2	ENSG00000178568.4	"Receptor protein-tyrosine kinase erbB-4 precursor (EC 2.7.1.112) (p180erbB4) (Tyrosine kinase-type cell surface receptor HER4). [Source:SWISSPROT;Acc:Q15303]"	opposite	9607	6.36343
2	ENSG00000183840.2	"Putative G protein-coupled receptor GPR39. [Source:SWISSPROT;Acc:O43194]"	opposite	10578	3.54725
2	ENSG00000188941.1	"Nonhistone chromosomal protein HMG-14 (High-mobility group nucleosome binding domain 1). [Source:SWISSPROT;Acc:P05114]"	same	8589	15.622
3	ENSG00000008226.5	"deleted in lung and esophageal cancer 1 isoform DLEC1-S2; deleted in lung cancer 1. [Source:RefSeq;Acc:NM_007336]"	opposite	13250	17.2349
3	ENSG00000047849.5	"Microtubule-associated protein 4 (MAP 4). [Source:SWISSPROT;Acc:P27816]"	opposite	11587	3.88789
3				11588	6.16058
3	ENSG00000058705.4	"Interleukin-1 receptor accessory protein precursor (IL-1 receptor accessory protein) (IL-1RAcP). [Source:SWISSPROT;Acc:Q9NPH3]"	opposite	14695	8.42737
3	ENSG00000073803.2	"mitogen-activated protein kinase kinase kinase 13; leucine zipper-bearing kinase. [Source:RefSeq;Acc:NM_004721]"	opposite	14673	19.0735
3	ENSG00000075711.4	Presynaptic protein SAP97 (Synapse-associated protein 97) (Discs	same	14736	9.33089
3	ENSG00000082701.3	"Glycogen synthase kinase-3 beta (EC 2.7.1.37) (GSK-3 beta). [Source:SWISSPROT;Acc:P49841]"	opposite	12311	5.27889
3				12312	6.37235
3	ENSG00000088538.4	"Dedicator of cytokinesis protein 3 (Modifier of cell adhesion) (Preselinin binding protein) (PBP protein). [Source:SWISSPROT;Acc:Q8IZD9]"	opposite	13320	4.96073
3	ENSG00000113966.2	"ADP-ribosylation factor-like protein 6. [Source:SWISSPROT;Acc:Q9H0F7]"	opposite	13786	2.88018
3	ENSG00000114127.2	"strand-exchange protein 1. [Source:RefSeq;Acc:NM_019001]"	opposite	12426	22.0743
3	ENSG00000114439.3	"HMG-BOX transcription factor BBX; x 001 protein. [Source:RefSeq;Acc:NM_020235]"	opposite	13927	7.63301
3	ENSG00000114670.2	"NIMA (never in mitosis gene a)- related kinase 11. [Source:RefSeq;Acc:NM_145910]"	opposite	14073	13.7934
3	ENSG00000114757.5	"PXR2b protein. [Source:RefSeq;Acc:NM_016559]"	opposite	12909	7.31341
3	ENSG00000118007.3	"Cohesin subunit SA-1 (Stromal antigen 1) (SCC3 homolog 1). [Source:SWISSPROT;Acc:Q8WVM7]"	opposite	12404	4.06128
3	ENSG00000120742.1	"stress-associated endoplasmic reticulum protein 1; ribosome associated membrane protein 4. [Source:RefSeq;Acc:NM_014445]"	opposite	12537	9.05524
3	ENSG00000134108.1	"ADP-ribosylation factor-like 10C. [Source:RefSeq;Acc:NM_018184]"	opposite	13023	16.3542
3	ENSG00000134115.2	"contactin 6; neural adhesion molecule. [Source:RefSeq;Acc:NM_014461]"	opposite	13002	17.1454
3	ENSG00000136523.4	Dynamin-like 120 kDa protein	opposite	14734	8.11423
3	ENSG00000144893.2	"TRALPUSH; no opposite paired repeat protein. [Source:RefSeq;Acc:NM_053002]"	opposite	14224	3.75331
3	ENSG00000144908.2	"10-formyltetrahydrofolate dehydrogenase (EC 1.5.1.6) (10-FTHFDH). [Source:SWISSPROT;Acc:O75891]"	opposite	12348	6.70428
3	ENSG00000144959.1	(blank)	opposite	12828	3.82664

Chr	Ensembl id	Description	Strand	HERV id	HERV score
3	ENSG00000144974.5	(blank)	same	14267	4.4094
3	ENSG00000145087.3	(blank)	opposite	14017	2.66834
3				14019	22.0974
3				14035	19.2651
3	ENSG00000151967.5	"schwannomin interacting protein 1. [Source:RefSeq;Acc:NM_014575]"	opposite	14280	5.59543
3	ENSG00000156931.2	(blank)	opposite	14666	13.2359
3				14667	23.9932
3	ENSG00000157388.2	Voltage-dependent L-type calcium channel alpha-1D subunit (Calcium channel	opposite	13328	3.18688
3	ENSG00000157445.4	calcium channel	opposite	13333	12.4605
3	ENSG00000163610.3	(blank)	same	13984	3.11062
3			opposite	12255	8.11777
3	ENSG00000163617.1	(blank)	opposite	12258	3.85945
3	ENSG00000163630.1	"Synaptoporin. [Source:SWISSPROT;Acc:Q8TBG9]"	opposite	13360	4.20588
3	ENSG00000163669.2	(blank)	same	13344	1.39985
3			opposite	11623	13.9933
3	ENSG00000163808.2	"kinesin-like 7; kinesin-like protein 2. [Source:RefSeq;Acc:NM_020242]"	opposite	13283	8.65991
3	ENSG00000163833.3	"muscle disease-related protein. [Source:RefSeq;Acc:NM_016298]"	same	12322	1.36744
3			opposite	14036	9.04266
3	ENSG00000168038.2	(blank)	opposite	11565	3.57693
3	ENSG00000168160.4	"Metabotropic glutamate receptor 7 precursor (mGluR7). [Source:SWISSPROT;Acc:Q14831]"	same	11365	5.14045
3	ENSG00000169064.2	(blank)	opposite	12803	6.95158
3				12804	20.7235
3	ENSG00000169760.4	"Neuroligin 1 precursor. [Source:SWISSPROT;Acc:Q8N2Q7]"	opposite	14592	4.37163
3	ENSG00000169855.5	"roundabout 1 isoform a; roundabout 1; axon guidance receptor. [Source:RefSeq;Acc:NM_002941]"	opposite	11697	16.7925
3	ENSG00000170011.2	"Rab effector MyRIP (Myosin-VIIa- and Rab-interacting protein) (Exophilin 8) (Slp homolog lacking C2 domains-c). [Source:SWISSPROT;Acc:Q8NFW9]"	same	11557	2.67807
3	ENSG00000170819.1	"Phakinin (Beaded filament structural protein 2) (Lens fiber cell beaded filament protein CP 49) (CP49) (49 kDa cytoskeletal protein) (CP 47) (CP47) (Lens intermediate filament like-light) (LIFL-L). [Source:SWISSPROT;Acc:Q13515]"	same	12392	16.578
3	ENSG00000171088.1	"Histamine H1 receptor. [Source:SWISSPROT;Acc:P35367]"	opposite	13057	10.0402
3	ENSG00000172139.3	(blank)	opposite	12245	1.44335
3	ENSG00000172578.2	"Kelch-like protein 6. [Source:SWISSPROT;Acc:Q8WZ60]"	opposite	12931	1.2479
3	ENSG00000172667.2	"p53 target zinc finger protein isoform 1; zinc finger protein WIG1; WIG-1/PAG608 protein. [Source:RefSeq;Acc:NM_022470]"	opposite	12905	14.0119
3	ENSG00000172752.3	(blank)	opposite	14068	19.0173
3	ENSG00000173389.4	(blank)	same	13322	10.7558
3			opposite	11608	14.7273
3				11611	6.96952
3	ENSG00000173421.4	(blank)	same	11594	24.4797
3			opposite	13308	15.2837
3	ENSG00000174640.3	"Solute carrier family 21 member 2 (Prostaglandin transporter) (PGT). [Source:SWISSPROT;Acc:Q92959]"	opposite	12397	7.9659
3	ENSG00000174948.3	"G protein-coupled receptor PGR10 (Fragment). [Source:SPTREMBL;Acc:Q86SP6]"	opposite	12575	7.70751
3	ENSG00000175928.1	"leucine rich repeat neuronal 1. [Source:RefSeq;Acc:NM_020873]"	opposite	13017	5.84825
3	ENSG00000176945.3	"mucin 20. [Source:RefSeq;Acc:NM_152673]"	same	12988	1.29043
3	ENSG00000178055.5	"testis serine protease 2. [Source:RefSeq;Acc:NM_182702]"	same	13303	4.97266
3	ENSG00000179152.3	(blank)	opposite	13278	15.7649

Chr	Ensembl id	Description	Strand	HERV id	HERV score
3	ENSG00000179799.4	"Seven transmembrane helix receptor. [Source:SPTREMBL;Acc:Q8NHB5]"	same	13386	38.2321
3	ENSG00000181804.2	solute carrier family 9 (sodium/hydrogen exchanger)	opposite	12440	21.8188
3	ENSG00000182247.1	ubiquitin-conjugating enzyme E2E 2 (UBC4/5 homolog	opposite	13142	2.42396
3	ENSG00000183625.2	"C-C chemokine receptor type 3 (C-C CKR-3) (CC-CKR-3) (CCR-3) (CCR3) (CKR3) (Eosinophil eotaxin receptor). [Source:SWISSPROT;Acc:P51677]"	opposite	13294	16.2431
3	ENSG00000183662.1	"TAFA1. [Source:SPTREMBL;Acc:Q7Z5A9]"	same	11647	7.60009
3	ENSG00000183960.1	"Potassium voltage-gated channel subfamily H member 8 (Ether-a-go-go- like potassium channel 3) (ELK channel 3) (ELK3) (ELK1) (hElk1). [Source:SWISSPROT;Acc:Q96L42]"	opposite	13101	27.3844
3	ENSG00000184500.3	"Vitamin K-dependent protein S precursor. [Source:SWISSPROT;Acc:P07225]"	opposite	11967	20.4756
3	ENSG00000185565.1	"Limbic system-associated membrane protein precursor (LSAMP). [Source:SWISSPROT;Acc:Q13449]"	same	13992	11.7228
4	ENSG00000038210.1	"phosphatidylinositol 4-kinase type-II beta; likely ortholog of mouse phosphatidylinositol 4-kinase type 2 beta. [Source:RefSeq;Acc:NM_018323]"	opposite	17355	23.4402
4	ENSG00000087008.3	Acyl-coenzyme A oxidase 3	opposite	14778	25.2086
4				14779	8.66843
4	ENSG00000109220.1	"cysteine-rich hydrophobic domain 2; BRX-like translocated in leukemia; cystein-rich hydrophobic domain 2. [Source:RefSeq;Acc:NM_012110]"	opposite	15210	10.9777
4	ENSG00000109323.1	"Beta-mannosidase precursor (EC 3.2.1.25) (Mannanase) (Mannase). [Source:SWISSPROT;Acc:O00462]"	same	18394	9.27675
4	ENSG00000109458.1	"GRB2-associated binding protein 1. [Source:RefSeq;Acc:NM_002039]"	opposite	19024	7.40914
4	ENSG00000109472.1	"Carboxypeptidase H precursor (EC 3.4.17.10) (CPH) (Carboxypeptidase E) (CPE) (Enkephalin convertase) (Prohormone processing carboxypeptidase). [Source:SWISSPROT;Acc:P16870]"	opposite	19271	5.79495
4	ENSG00000109586.1	"polypeptide N-acetylgalactosaminyltransferase 7. [Source:RefSeq;Acc:NM_017423]"	opposite	19412	8.168
4	ENSG00000109674.1	"DNA glycosylase hFPG2. [Source:RefSeq;Acc:NM_018248]"	opposite	19503	3.06013
4	ENSG00000109689.3	"Stromal interaction molecule 2 precursor. [Source:SWISSPROT;Acc:Q9P246]"	opposite	17356	3.60513
4				17357	6.06244
4	ENSG00000109697.5	"Lipopolysaccharide-responsive and beige-like anchor protein (CDC4-like protein) (Beige-like protein). [Source:SWISSPROT;Acc:P50851]"	same	19060	4.80571
4				19061	4.68807
4	ENSG00000109762.4	"Sorting nexin 25 (MSTP043). [Source:SWISSPROT;Acc:Q9H3E2]"	opposite	19567	10.3424
4	ENSG00000118777.2	ATP-binding cassette	same	18206	25.2901
4	ENSG00000124406.3	"Potential phospholipid-transporting ATPase IA (EC 3.6.3.1) (Chromaffin granule ATPase II) (ATPase class I type 8A member 1). [Source:SWISSPROT;Acc:Q9Y2Q0]"	opposite	15105	6.73092
4	ENSG00000127589.1	"Tubulin beta-4q chain. [Source:SWISSPROT;Acc:Q99867]"	same	19602	17.7658
4	ENSG00000131127.4	"Zinc finger protein 141. [Source:SWISSPROT;Acc:Q15928]"	same	14747	20.1293
4				14748	4.79246
4				14749	17.6066
4			opposite	17201	6.49515
4	ENSG00000137462.2	"Toll-like receptor 2 precursor (Toll/interleukin 1 receptor-like protein 4). [Source:SWISSPROT;Acc:O60603]"	opposite	19074	20.292
4	ENSG00000138653.1	"N-deacetylase/N-sulfotransferase (heparan glucosaminyl) 4; N-deacetylase/N-sulfotransferase 4. [Source:RefSeq;Acc:NM_022569]"	opposite	16049	4.44547
4				16050	4.99418
4	ENSG00000138759.4	"Fraser syndrome 1 isoform 1; extracellular matrix protein. [Source:RefSeq;Acc:NM_025074]"	same	15697	8.3448
4	ENSG00000145242.3	"Ephrin type-A receptor 5 precursor (EC 2.7.1.112) (Tyrosine-protein kinase receptor EHK-1) (Eph homology kinase-1) (Receptor protein-tyrosine kinase HEK7). [Source:SWISSPROT;Acc:P54756]"	opposite	15421	5.72202
4	ENSG00000145348.3	(blank)	opposite	15993	21.2442
4	ENSG00000145375.2	"spermatogenesis associated factor SPAF. [Source:RefSeq;Acc:NM_145207]"	opposite	18603	1.76079

Chr	Ensembl id	Description	Strand	HERV id	HERV score
4	ENSG00000145431.1	"platelet-derived growth factor C precursor; spinal cord-derived growth factor; secretory growth factor-like protein; fallotein. [Source:RefSeq;Acc:NM_016205]"	opposite	16708	2.31824
4	ENSG00000150471.2	"latrophilin 3; latrophilin homolog 3 (cow); lectomedin 3. [Source:RefSeq;Acc:NM_015236]"	opposite	17816	9.49212
4	ENSG00000151612.3	(blank)	opposite	16630	13.6936
4	ENSG00000151623.4	"Mineralocorticoid receptor (MR). [Source:SWISSPROT;Acc:P08235]"	opposite	16645	7.93464
4	ENSG00000151790.1	Tryptophan 2	opposite	19107	1.94886
4	ENSG00000151834.4	"Gamma-aminobutyric-acid receptor alpha-2 subunit precursor (GABA(A) receptor). [Source:SWISSPROT;Acc:P47869]"	opposite	15177	5.94512
4	ENSG00000152208.2	"Glutamate receptor delta-2 subunit precursor (GluR delta-2). [Source:SWISSPROT;Acc:O43424]"	opposite	18277	13.1198
4				18281	3.06229
4	ENSG00000153064.3	"B-cell scaffold protein with ankyrin repeats 1. [Source:RefSeq;Acc:NM_017935]"	opposite	18390	30.7491
4	ENSG00000154274.4	(blank)	same	15097	8.68739
4	ENSG00000156096.4	UDP-glucuronosyltransferase 2B11 precursor	same	18011	10.6168
4			opposite	15546	9.64399
4				15547	24.3908
4				15557	8.19723
4				15561	9.13235
4				15566	3.57488
4				15571	9.12166
4	ENSG00000156234.2	"Small inducible cytokine B13 precursor (CXCL13) (B lymphocyte chemoattractant) (CXC chemokine BLC) (B cell-attracting chemokine 1) (BCA-1) (ANGIE). [Source:SWISSPROT;Acc:O43927]"	same	15692	1.04826
4	ENSG00000163071.1	(blank)	opposite	17710	3.83743
4	ENSG00000163697.4	"Amyloid beta A4 precursor protein-binding family B member 2 (Fe65-like protein) (Fragment). [Source:SWISSPROT;Acc:Q92870]"	opposite	15101	3.65655
4	ENSG00000164024.2	"Methionine aminopeptidase 1 (EC 3.4.11.18) (MetAP 1) (MAP 1) (Peptidase M 1). [Source:SWISSPROT;Acc:P53582]"	opposite	18346	19.7166
4	ENSG00000164025.5	"Alcohol dehydrogenase class III chi chain (EC 1.1.1.1) (Glutathione- dependent formaldehyde dehydrogenase) (EC 1.2.1.1) (FDH). [Source:SWISSPROT;Acc:P11766]"	same	18347	9.31825
4				18350	12.6898
4				18351	5.72701
4			opposite	15904	6.55389
4				15905	19.3197
4	ENSG00000164124.2	(blank)	opposite	19149	9.13386
4	ENSG00000164162.3	"Anaphase promoting complex subunit 10 (APC10) (Cyclosome subunit 10). [Source:SWISSPROT;Acc:Q9UM13]"	opposite	16628	8.00514
4	ENSG00000164344.1	"Plasma kallikrein precursor (EC 3.4.21.34) (Plasma prekallikrein) (Kininogenin) (Fletcher factor). [Source:SWISSPROT;Acc:P03952]"	opposite	19573	21.3931
4	ENSG00000168843.3	"follistatin-like 5. [Source:RefSeq;Acc:NM_020116]"	opposite	16794	4.66891
4	ENSG00000170088.4	(blank)	opposite	16878	3.4598
4	ENSG00000170516.5	"cytochrome c oxidase subunit VIIb2. [Source:RefSeq;Acc:NM_130902]"	opposite	15183	3.92905
4				15184	6.35967
4				15185	3.65158
4	ENSG00000171234.3	UDP-glucuronosyltransferase 2B7 precursor	same	15557	8.19723
4				15561	9.13235
4				15566	3.57488
4				15571	9.12166
4			opposite	18011	10.6168
4	ENSG00000178177.1	"transcription factor MLR1. [Source:RefSeq;Acc:NM_153686]"	opposite	14835	4.39154
4	ENSG00000178331.2	UDP-glucuronosyltransferase 2B17 precursor	same	17991	9.43303

Chr	Ensembl id	Description	Strand	HERV id	HERV score
4			opposite	15497	26.2999
4				15498	3.84494
4				15499	17.9257
4	ENSG00000180801.4	(blank)	same	18457	5.45113
4	ENSG00000181381.2	(blank)	opposite	16941	4.58403
4	ENSG00000181982.2	(blank)	opposite	14883	4.25652
4	ENSG00000183423.2	(blank)	opposite	18445	15.0448
4	ENSG00000184305.3	(blank)	opposite	18222	18.3163
4				18231	0.719592
4				18232	26.3596
4				18233	18.7181
4				18235	1.03146
4				18241	8.51018
4	ENSG00000186777.2	"Zinc finger protein 27 (Zinc finger protein KOX22) (Fragment). [Source:SWISSPROT;Acc:P17033]"	same	17203	26.1599
4				17204	14.6679
4			opposite	14755	2.34428
4				14756	3.12538
4	ENSG00000186877.1	(blank)	opposite	19407	20.2056
5	ENSG00000035499.1	"HBxAg transactivated protein 1. [Source:RefSeq;Acc:NM_018369]"	opposite	20340	18.2322
5	ENSG00000039123.2	(blank)	opposite	21939	12.6112
5	ENSG00000039560.3	"retinoic acid induced 14; novel retinal pigment epithelial gene. [Source:RefSeq;Acc:NM_015577]"	opposite	21762	18.3227
5	ENSG00000064652.1	"Sorting nexin 24 (SBBI31). [Source:SWISSPROT;Acc:Q9Y343]"	opposite	22589	4.56239
5				22590	5.9026
5	ENSG00000069018.4	"Short transient receptor potential channel 7 (TrpC7) (TRP7 protein). [Source:SWISSPROT;Acc:Q9HCX4]"	opposite	21067	10.9823
5	ENSG00000073578.2	Succinate dehydrogenase [ubiquinone] flavoprotein subunit	same	19605	16.9204
5	ENSG00000081842.5	"Protocadherin alpha 6 precursor (PCDH-alpha6). [Source:SWISSPROT;Acc:Q9UN73]"	opposite	22680	6.60391
5	ENSG00000082068.1	(blank)	opposite	21777	0.764352
5	ENSG00000112977.3	"Death-associated protein. [Source:SPTREMBL;Acc:Q9BUC9]"	opposite	19661	6.94147
5	ENSG00000113159.4	(blank)	opposite	20431	5.48051
5	ENSG00000113303.1	"butyrophilin-like 8. [Source:RefSeq;Acc:NM_024850]"	same	21243	3.01796
5	ENSG00000113360.3	"Ribonuclease III (EC 3.1.26.3) (RNase III) (p241). [Source:SWISSPROT;Acc:Q9NRR4]"	opposite	20126	3.68005
5	ENSG00000113448.4	cAMP-specific 3'	same	21949	4.47884
5			opposite	20338	2.68765
5	ENSG00000113657.2	"Dihydropyrimidinase related protein-3 (DRP-3) (Unc-33-like phosphoprotein) (ULIP protein) (Collapsin response mediator protein 4) (CRMP-4). [Source:SWISSPROT;Acc:Q14195]"	opposite	21098	4.39067
5	ENSG00000122012.3	(blank)	same	20438	4.70602
5	ENSG00000129595.2	"Band 4.1-like protein 4A (NBL4 protein). [Source:SWISSPROT;Acc:Q9HCS5]"	opposite	20907	3.3769
5	ENSG00000145526.2	"Cadherin-18 precursor (Cadherin-14). [Source:SWISSPROT;Acc:Q13634]"	same	21360	5.96088
5			opposite	19761	22.1454
5	ENSG00000145623.1	"oncostatin M receptor. [Source:RefSeq;Acc:NM_003999]"	opposite	21780	2.05569
5	ENSG00000145626.2	(blank)	same	21769	4.59538
5			opposite	20150	4.68582
5	ENSG00000145703.3	"Ras GTPase-activating-like protein IQGAP2. [Source:SWISSPROT;Acc:Q13576]"	same	20440	7.85035
5	ENSG00000145819.2	"Oligophrenin-1 like protein (GTPase regulator associated with focal adhesion kinase). [Source:SWISSPROT;Acc:Q9UNA1]"	opposite	22687	5.66191

Chr	Ensembl id	Description	Strand	HERV id	HERV score
5	ENSG00000145826.1	"Leukocyte cell-derived chemotaxin 2 precursor (hLECT2). [Source:SWISSPROT;Acc:O14960]"	same	22655	5.44476
5	ENSG00000145908.2	"Zinc finger protein 300. [Source:SWISSPROT;Acc:Q96RE9]"	same	22721	1.90176
5	ENSG00000150712.2	"phosphatidylinositol-3 phosphate 3-phosphatase adaptor subunit; 3-phosphatase adapter subunit. [Source:RefSeq;Acc:NM_019061]"	opposite	20127	7.62162
5	ENSG00000154162.2	Brain-cadherin precursor (BR-cadherin) (Cadherin-12) (N-cadherin 2) (Cadherin	opposite	19829	3.81758
5				19831	4.32794
5	ENSG00000156475.4	Serine/threonine protein phosphatase 2A	opposite	21097	11.9549
5	ENSG00000158987.5	"PDZ domain containing guanine nucleotide exchange factor 2 (PDZ-GEF2) (Rap guanine nucleotide exchange factor) (RA-GEF-2). [Source:SWISSPROT;Acc:Q8TEU7]"	opposite	21049	21.5737
5				21050	19.8881
5	ENSG00000164197.2	(blank)	opposite	21982	4.33847
5	ENSG00000168690.3	(blank)	opposite	21766	7.13479
5	ENSG00000169302.3	(blank)	opposite	22702	5.37372
5	ENSG00000169570.1	(blank)	same	22528	12.0932
5	ENSG00000170624.2	"Delta-sarcoglycan (SG-delta) (35 kDa dystrophin-associated glycoprotein) (35DAG). [Source:SWISSPROT;Acc:Q92629]"	opposite	22748	22.1336
5	ENSG00000171643.3	"S-100Z protein. [Source:SWISSPROT;Acc:Q8WXG8]"	same	20442	19.9269
5			opposite	22058	14.9631
5	ENSG00000174705.2	(blank)	opposite	21224	16.022
5	ENSG00000177876.2	"similar to ADAMTS-10 precursor. [Source:RefSeq;Acc:NM_197941]"	opposite	20381	9.40957
5	ENSG00000183613.3	(blank)	same	20442	19.9269
5			opposite	22058	14.9631
6	ENSG00000008083.2	"Jumonji protein. [Source:SWISSPROT;Acc:Q92833]"	same	22911	3.85434
6	ENSG00000055291.5	"CMP-sialic acid transporter (CMP-Sia-Tr) (CMP-SA-Tr) (Solute carrier family 35 member A1). [Source:SWISSPROT;Acc:P78382]"	opposite	24866	6.40089
6	ENSG00000057663.3	"Autophagy protein 5-like (APG5-like) (Apoptosis-specific protein). [Source:SWISSPROT;Acc:Q9H1Y0]"	opposite	23791	13.8413
6	ENSG00000064999.4	"Ankyrin repeat and SAM domain containing protein 1. [Source:SWISSPROT;Acc:Q92625]"	same	23029	7.83894
6	ENSG00000065833.1	"NADP-dependent malic enzyme (EC 1.1.1.40) (NADP-ME) (Malic enzyme 1). [Source:SWISSPROT;Acc:P48163]"	opposite	23511	7.10483
6				23512	5.6475
6	ENSG00000079841.4	"Regulating synaptic membrane exocytosis protein 1 (Rab3-interacting molecule 1) (RIM 1). [Source:SWISSPROT;Acc:Q86UR5]"	opposite	24708	6.79388
6				24709	2.21122
6	ENSG00000082269.3	(blank)	opposite	24688	8.96813
6	ENSG00000082293.2	"Collagen alpha 1(XIX) chain precursor (Collagen alpha 1(Y) chain). [Source:SWISSPROT;Acc:Q14993]"	opposite	24682	5.48232
6	ENSG00000083123.2	2-oxoisovalerate dehydrogenase beta subunit	opposite	24790	2.78744
6	ENSG00000091831.5	"Estrogen receptor (ER) (Estradiol receptor) (ER-alpha). [Source:SWISSPROT;Acc:P03372]"	opposite	25354	6.38114
6	ENSG00000096654.5	"Zinc finger protein 184. [Source:SWISSPROT;Acc:Q99676]"	same	24315	3.16528
6	ENSG00000111880.3	"mRNA capping enzyme (HCE) (HCAP1) [Includes: Polynucleotide 5'- triphosphatase (EC 3.1.3.33) (mRNA 5'-triphosphatase) (TPase); mRNA guanylyltransferase (EC 2.7.7.50) (GTP--RNA guanylyltransferase) (GTase)]. [Source:SWISSPROT;Acc:O60942]"	opposite	23549	25.6829
6				23552	6.13468
6	ENSG00000111981.1	"UL16 binding protein 1; alcan-beta. [Source:RefSeq;Acc:NM_025218]"	opposite	25348	1.66339
6	ENSG00000112038.4	"Mu-type opioid receptor (MOR-1). [Source:SWISSPROT;Acc:P35372]"	opposite	25379	1.89526
6	ENSG00000112175.1	"Bone morphogenetic protein 5 precursor (BMP-5). [Source:SWISSPROT;Acc:P22003]"	opposite	23155	2.21057
6	ENSG00000112232.2	KH domain-containing	opposite	23192	6.63653
6	ENSG00000112304.1	"Thioesterase superfamily member 2 (PNAS-27) (HT012). [Source:SWISSPROT;Acc:Q9NPJ3]"	opposite	24298	12.139
6	ENSG00000112319.5	"Eyes absent homolog 4. [Source:SWISSPROT;Acc:O95677]"	opposite	25192	5.09279
6	ENSG00000112394.3	solute carrier family 16	same	23819	17.2185
6	ENSG00000112499.2	"solute carrier family 22 member 2 isoform a; organic cation transporter 2. [Source:RefSeq;Acc:NM_003058]"	opposite	24110	10.6709

Chr	Ensembl id	Description	Strand	HERV id	HERV score
6	ENSG00000112541.2	cAMP and cAMP-inhibited cGMP 3'	opposite	24158	24.0437
6				24161	7.16288
6	ENSG00000112837.3	"T-box transcription factor TBX18 (T-box protein 18) (Fragment). [Source:SWISSPROT;Acc:O95935]"	same	24844	22.6399
6	ENSG00000118420.3	(blank)	same	24831	6.08716
6	ENSG00000118492.3	(blank)	opposite	25329	17.778
6	ENSG00000124721.4	dynein	same	23037	3.33901
6	ENSG00000124788.3	"Ataxin-1 (Spinocerebellar ataxia type 1 protein). [Source:SWISSPROT;Acc:P54253]"	opposite	22918	22.2462
6	ENSG00000135298.2	"Brain-specific angiogenesis inhibitor 3 precursor. [Source:SWISSPROT;Acc:O60242]"	opposite	24673	5.90736
6	ENSG00000135338.1	(blank)	opposite	23481	4.71476
6	ENSG00000137198.1	"GMP reductase 1 (EC 1.7.1.7) (Guanosine 5'-monophosphate oxidoreductase 1) (Guanosine monophosphate reductase 1). [Source:SWISSPROT;Acc:P36959]"	opposite	24250	11.6209
6	ENSG00000145982.1	"phenylalanine-tRNA synthetase. [Source:RefSeq;Acc:NM_006567]"	opposite	24210	7.84594
6	ENSG00000146085.1	Methylmalonyl-CoA mutase	same	24405	5.64535
6	ENSG00000146350.2	(blank)	opposite	23864	3.44026
6				23866	5.32362
6				23867	3.68897
6	ENSG00000146416.3	"androgen-induced 1; CGI-103 protein; androgen induced protein. [Source:RefSeq;Acc:NM_016108]"	opposite	25300	23.2016
6	ENSG00000164483.4	"sterile alpha motif domain containing 3. [Source:RefSeq;Acc:NM_152552]"	opposite	23931	12.2658
6	ENSG00000164485.3	"interleukin 22-binding protein isoform 1; class II cytokine receptor; interleukin 22-binding protein. [Source:RefSeq;Acc:NM_052962]"	opposite	23960	13.9085
6	ENSG00000164494.1	"candidate tumor suppressor protein. [Source:RefSeq;Acc:NM_020381]"	opposite	23799	8.10699
6	ENSG00000164711.5	lipoprotein	same	25422	6.10274
6	ENSG00000166308.2	"Complement C4 precursor [Contains: C4a anaphylatoxin]. [Source:SWISSPROT;Acc:P01028]"	opposite	24345	15.1219
6	ENSG00000168379.1	Similar to major histocompatibility complex	opposite	24352	4.98349
6	ENSG00000168415.5	HLA class II histocompatibility antigen	same	24347	19.2147
6				24349	22.8568
6			opposite	23022	7.56738
6				23024	8.80626
6				23025	2.40072
6	ENSG00000168745.5	HLA class I histocompatibility antigen	same	23011	4.25765
6				23012	4.64502
6				23013	2.88173
6	ENSG00000170927.3	"Polycystic kidney and hepatic disease 1 precursor (Fibrocystin) (Polyductin) (Tigmin). [Source:SWISSPROT;Acc:Q8TCZ9]"	opposite	23113	11.3327
6	ENSG00000172245.3	"Laminin alpha-2 chain precursor (Laminin M chain) (Merosin heavy chain). [Source:SWISSPROT;Acc:P24043]"	opposite	25185	5.49531
6	ENSG00000172673.1	(blank)	opposite	23919	5.08928
6	ENSG00000181126.3	(blank)	same	23004	3.56256
6	ENSG00000181355.5	"MRDS1; orofacial clefting chromosomal breakpoint region 1. [Source:RefSeq;Acc:NM_153003]"	opposite	22896	7.61071
6	ENSG00000187200.3	HLA class I histocompatibility antigen	same	24340	3.92316
6				24341	12.0823
6	ENSG00000187888.1	HLA class I histocompatibility antigen	same	23006	2.47945
6				23007	4.17422
6				23008	2.74376
6	ENSG00000188107.1	EGF-like-domain	same	24549	3.04196
7	ENSG00000004866.5	"suppression of tumorigenicity 7 isoform a; family with sequence similarity 4"	opposite	27442	3.71664
7	ENSG00000005108.1	(blank)	opposite	25553	9.46663

Chr	Ensembl id	Description	Strand	HERV id	HERV score
7	ENSG00000005249.1	"cAMP-dependent protein kinase type II-beta regulatory chain. [Source:SWISSPROT;Acc:P31323]"	opposite	27345	5.40119
7	ENSG00000006530.4	(blank)	opposite	27624	21.179
7	ENSG00000006576.5	"putative homeodomain transcription factor 2. [Source:RefSeq;Acc:NM_020432]"	opposite	27153	4.35977
7	ENSG00000006747.4	"Adseverin (Scinderin). [Source:SWISSPROT;Acc:Q9Y6U3]"	opposite	26750	23.5158
7	ENSG00000021461.5	"Cytochrome P450 3A43 (EC 1.14.14.1). [Source:SWISSPROT;Acc:Q9HB55]"	opposite	27316	8.6618
7	ENSG00000042813.1	"zona pellucida binding protein. [Source:RefSeq;Acc:NM_007009]"	same	26989	3.89079
7				26991	12.9578
7				26992	8.49626
7	ENSG00000064419.4	"transportin 3; transportin-SR; importin 12. [Source:RefSeq;Acc:NM_012470]"	opposite	26497	3.26962
7	ENSG00000081803.3	"Ca2+-dependent activator protein for secretion 2. [Source:RefSeq;Acc:NM_017954]"	opposite	26444	11.4296
7	ENSG00000091129.4	"Neuronal cell adhesion molecule precursor (Nr-CAM) (NgCAM-related cell adhesion molecule) (Ng-CAM-related) (hBravo). [Source:SWISSPROT;Acc:Q92823]"	opposite	26269	21.1256
7	ENSG00000105778.4	(blank)	same	25700	15.5747
7			opposite	26858	9.76771
7	ENSG00000105866.1	"Transcription factor Sp4 (SPR-1). [Source:SWISSPROT;Acc:Q02446]"	opposite	26818	17.5288
7	ENSG00000105894.2	"Pleiotrophin precursor (PTN) (Heparin-binding growth-associated molecule) (HB-GAM) (Heparin-binding growth factor 8) (HBGF-8) (Osteoblast specific factor 1) (OSF-1) (Heparin-binding neurite outgrowth promoting factor 1) (HBNF-1). [Source:SWISSPROT;Acc:P21"	same	27608	8.8904
7	ENSG00000105983.5	"limb region 1 protein; limb region 1. [Source:RefSeq;Acc:NM_022458]"	opposite	26635	6.47376
7	ENSG00000106078.5	"cordon-bleu homolog. [Source:RefSeq;Acc:NM_015198]"	opposite	25833	3.75062
7	ENSG00000106121.2	(blank)	same	25695	5.76909
7	ENSG00000106580.3	"Aldo-keto reductase family 1 member B10 (EC 1.1.1.-) (Aldose reductase-like) (ARL-1) (Small intestine reductase) (SI reductase) (Aldose reductase-related protein) (ARP) (hARP). [Source:SWISSPROT;Acc:O60218]"	same	26511	22.4133
7				26513	31.5306
7	ENSG00000122779.3	"Transcription intermediary factor 1-alpha (TIF1-alpha) (Tripartite motif protein 24). [Source:SWISSPROT;Acc:O15164]"	opposite	27619	18.8536
7	ENSG00000128510.1	"Carboxypeptidase A4 precursor (EC 3.4.17.-) (Carboxypeptidase A3). [Source:SWISSPROT;Acc:Q9UI42]"	opposite	27563	8.37878
7	ENSG00000128512.4	"Dedicator of cytokinesis protein 4. [Source:SWISSPROT;Acc:Q8N1I0]"	opposite	26316	5.45767
7	ENSG00000128578.1	(blank)	opposite	27560	7.65164
7	ENSG00000130226.5	"Dipeptidyl aminopeptidase-like protein 6 (Dipeptidylpeptidase VI) (Dipeptidylpeptidase 6) (Dipeptidyl peptidase IV like protein) (Dipeptidyl aminopeptidase-related protein) (DPPX). [Source:SWISSPROT;Acc:P42658]"	same	26629	10.2551
7				26630	30.0881
7				26631	22.5835
7	ENSG00000131558.3	"Exocyst complex component Sec8. [Source:SWISSPROT;Acc:Q96A65]"	opposite	27580	3.34927
7	ENSG00000136261.4	"basic leucine zipper and W2 domains 2; HSPC028 protein. [Source:RefSeq;Acc:NM_014038]"	opposite	26797	5.66235
7	ENSG00000136267.3	Diacylglycerol kinase	opposite	25607	4.47859
7				25609	3.55035
7	ENSG00000146856.3	(blank)	opposite	27585	1.68046
7	ENSG00000150851.3	(blank)	same	25685	12.4997
7	ENSG00000152926.2	"Zinc finger protein 117 (Zinc finger protein HPF9) (Fragment). [Source:SWISSPROT;Acc:Q03924]"	same	27110	22.7965
7	ENSG00000154438.1	Germ cell-specific ankyrin	same	27443	2.18552
7	ENSG00000155093.3	"Receptor-type protein-tyrosine phosphatase N2 precursor (EC 3.1.3.48) (R-PTP-N2) (Islet cell autoantigen related protein) (ICAAR) (IAR) (Phogrin). [Source:SWISSPROT;Acc:Q92932]"	opposite	26641	4.64363
7	ENSG00000155849.3	"Engulfment and cell motility protein 1 (CED-12 homolog). [Source:SWISSPROT;Acc:Q92556]"	same	26891	2.55425
7	ENSG00000157680.3	Diacylglycerol kinase	opposite	26526	4.16257
7	ENSG00000157764.2	"B-Raf proto-oncogene serine/threonine-protein kinase (EC 2.7.1.37) (p94) (v-Raf murine sarcoma viral oncogene homolog B1).	opposite	26546	17.0299

Chr	Ensembl id	Description	Strand	HERV id	HERV score
		[Source:SWISSPROT;Acc:P15056]"			
7				26547	9.84384
7	ENSG00000160870.3	"Cytochrome P450 3A7 (EC 1.14.14.1) (CYPIIIA7) (P450-HFLA). [Source:SWISSPROT;Acc:P24462]"	opposite	26234	18.8358
7	ENSG00000161040.3	"F-box and leucine-rich repeat protein 13. [Source:RefSeq;Acc:NM_145032]"	opposite	26241	9.80135
7	ENSG00000164597.3	"Conserved oligomeric Golgi complex subunit 5 (13S Golgi transport complex 90 kDa subunit) (GTC-90) (Golgi transport complex 1). [Source:SWISSPROT;Acc:Q9UP83]"	opposite	26262	9.52303
7				26263	6.92757
7	ENSG00000170381.1	"Semaphorin 3E precursor. [Source:SWISSPROT;Acc:O15041]"	opposite	26095	12.0578
7	ENSG00000171082.4	(blank)	opposite	27633	5.85162
7	ENSG00000171115.1	"immune associated nucleotide 6; immune associated nucleotide. [Source:RefSeq;Acc:NM_175571]"	opposite	27705	4.42642
7	ENSG00000174469.4	"Contactin associated protein-like 2 precursor (Cell recognition molecule Caspr2). [Source:SWISSPROT;Acc:Q9UHC6]"	opposite	27674	11.1748
7				27676	3.48584
7				27694	11.4106
7	ENSG00000175600.3	(blank)	opposite	26908	7.82823
7				26910	6.35069
7	ENSG00000178234.1	"GALNAC-T11. [Source:RefSeq;Acc:NM_022087]"	opposite	27721	15.8902
7				27722	20.3266
7	ENSG00000179603.5	"Metabotropic glutamate receptor 8 precursor (mGluR8). [Source:SWISSPROT;Acc:O00222]"	same	27559	4.98622
7			opposite	26489	5.00248
7				26491	3.08258
7				26493	4.02754
7	ENSG00000181342.4	"Zinc finger protein ZFD25. [Source:SWISSPROT;Acc:Q9UII5]"	same	25944	5.46201
7				25954	4.31886
7				25956	20.0229
7			opposite	27104	9.87238
7				27106	5.07642
7	ENSG00000181444.3	(blank)	same	27697	23.9546
7			opposite	26615	9.16076
7				26616	5.09041
7	ENSG00000182165.3	"TP53 activated protein 1; TP53 target gene 1; H_RG012D21.9. [Source:RefSeq;Acc:NM_007233]"	same	27237	9.01983
7	ENSG00000184903.1	"IMP2 inner mitochondrial membrane protease-like; inner mitochondrial membrane peptidase 2 like. [Source:RefSeq;Acc:NM_032549]"	opposite	26310	2.95194
7				26312	4.96618
7	ENSG00000186088.1	(blank)	opposite	26005	13.1048
7	ENSG00000187416.1	"lipoma HMGIC fusion partner-like 3. [Source:RefSeq;Acc:NM_199000]"	same	26251	7.14168
7	ENSG00000188883.1	(blank)	opposite	26544	10.7007
8	ENSG00000036565.3	"Chromaffin granule amine transporter (Vesicular amine transporter 1) (VAT1) (Solute carrier family 18 member 1). [Source:SWISSPROT;Acc:P54219]"	opposite	27898	7.31088
8	ENSG00000047249.4	"Vacuolar ATP synthase subunit H (EC 3.6.3.14) (V-ATPase H subunit) (Vacuolar proton pump H subunit) (V-ATPase 50/57 kDa subunits) (Vacuolar proton pump subunit SFD) (CGI-11). [Source:SWISSPROT;Acc:Q9UI12]"	opposite	28144	25.7311
8	ENSG00000078674.4	"pericentriolar material 1. [Source:RefSeq;Acc:NM_006197]"	same	27885	2.42408
8	ENSG00000104205.3	"Serine/threonine-protein kinase Sgk3 (EC 2.7.1.37) (Serum/glucocorticoid regulated kinase 3) (Serum/glucocorticoid regulated kinase-like). [Source:SWISSPROT;Acc:Q96BR1]"	opposite	29453	4.09369
8	ENSG00000104324.5	"plasma glutamate carboxypeptidase; aminopeptidase. [Source:RefSeq;Acc:NM_016134]"	opposite	29865	4.26016
8	ENSG00000104341.3	"lysosomal-associated transmembrane protein 4 beta; putative integral membrane transporter. [Source:RefSeq;Acc:NM_018407]"	opposite	29866	14.4396

Chr	Ensembl id	Description	Strand	HERV id	HERV score
8	ENSG00000104369.1	"Junctophilin 1 (Junctophilin type 1) (JP-1). [Source:SWISSPROT;Acc:Q9HDC5]"	opposite	28290	6.3037
8	ENSG00000104375.4	"Serine/threonine protein kinase 3 (EC 2.7.1.37) (STE20-like kinase MST2) (MST-2) (Mammalian STE20-like protein kinase 2) (Serine/threonine protein kinase Krs-1). [Source:SWISSPROT;Acc:Q13188]"	opposite	28588	8.90707
8	ENSG00000104450.3	"sperm associated antigen 1; infertility-related sperm protein; TPR-containing protein involved in spermatogenesis; tetratricopeptide repeat-containing protein. [Source:RefSeq;Acc:NM_003114]"	same	28600	5.35593
8	ENSG00000104490.5	"Neurocalcin delta. [Source:SWISSPROT;Acc:P29554]"	opposite	28601	5.14652
8	ENSG00000104755.3	"ADAM 2 precursor (A disintegrin and metalloproteinase domain 2) (Fertilin beta subunit) (PH-30) (PH30). [Source:SWISSPROT;Acc:Q99965]"	opposite	27981	4.38536
8				27985	1.06869
8	ENSG00000120526.4	"chronic myelogenous leukemia tumor antigen 66. [Source:RefSeq;Acc:NM_032869]"	opposite	28656	10.92
8	ENSG00000120889.1	"Tumor necrosis factor receptor superfamily member 10B precursor (Death receptor 5) (TNF-related apoptosis-inducing ligand receptor 2) (TRAIL receptor-2) (TRAIL-R2). [Source:SWISSPROT;Acc:O14763]"	opposite	27905	3.9208
8	ENSG00000120915.2	"Soluble epoxide hydrolase (SEH) (EC 3.3.2.3) (Epoxide hydratase) (Cytosolic epoxide hydrolase) (CEH). [Source:SWISSPROT;Acc:P34913]"	opposite	29128	26.5239
8	ENSG00000120992.4	"Acyl-protein thioesterase 1 (EC 3.1.2.-) (Lysophospholipase I). [Source:SWISSPROT;Acc:O75608]"	opposite	28149	12.0838
8	ENSG00000132549.3	"Cohen syndrome 1 protein isoform 5. [Source:RefSeq;Acc:NM_017890]"	opposite	29871	8.86195
8	ENSG00000134013.2	"Lysyl oxidase homolog 2 precursor (EC 1.4.3.-) (Lysyl oxidase-like protein 2) (Lysyl oxidase related protein 2) (Lysyl oxidase-related protein WS9-14). [Source:SWISSPROT;Acc:Q9Y4K0]"	same	29111	3.71506
8	ENSG00000134028.3	ADAM-like	opposite	29117	1.93177
8	ENSG00000147481.2	"Gamma-1-syntrophin (G1SYN) (Syntrophin 4) (SYN4). [Source:SWISSPROT;Acc:Q9NSN8]"	same	28082	4.07358
8			opposite	29297	4.49195
8				29302	4.66219
8	ENSG00000147488.3	"suppression of tumorigenicity 18; zinc finger protein 387. [Source:RefSeq;Acc:NM_014682]"	opposite	28116	4.68985
8	ENSG00000147676.3	"MAL2 protein. [Source:SWISSPROT;Acc:Q969L2]"	opposite	30135	2.8561
8	ENSG00000155792.1	"DEP domain containing 6. [Source:RefSeq;Acc:NM_022783]"	opposite	30138	20.3578
8	ENSG00000156006.1	Arylamine N-acetyltransferase 2 (EC 2.3.1.5) (Arylamide acetylase 2) (Arylamine N-acetyltransferase	opposite	29095	8.4984
8	ENSG00000158669.2	"putative lysophosphatidic acid acyltransferase. [Source:RefSeq;Acc:NM_178819]"	opposite	29235	8.1537
8	ENSG00000164793.4	"fibrocystin L; polycystic kidney and hepatic disease-like 1. [Source:RefSeq;Acc:NM_177531]"	opposite	29947	3.78859
8	ENSG00000165078.1	"Carboxypeptidase A6 precursor (EC 3.4.17.1) (Carboxypeptidase B). [Source:SWISSPROT;Acc:Q8N4T0]"	opposite	28245	4.58671
8	ENSG00000168077.3	scavenger receptor class A	opposite	29130	5.19136
8	ENSG00000168615.2	"ADAM 9 precursor (EC 3.4.24.-) (A disintegrin and metalloproteinase domain 9) (Cellular disintegrin-related protein) (Metalloprotease/disintegrin/cysteine-rich protein 9) (Myeloma cell metalloproteinase) (Meltrin gamma). [Source:SWISSPROT;Acc:Q13443]"	opposite	29193	2.99004
8				29194	2.49671
8	ENSG00000168619.5	ADAM 18 precursor (A disintegrin and metalloproteinase domain 18) (Transmembrane metalloproteinase-like	same	27979	4.41349
8				27981	4.38536
8				27985	1.06869
8			opposite	29208	4.0596
8	ENSG00000170631.4	"Zinc finger protein clone 647. [Source:SWISSPROT;Acc:P15622]"	same	30267	12.3908
8				30271	16.7278
8			opposite	28959	12.6403
8	ENSG00000171428.3	Arylamine N-acetyltransferase 1 (EC 2.3.1.5) (Arylamide acetylase 1) (Arylamine N-acetyltransferase	opposite	29086	21.554
8	ENSG00000172817.1	"Cytochrome P450 7B1 (Oxysterol 7-alpha-hydroxylase) (EC 1.14.13.-). [Source:SWISSPROT;Acc:O75881]"	opposite	28233	5.18156
8	ENSG00000176406.5	"Regulating synaptic membrane exocytosis protein 2 (Rab3-interacting molecule 2) (RIM 2). [Source:SWISSPROT;Acc:Q9UQ26]"	opposite	29887	2.32156

Chr	Ensembl id	Description	Strand	HERV id	HERV score
8				29889	2.44209
8	ENSG00000176571.2	(blank)	opposite	29743	14.5811
8				29746	4.78187
8				29750	10.5316
8				29752	3.0665
8	ENSG00000177306.2	"Seven transmembrane helix receptor. [Source:SPTREMBL;Acc:Q8NHA1]"	same	29010	35.1194
8	ENSG00000177335.1	(blank)	same	28952	4.01069
8				28953	6.1786
8	ENSG00000178764.1	"zinc fingers and homeoboxes 2; transcription factor ZHX2. [Source:RefSeq;Acc:NM_014943]"	opposite	30148	24.661
8	ENSG00000182319.1	(blank)	same	29024	5.56838
8	ENSG00000185015.1	"Carbonic anhydrase XIII (EC 4.2.1.1) (Carbonate dehydratase XIII) (CA- XIII). [Source:SWISSPROT;Acc:Q8N1Q1]"	opposite	29731	5.9315
8	ENSG00000185053.1	"Zeta-sarcoglycan (Zeta-SG) (ZSG1). [Source:SWISSPROT;Acc:Q96LD1]"	opposite	27838	2.55127
8	ENSG00000185942.1	(blank)	opposite	29415	16.5472
9	ENSG00000023318.3	"Thioredoxin domain containing protein 4 precursor (Endoplasmic reticulum protein ERp44). [Source:SWISSPROT;Acc:Q9BS26]"	opposite	30839	10.9767
9	ENSG00000029402.2	(blank)	opposite	31503	4.71994
9	ENSG00000078725.1	"deleted in bladder cancer 1; deleted in bladder cancer chromosome region candidate 1. [Source:RefSeq;Acc:NM_014618]"	opposite	30958	3.71194
9	ENSG00000090054.3	"Serine palmitoyltransferase 1 (EC 2.3.1.50) (Long chain base biosynthesis protein 1) (LCB 1) (Serine-palmitoyl-CoA transferase 1) (SPT 1) (SPT1). [Source:SWISSPROT;Acc:O15269]"	opposite	30794	24.0947
9	ENSG00000096872.3	"coiled-coil domain containing 2; capillary morphogenesis protein 1. [Source:RefSeq;Acc:NM_025103]"	opposite	31203	12.4117
9	ENSG00000099810.4	"5'-methylthioadenosine phosphorylase (EC 2.4.2.28) (MTA phosphorylase) (MTAPase). [Source:SWISSPROT;Acc:Q13126]"	same	30421	6.20013
9	ENSG00000107036.1	"BA207C16.2 (Fragment). [Source:SPTREMBL;Acc:Q9NQL7]"	opposite	31034	4.53068
9	ENSG00000107263.5	"Guanine nucleotide-releasing factor 2 (C3G protein) (CRK SH3-binding GNRP). [Source:SWISSPROT;Acc:Q13905]"	opposite	30979	20.8902
9	ENSG00000119471.3	(blank)	opposite	31598	13.8196
9	ENSG00000119509.2	"inversin isoform a; inversion of embryonic turning; nephronophthisis 2. [Source:RefSeq;Acc:NM_014425]"	opposite	31513	15.3555
9	ENSG00000130957.1	Fructose-1	opposite	30813	2.4033
9	ENSG00000136842.3	"Tropomodulin 1 (Erythrocyte tropomodulin) (E-Tmod). [Source:SWISSPROT;Acc:P28289]"	opposite	31504	6.11799
9	ENSG00000136928.2	Gamma-aminobutyric acid type B receptor	opposite	30830	3.24338
9				30831	3.27712
9	ENSG00000148120.1	(blank)	opposite	31496	15.5735
9				31498	2.50336
9	ENSG00000148308.2	general transcription factor IIIC	opposite	31651	21.4834
9	ENSG00000148408.1	Voltage-dependent N-type calcium channel alpha-1B subunit (Calcium channel	opposite	31668	3.61866
9				31669	7.40323
9	ENSG00000155158.4	(blank)	opposite	30408	8.60934
9	ENSG00000160563.3	cofactor required for Sp1 transcriptional activation	opposite	30981	3.36965
9	ENSG00000164989.3	(blank)	opposite	31107	3.85394
9	ENSG00000165115.2	"kinesin family member 27. [Source:RefSeq;Acc:NM_017576]"	opposite	30748	4.58924
9	ENSG00000171097.2	"cytoplasmic cysteine conjugate-beta lyase; glutamine-phenylpyruvate aminotransferase. [Source:RefSeq;Acc:NM_004059]"	opposite	30974	21.3478
9	ENSG00000173077.3	"deleted in esophageal cancer 1. [Source:RefSeq;Acc:NM_017418]"	same	30943	8.35489
9			opposite	31604	24.4728
9	ENSG00000187210.1	Beta-1	opposite	31418	14.8465
9	ENSG00000188312.1	(blank)	opposite	31480	3.9475
10	ENSG00000023839.1	"Canalicular multispecific organic anion transporter 1 (Multidrug resistance-associated protein 2) (Canalicular multidrug resistance protein). [Source:SWISSPROT;Acc:Q92887]"	same	32298	0.960252

Chr	Ensembl id	Description	Strand	HERV id	HERV score
10			opposite	33127	24.6843
10	ENSG00000065618.4	"Collagen alpha 1(XVII) chain (Bullous pemphigoid antigen 2) (180 kDa bullous pemphigoid antigen 2). [Source:SWISSPROT;Acc:Q9UMD9]"	opposite	32309	8.23689
10	ENSG00000066468.5	"Fibroblast growth factor receptor 2 precursor (EC 2.7.1.112) (FGFR-2) (Keratinocyte growth factor receptor 2). [Source:SWISSPROT;Acc:P21802]"	opposite	32411	13.8358
10	ENSG00000068383.4	Type I inositol-1	opposite	33248	8.24736
10	ENSG00000107518.3	(blank)	opposite	33187	2.00623
10				33188	4.83372
10	ENSG00000107611.2	"cubilin; intrinsic factor-cobalamin receptor; intrinsic factor B12-receptor. [Source:RefSeq;Acc:NM_001081]"	same	32545	18.5305
10	ENSG00000107779.1	"Bone morphogenetic protein receptor type IA precursor (EC 2.7.1.37) (Serine/threonine-protein kinase receptor R5) (SKR5) (Activin receptor-like kinase 3) (ALK-3). [Source:SWISSPROT;Acc:P36894]"	same	32234	6.29159
10	ENSG00000107954.1	"neuralized-like. [Source:RefSeq;Acc:NM_004210]"	opposite	33134	9.31079
10	ENSG00000107987.5	"Aldo-keto reductase family 1 member C4 (EC 1.1.1.-) (Chlordecone reductase) (EC 1.1.1.225) (CDR) (3-alpha-hydroxysteroid dehydrogenase) (EC 1.1.1.50) (3-alpha-HSD) (Dihydrodiol dehydrogenase 4) (DD4) (HAKRA). [Source:SWISSPROT;Acc:P17516]"	same	31699	2.41415
10				31701	9.70021
10			opposite	32471	3.90054
10	ENSG00000108100.5	"cyclin fold protein 1; cyclin-box carrying protein 1. [Source:RefSeq;Acc:NM_145012]"	opposite	32641	13.446
10	ENSG00000120292.5	"Zinc finger protein 11B (Fragment). [Source:SWISSPROT;Acc:Q06732]"	same	32676	11.873
10				32677	10.6994
10			opposite	31855	21.06
10				31856	19.9458
10	ENSG00000120594.4	"plexin domain containing 2 precursor; tumor endothelial marker 7-related; 1200007L24Rik. [Source:RefSeq;Acc:NM_032812]"	opposite	32576	12.9278
10	ENSG00000121898.2	"Potential carboxypeptidase-like protein X2 precursor. [Source:SWISSPROT;Acc:Q8N436]"	opposite	32421	6.10657
10	ENSG00000126542.2	(blank)	same	32696	6.97704
10	ENSG00000132334.4	"Protein-tyrosine phosphatase epsilon precursor (EC 3.1.3.48) (R-PTP- epsilon). [Source:SWISSPROT;Acc:P23469]"	same	32425	5.40739
10				32426	5.88365
10	ENSG00000133661.2	"Pulmonary surfactant-associated protein D precursor (SP-D) (PSP-D). [Source:SWISSPROT;Acc:P35247]"	same	32990	13.815
10			opposite	32151	11.4166
10	ENSG00000134461.2	(blank)	opposite	31704	16.5621
10	ENSG00000138185.3	"Ectonucleoside triphosphate diphosphohydrolase 1 (EC 3.6.1.5) (NTPDase1) (Ecto-ATP diphosphohydrolase) (ATPDase) (Lymphoid cell activation antigen) (Ecto-apyrase) (CD39 antigen). [Source:SWISSPROT;Acc:P49961]"	opposite	33121	17.2568
10	ENSG00000148429.1	(blank)	opposite	31730	4.27624
10	ENSG00000148680.4	"5-hydroxytryptamine 7 receptor (5-HT-7) (5-HT-X) (Serotonin receptor) (5HT7). [Source:SWISSPROT;Acc:P34969]"	opposite	32271	5.00751
10				32272	13.897
10	ENSG00000150076.5	"coiled-coil domain containing 7. [Source:RefSeq;Acc:NM_145023]"	same	31816	5.64572
10				31817	4.67611
10				31818	2.69895
10			opposite	32633	6.42689
10	ENSG00000150275.3	"Protocadherin 15 precursor. [Source:SWISSPROT;Acc:Q96QU1]"	same	32787	4.5554
10			opposite	31939	9.95612
10				31949	8.28006
10	ENSG00000152487.1	"nucleolar protein (NOL1/NOP2/sun) and PUA domains 1. [Source:RefSeq;Acc:NM_182543]"	opposite	31754	7.14109
10	ENSG00000165404.5	"zinc finger protein 485; Zinc finger protein 93 (Zinc finger protein HTF34). [Source:RefSeq;Acc:NM_145312]"	same	31859	44.2671
10			opposite	32682	5.58281

Chr	Ensembl id	Description	Strand	HERV id	HERV score
10				32683	20.6369
10				32684	27.0561
10	ENSG00000165449.2	solute carrier family 16 (monocarboxylic acid transporters)	same	32887	9.66393
10	ENSG00000165841.1	"Cytochrome P450 2C19 (EC 1.14.13.80) ((R)-limonene 6-monooxygenase) (CYPIIC19) (P450-11A) (Mephenytoin 4-hydroxylase) (CYPIIC17) (P450-254C). [Source:SWISSPROT;Acc:P33261]"	opposite	33107	20.8475
10				33108	20.1338
10	ENSG00000165886.1	(blank)	opposite	33123	8.29952
10				33124	5.76276
10	ENSG00000165983.3	"Phosphotriesterase related protein (Parathion hydrolase-related protein) (HPHRP). [Source:SWISSPROT;Acc:Q96BW5]"	opposite	32543	6.15227
10	ENSG00000169740.4	"Zinc finger protein 239 (Zinc finger protein MOK-2) (HOK-2). [Source:SWISSPROT;Acc:Q16600]"	same	32683	20.6369
10				32684	27.0561
10			opposite	31859	44.2671
10	ENSG00000172987.2	"heparanase 2; heparanase-like protein; heparanase 3. [Source:RefSeq;Acc:NM_021828]"	opposite	32295	3.51126
10	ENSG00000173876.2	tubulin	same	32441	4.63891
10	ENSG00000175395.4	"Zinc finger protein 25 (Zinc finger protein KOX19). [Source:SWISSPROT;Acc:P17030]"	opposite	31831	24.2077
10	ENSG00000179133.1	(blank)	opposite	31781	4.74005
10	ENSG00000182771.2	"Glutamate receptor delta-1 subunit precursor (GluR delta-1). [Source:SWISSPROT;Acc:Q9ULK0]"	opposite	32231	2.81266
10				32233	4.46631
10	ENSG00000183230.1	catenin (cadherin-associated protein)	opposite	32109	8.99706
10				32114	13.1523
10	ENSG00000183675.3	(blank)	same	32708	6.95004
10	ENSG00000185532.2	cGMP-dependent protein kinase 1	opposite	32737	4.21107
10				32739	22.2062
10	ENSG00000185736.3	"Double-stranded RNA-specific editase B2 (EC 3.5.-.-) (dsRNA adenosine deaminase B2) (RNA dependent adenosine deaminase 3) (RNA editing deaminase 2) (RNA editing enzyme 2). [Source:SWISSPROT;Acc:Q9NS39]"	opposite	31672	10.192
10	ENSG00000185904.2	(blank)	same	31855	21.06
10	ENSG00000189180.1	"Zinc finger protein 33A (Zinc finger protein KOX31) (HA0946). [Source:SWISSPROT;Acc:Q06730]"	same	31831	24.2077
11	ENSG00000048028.4	"Ubiquitin carboxyl-terminal hydrolase 28 (EC 3.1.2.15) (Ubiquitin thiolesterase 28) (Ubiquitin-specific processing protease 28) (Deubiquitinating enzyme 28). [Source:SWISSPROT;Acc:Q96RU2]"	opposite	34163	1.53988
11	ENSG00000086991.2	"NADPH oxidase 4. [Source:RefSeq;Acc:NM_016931]"	opposite	33925	18.5733
11	ENSG00000087884.4	"PTD015 protein. [Source:RefSeq;Acc:NM_024684]"	opposite	34772	28.4604
11	ENSG00000109911.4	"elongation protein 4 homolog; PAX6 neighbor gene; chromosome 11 open reading frame 19. [Source:RefSeq;Acc:NM_019040]"	opposite	34495	13.878
11	ENSG00000129084.4	"Proteasome subunit alpha type 1 (EC 3.4.25.1) (Proteasome component C2) (Macropain subunit C2) (Multicatalytic endopeptidase complex subunit C2) (Proteasome nu chain) (30 kDa prosomal protein) (PROS-30). [Source:SWISSPROT;Acc:P25786]"	opposite	33341	23.5037
11				33342	4.83138
11	ENSG00000129744.1	"GPI-linked NAD(P)(+)--arginine ADP-ribosyltransferase 1 precursor (EC 2.4.2.31) (Mono(ADP-ribosyl)transferase). [Source:SWISSPROT;Acc:P52961]"	same	33281	15.0425
11	ENSG00000133812.4	"SET binding factor 2. [Source:RefSeq;Acc:NM_030962]"	opposite	33332	4.49481
11	ENSG00000134343.3	"Transmembrane protein 16C. [Source:SWISSPROT;Acc:Q9BYT9]"	opposite	34458	12.5019
11	ENSG00000137494.2	(blank)	opposite	34801	6.30454
11	ENSG00000137634.1	(blank)	opposite	34169	22.0964
11	ENSG00000137737.1	"layilin. [Source:RefSeq;Acc:NM_178834]"	opposite	35068	4.27377
11	ENSG00000149084.2	"steroid dehydrogenase homolog; 3-ketoacyl-CoA reductase. [Source:RefSeq;Acc:NM_016142]"	opposite	34603	7.5444
11	ENSG00000149089.1	(blank)	opposite	33484	22.0911

Chr	Ensembl id	Description	Strand	HERV id	HERV score
11	ENSG00000149295.4	"D(2) dopamine receptor. [Source:SWISSPROT;Acc:P14416]"	opposite	34161	5.4661
11	ENSG00000149308.3	nuclear protein	opposite	34143	26.7794
11	ENSG00000149452.5	"solute carrier family 22 member 8; organic anion transporter 3. [Source:RefSeq;Acc:NM_004254]"	opposite	33760	11.1324
11				33761	15.4318
11	ENSG00000149548.3	(blank)	opposite	35118	6.44698
11	ENSG00000150672.3	Channel associated protein of synapse-110 (Chapsyn-110) (Discs	same	34806	4.17056
11	ENSG00000151376.4	NADP-dependent malic enzyme	opposite	33898	3.94205
11	ENSG00000152404.3	CWF19-like 2	opposite	34140	3.55711
11	ENSG00000152578.1	Glutamate receptor 4 precursor (GluR-4) (GluR4) (GluR-D) (Glutamate receptor ionotropic	opposite	35036	4.06382
11	ENSG00000162105.2	"SH3 and multiple ankyrin repeat domains protein 2 (Shank2). [Source:SWISSPROT;Acc:Q9UPX8]"	opposite	33789	5.53692
11	ENSG00000162174.2	"asparaginase-like 1 protein. [Source:RefSeq;Acc:NM_025080]"	opposite	34719	12.8036
11				34720	21.7598
11	ENSG00000165325.3	(blank)	opposite	34865	3.58518
11	ENSG00000165973.5	"Protein kinase C-binding protein NELL1 precursor (NEL-like protein 1) (Nel-related protein 1). [Source:SWISSPROT;Acc:Q92832]"	same	33366	2.42808
11	ENSG00000166323.2	(blank)	same	35056	3.89208
11			opposite	34146	5.00816
11	ENSG00000166435.4	"X-ray radiation resistance associated 1. [Source:SPTREMBL;Acc:Q7Z463]"	opposite	33806	16.124
11				33807	5.62899
11	ENSG00000166693.2	"Olfactory receptor 5M1 (OST050). [Source:SWISSPROT;Acc:Q8NGP8]"	opposite	33677	3.10311
11	ENSG00000166961.3	(blank)	same	33733	5.17923
11				33734	8.4182
11				33735	8.82897
11	ENSG00000168959.3	"Metabotropic glutamate receptor 5 precursor (mGluR5). [Source:SWISSPROT;Acc:P41594]"	opposite	33911	2.628
11				33918	7.01314
11	ENSG00000169519.4	(blank)	same	33453	8.69522
11	ENSG00000170959.2	(blank)	same	34493	4.11598
11	ENSG00000171994.3	"Olfactory receptor 52K1. [Source:SWISSPROT;Acc:Q8NGK4]"	same	33287	3.20159
11			opposite	34282	16.9919
11				34283	6.12067
11	ENSG00000172324.2	"Olfactory receptor 5A2. [Source:SWISSPROT;Acc:Q8NGI9]"	same	34696	20.0399
11				34697	6.39808
11				34698	14.2965
11			opposite	33699	15.0199
11				33708	17.6505
11				33712	6.75616
11	ENSG00000181995.2	(blank)	same	33732	4.14929
11	ENSG00000182634.3	"Olfactory receptor 10G7. [Source:SWISSPROT;Acc:Q8NGN6]"	same	35113	7.95989
11	ENSG00000182667.2	"Neurotrimin precursor (hNT). [Source:SWISSPROT;Acc:Q9P121]"	opposite	35155	7.98844
11	ENSG00000183715.1	"Opioid binding protein/cell adhesion molecule precursor (OBCAM) (Opioid-binding cell adhesion molecule) (OPCML). [Source:SWISSPROT;Acc:Q14982]"	opposite	34244	3.22968
11				34245	5.20504
11	ENSG00000185688.2	"Olfactory receptor 8B8 (Olfactory receptor TPCR85) (Olfactory-like receptor JCG8). [Source:SWISSPROT;Acc:Q15620]"	same	35111	13.1667
11				35113	7.95989
11	ENSG00000187398.1	(blank)	opposite	34420	22.4848

Chr	Ensembl id	Description	Strand	HERV id	HERV score
11	ENSG00000188211.1	(blank)	same	33351	22.8378
12	ENSG00000004700.5	"ATP-dependent DNA helicase Q1 (DNA-dependent ATPase Q1). [Source:SWISSPROT;Acc:P46063]"	opposite	35339	4.03071
12	ENSG00000006831.3	"Adiponectin receptor protein 2. [Source:SWISSPROT;Acc:Q86V24]"	opposite	36141	29.7795
12	ENSG00000018236.4	"Contactin precursor (Glycoprotein gP135). [Source:SWISSPROT;Acc:Q12860]"	opposite	36505	8.29168
12	ENSG00000047617.3	"Transmembrane protein 16B. [Source:SWISSPROT;Acc:Q9NQ90]"	opposite	35173	7.30776
12				35174	4.45163
12	ENSG00000074527.2	"netrin 4; beta-netrin. [Source:RefSeq;Acc:NM_021229]"	opposite	36042	11.7568
12	ENSG00000079081.5	"Similar to SLIT-ROBO Rho GTPase-activating protein 1. [Source:SPTREMBL;Acc:Q8N6J5]"	opposite	36708	5.70945
12	ENSG00000083782.1	"Dermatan sulfate proteoglycan 3 precursor (Epiphycan) (Small chondroitin/dermatan sulfate proteoglycan) (Proteoglycan-Lb) (PG-Lb). [Source:SWISSPROT;Acc:Q99645]"	opposite	36017	8.80748
12	ENSG00000086117.3	"Sodium channel protein type VIII alpha subunit. [Source:SWISSPROT;Acc:Q9UQD0]"	opposite	36570	20.1438
12				36571	7.35361
12	ENSG00000089818.4	(blank)	same	35207	4.20236
12				35208	3.75301
12	ENSG00000090612.5	"Zinc finger protein 26 (Zinc finger protein KOX20) (Fragment). [Source:SWISSPROT;Acc:P17031]"	same	36135	14.6867
12			opposite	37203	16.2758
12	ENSG00000091039.4	"Oxysterol binding protein-related protein 8 (OSBP-related protein 8) (ORP-8). [Source:SWISSPROT;Acc:Q9BZF1]"	opposite	35792	4.6691
12	ENSG00000111110.2	(blank)	opposite	35644	4.86496
12	ENSG00000111218.1	"Protein arginine N-methyltransferase 4 (EC 2.1.1.-). [Source:SWISSPROT;Acc:Q9NR22]"	same	35165	5.91549
12				35166	6.23242
12	ENSG00000111249.2	"Homeobox protein Cux-2 (Cut-like 2) (Fragment). [Source:SWISSPROT;Acc:O14529]"	opposite	37161	5.14743
12				37162	3.9793
12	ENSG00000111700.2	"Solute carrier family 21 member 8 (Organic anion transporter 8) (Organic anion transporting polypeptide 8) (OATP8) (Liver-specific organic anion transporter 2) (LST-2). [Source:SWISSPROT;Acc:Q9NPD5]"	opposite	36295	13.2133
12				36296	9.31559
12	ENSG00000111713.1	Glycogen [starch] synthase	opposite	35340	7.14234
12	ENSG00000118307.3	"cancer susceptibility candidate 1; lung adenoma susceptibility 1-like. [Source:RefSeq;Acc:NM_018272]"	opposite	35373	10.093
12	ENSG00000120853.3	(blank)	same	37119	11.3441
12	ENSG00000122970.4	"carnitine deficiency-associated gene expressed in ventricle 1. [Source:RefSeq;Acc:NM_014055]"	opposite	37160	3.60857
12	ENSG00000123104.2	Inositol 1	opposite	35387	6.37246
12	ENSG00000127325.4	"Bestrophin 4 (Vitelliform macular dystrophy 2-like protein 3). [Source:SWISSPROT;Acc:Q8N1M1]"	same	36740	5.3078
12	ENSG00000127337.1	"glioma-amplified sequence-41; NuMA binding protein 1. [Source:RefSeq;Acc:NM_006530]"	opposite	36739	7.18612
12	ENSG00000127720.1	(blank)	opposite	36927	8.02398
12	ENSG00000129317.3	(blank)	opposite	35495	6.28212
12	ENSG00000134283.4	"periphilin 1 isoform 5; gastric cancer antigen Ga50. [Source:RefSeq;Acc:NM_201438]"	same	35487	16.7198
12	ENSG00000135426.2	(blank)	same	36581	13.1776
12	ENSG00000136052.1	solute carrier family 41	opposite	36074	4.9095
12	ENSG00000139144.1	"Phosphatidylinositol-4-phosphate 3-kinase C2 domain-containing gamma polypeptide (EC 2.7.1.154) (Phosphoinositide 3-Kinase-C2-gamma) (PtdIns-3-kinase C2 gamma) (PI3K-C2gamma). [Source:SWISSPROT;Acc:O75747]"	opposite	36276	5.24137
12				36278	4.93603
12	ENSG00000139146.3	(blank)	same	36441	1.66891
12	ENSG00000139173.1	(blank)	same	35502	2.27749
12			opposite	36528	20.9795
12	ENSG00000139292.2	"Leucine-rich repeat-containing G protein-coupled receptor 5 precursor (Orphan G protein-coupled receptor HG38) (G protein-coupled	opposite	36760	6.96297

Chr	Ensembl id	Description	Strand	HERV id	HERV score
		receptor 49). [Source:SWISSPROT;Acc:O75473]"			
12	ENSG00000139324.3	(blank)	opposite	37038	18.3492
12	ENSG00000139430.1	"T-cell activation protein phosphatase 2C. [Source:RefSeq;Acc:NM_139283]"	opposite	36093	11.6202
12	ENSG00000150045.2	killer cell lectin-like receptor subfamily F	opposite	36180	4.04298
12	ENSG00000151490.4	"Receptor-type protein-tyrosine phosphatase O precursor (EC 3.1.3.48) (Glomerular epithelial protein 1) (Protein tyrosine phosphatase U2) (PTPase U2) (PTP-U2). [Source:SWISSPROT;Acc:Q16827]"	opposite	36241	2.71625
12	ENSG00000151746.2	"Cytoskeleton-like bicaudal D protein homolog 1. [Source:SWISSPROT;Acc:Q96G01]"	same	35428	3.50359
12	ENSG00000151952.4	(blank)	opposite	36126	7.18707
12	ENSG00000152936.3	(blank)	same	36374	2.44921
12	ENSG00000166532.3	(blank)	opposite	36166	18.3652
12	ENSG00000171435.3	"kinase suppressor of Ras-2. [Source:RefSeq;Acc:NM_173598]"	opposite	36103	5.23819
12	ENSG00000171681.4	"activating transcription factor 7 interacting protein; MBD1-containing chromatin associated factor. [Source:RefSeq;Acc:NM_018179]"	opposite	36218	6.03402
12	ENSG00000173588.3	"NY-REN-58 antigen. [Source:RefSeq;Acc:NM_016122]"	opposite	36028	17.1588
12				36029	1.22267
12	ENSG00000177478.4	"Seven transmembrane helix receptor. [Source:SPTREMBL;Acc:Q8NH09]"	same	35534	5.03474
12			opposite	36562	2.72561
12	ENSG00000179520.1	solute carrier family 17 (sodium-dependent inorganic phosphate cotransporter)	opposite	37120	2.56273
12	ENSG00000183880.4	"zinc finger protein 605. [Source:RefSeq;Acc:NM_183238]"	same	37203	16.2758
12	ENSG00000185046.3	"E2a-Pbx1-associated protein; amyloid-beta precursor protein intracellular domain associated protein 1; cajalin 2. [Source:RefSeq;Acc:NM_020140]"	opposite	36054	17.171
12	ENSG00000185306.1	(blank)	same	36709	6.66845
12			opposite	35650	3.6119
12	ENSG00000186908.3	"huntingtin interacting protein 14; Huntingtin interacting protein H; huntingtin interacting protein 3; zinc finger"	same	35796	5.66966
13	ENSG00000032742.4	"Recessive polycystic kidney disease protein Tg737 homolog. [Source:SWISSPROT;Acc:Q13099]"	opposite	38270	19.4898
13	ENSG00000080618.2	"plasma carboxypeptidase B2 isoform a preproprotein; carboxypeptidase U; thrombin-activatable fibrinolysis inhibitor; carboxypeptidase B-like protein; thrombin-activable fibrinolysis inhibitor. [Source:RefSeq;Acc:NM_001872]"	opposite	37339	4.82875
13	ENSG00000083544.1	"Tudor domain containing protein 3. [Source:SWISSPROT;Acc:Q9H7E2]"	same	37497	3.09948
13				37498	6.9597
13	ENSG00000102452.3	"voltage gated channel like 1; four repeat voltage-gated ion channel. [Source:RefSeq;Acc:NM_052867]"	opposite	38210	19.7982
13	ENSG00000102531.3	(blank)	opposite	38418	5.60879
13	ENSG00000102606.4	"Rho guanine nucleotide exchange factor 7 (PAK-interacting exchange factor beta) (Beta-Pix) (COOL-1) (p85). [Source:SWISSPROT;Acc:Q14155]"	opposite	39379	22.3105
13	ENSG00000102683.1	"Gamma-sarcoglycan (Gamma-SG) (35 kDa dystrophin-associated glycoprotein) (35DAG). [Source:SWISSPROT;Acc:Q13326]"	same	37239	5.86743
13	ENSG00000120662.3	Peptide chain release factor 1	same	38393	6.74149
13	ENSG00000120675.1	"DNAJ domain-containing; methylation-controlled J protein. [Source:RefSeq;Acc:NM_013238]"	opposite	38398	7.18447
13	ENSG00000120697.1	"Dolichyl-phosphate beta-glucosyltransferase (EC 2.4.1.117) (DolP- glucosyltransferase) (HSPC149). [Source:SWISSPROT;Acc:Q9Y673]"	opposite	37299	13.7368
13	ENSG00000127870.3	"RING finger protein 6. [Source:SWISSPROT;Acc:Q9Y252]"	same	38288	4.38962
13	ENSG00000132938.5	(blank)	opposite	38300	4.48987
13	ENSG00000133104.2	"spartin; trans-activated by hepatitis C virus core protein 1. [Source:RefSeq;Acc:NM_015087]"	opposite	37296	14.7876
13	ENSG00000134900.2	"Tripeptidyl-peptidase II (EC 3.4.14.10) (TPP-II) (Tripeptidyl aminopeptidase). [Source:SWISSPROT;Acc:P29144]"	opposite	39319	15.9283
13	ENSG00000136160.5	"Endothelin B receptor precursor (ET-B) (Endothelin receptor Non-selective type). [Source:SWISSPROT;Acc:P24530]"	same	38904	16.1373
13				38905	6.80734
13				38906	9.97052

Chr	Ensembl id	Description	Strand	HERV id	HERV score
13	ENSG00000165487.3	"Smhs2 homolog. [Source:RefSeq;Acc:NM_152726]"	opposite	37221	6.71522
13	ENSG00000172915.4	"Neurobeachin protein (Lysosomal trafficking regulator 2) (BCL8B protein). [Source:SWISSPROT;Acc:Q8NFP9]"	opposite	38329	12.4334
13	ENSG00000175198.2	Propionyl-CoA carboxylase alpha chain	same	38209	31.1435
13			opposite	39306	7.48408
13	ENSG00000177527.1	(blank)	opposite	37623	12.305
13				37624	18.4932
13	ENSG00000184226.3	"Protocadherin 9 precursor. [Source:SWISSPROT;Acc:Q9HC56]"	opposite	37687	5.56504
13	ENSG00000184497.1	(blank)	opposite	38264	10.7606
13	ENSG00000187676.1	"beta 3-glycosyltransferase-like. [Source:RefSeq;Acc:NM_194318]"	opposite	38302	1.17687
14	ENSG00000071537.2	"Sel-1 homolog precursor (Suppressor of lin-12-like protein) (Sel-1L). [Source:SWISSPROT;Acc:Q9UBV2]"	opposite	39950	3.9862
14	ENSG00000080823.5	"MAPK/MAK/MRK overlapping kinase (EC 2.7.1.37) (MOK protein kinase) (Renal tumor antigen 1) (RAGE-1). [Source:SWISSPROT;Acc:Q9UQ07]"	opposite	40108	19.1132
14	ENSG00000100436.1	(blank)	opposite	40403	22.4721
14	ENSG00000100523.3	"Probable phospholipase DDHD1 (EC 3.1.1.-) (DDHD domain protein 1) (Phosphatidic acid-preferring phospholipase A1 homolog) (PA-PLA1). [Source:SWISSPROT;Acc:Q8NEL9]"	opposite	39846	15.7662
14	ENSG00000100592.3	"Disheveled associated activator of morphogenesis 1. [Source:SWISSPROT;Acc:Q9Y4D1]"	opposite	40504	6.88689
14	ENSG00000100599.4	"Ras and Rab interactor 3 (Ras interaction/interference protein 3). [Source:SWISSPROT;Acc:Q8TB24]"	opposite	40701	4.43707
14	ENSG00000100784.2	"Ribosomal protein S6 kinase alpha 5 (EC 2.7.1.37) (Nuclear mitogen-and stress-activated protein kinase-1) (90 kDa ribosomal protein S6 kinase 5) (RSK-like protein kinase) (RLSK). [Source:SWISSPROT;Acc:O75582]"	opposite	40057	6.09945
14				40058	3.33999
14	ENSG00000119686.1	(blank)	opposite	40603	18.8401
14	ENSG00000130076.5	"Ig alpha-1 chain C region. [Source:SWISSPROT;Acc:P01876]"	same	40750	3.24711
14				40760	23.4871
14			opposite	40124	2.59541
14	ENSG00000131969.4	(blank)	same	39829	6.74262
14	ENSG00000133958.2	"KIAA1409. [Source:RefSeq;Acc:NM_020818]"	same	40071	4.29013
14			opposite	40710	17.3893
14	ENSG00000133983.1	(blank)	opposite	39920	24.5508
14	ENSG00000139915.4	"MAM domain containing 1. [Source:RefSeq;Acc:NM_182830]"	opposite	39776	11.9284
14	ENSG00000139971.3	"Human full-length cDNA clone CS0DC025YL05 of neuroblastoma of Homo sapiens (Human). [Source:SPTREMBL;Acc:Q86TY3]"	opposite	39860	59.5417
14	ENSG00000139985.3	"ADAM 21 precursor (EC 3.4.24.-) (A disintegrin and metalloproteinase domain 21). [Source:SWISSPROT;Acc:Q9UKJ8]"	same	39920	24.5508
14			opposite	40585	18.5662
14				40587	4.90705
14				40588	22.0239
14	ENSG00000140015.5	"Potassium voltage-gated channel subfamily H member 5 (Ether-a-go-go potassium channel 2) (hEAG2). [Source:SWISSPROT;Acc:Q8NCM2]"	same	40530	14.4866
14				40532	8.10694
14	ENSG00000151338.5	"mirror-image polydactyly 1; mirror-image polydactyly gene 1. [Source:RefSeq;Acc:NM_138731]"	opposite	40248	2.43163
14	ENSG00000151413.5	(blank)	opposite	40230	15.4496
14	ENSG00000171723.5	"Gephyrin. [Source:SWISSPROT;Acc:Q9NQX3]"	opposite	40551	6.82775
14	ENSG00000182732.3	"Regulator of G-protein signaling 6 (RGS6) (S914). [Source:SWISSPROT;Acc:P49758]"	opposite	40598	6.35107
14	ENSG00000185024.2	Transcription factor IIIB 90 kDa subunit (TFIIIB90) (hTFIIIB90) (B- related factor 1) (hBRF) (TATA box-binding protein-associated factor	same	40741	1.34183
14	ENSG00000188899.1	"Ig heavy chain V-III region DOB. [Source:SWISSPROT;Acc:P01782]"	same	40750	3.24711
14			opposite	40124	2.59541

Chr	Ensembl id	Description	Strand	HERV id	HERV score
14	ENSG00000189012.1	"Striatin 3 (Cell-cycle autoantigen SG2NA) (S/G2 antigen). [Source:SWISSPROT;Acc:Q13033]"	opposite	39527	5.16029
15	ENSG00000092529.5	"Calpain 3 (EC 3.4.22.-) (Calpain L3) (Calpain p94) (Calcium-activated neutral proteinase 3) (CANP 3) (Muscle-specific calcium-activated neutral protease 3) (nCL-1). [Source:SWISSPROT;Acc:P20807]"	same	40903	1.59583
15	ENSG00000103733.1	"Adapter-related protein complex 3 beta 2 subunit (Beta-adaptin 3B) (AP-3 complex beta-3B subunit) (Beta-3B-adaptin) (Neuron-specific vesicle coat protein beta-NAP). [Source:SWISSPROT;Acc:Q13367]"	opposite	41040	12.6341
15	ENSG00000104044.4	"P protein (Melanocyte-specific transporter protein). [Source:SWISSPROT;Acc:Q04671]"	opposite	40847	5.19246
15	ENSG00000128739.5	"Small nuclear ribonucleoprotein associated protein N (snRNP-N) (Sm protein N) (Sm-N) (SmN) (Sm-D) (Tissue-specific splicing protein). [Source:SWISSPROT;Acc:P14648]"	opposite	41245	1.22278
15				41247	4.04451
15	ENSG00000128881.5	"tau tubulin kinase 2; tau-tubulin kinase. [Source:RefSeq;Acc:NM_173500]"	opposite	40904	17.2859
15	ENSG00000134146.1	(blank)	opposite	40880	7.65686
15	ENSG00000134152.2	(blank)	same	41283	12.6616
15	ENSG00000137819.2	"membrane progestin receptor gamma; progestin and adipoQ receptor family member V. [Source:RefSeq;Acc:NM_017705]"	same	40994	6.04033
15	ENSG00000137869.2	"Cytochrome P450 19A1 (Aromatase) (EC 1.14.14.1) (CYPXIX) (Estrogen synthetase) (P-450AROM). [Source:SWISSPROT;Acc:P11511]"	opposite	40946	14.486
15	ENSG00000140374.4	Electron transfer flavoprotein alpha-subunit	opposite	41015	20.9454
15	ENSG00000140386.3	"Zinc finger protein 291. [Source:SWISSPROT;Acc:Q9BY12]"	opposite	41018	6.31601
15	ENSG00000140459.1	Cytochrome P450 11A1	opposite	41004	10.3721
15	ENSG00000140470.2	"ADAMTS-17 precursor (EC 3.4.24.-) (A disintegrin and metalloproteinase with thrombospondin motifs 17) (ADAM-TS 17) (ADAM-TS17). [Source:SWISSPROT;Acc:Q8TE56]"	opposite	41116	4.61201
15	ENSG00000140543.3	"de-etiolated 1. [Source:RefSeq;Acc:NM_017996]"	opposite	41071	21.3575
15	ENSG00000154227.3	"LAG1 longevity assurance homolog 3. [Source:RefSeq;Acc:NM_178842]"	opposite	41119	15.7494
15	ENSG00000167014.2	(blank)	same	40911	10.658
15				40912	12.9224
15	ENSG00000175921.4	(blank)	opposite	41038	20.1479
15	ENSG00000183083.4	(blank)	opposite	41487	20.9184
15	ENSG00000186322.3	(blank)	opposite	41049	21.3049
15	ENSG00000186702.3	(blank)	opposite	41053	21.1476
16	ENSG00000077754.4	"Leucine carboxyl methyltransferase 1 (EC 2.1.1.-) (Protein-leucine O- methyltransferase) (CGI-68). [Source:SWISSPROT;Acc:Q9UIC8]"	opposite	41904	14.3691
16	ENSG00000102879.3	"Coronin-like protein p57 (Coronin 1A). [Source:SWISSPROT;Acc:P31146]"	same	41619	3.68897
16	ENSG00000103196.1	(blank)	opposite	42138	6.91351
16	ENSG00000129636.2	"T-cell immunomodulatory protein. [Source:RefSeq;Acc:NM_030790]"	opposite	41696	0.739214
16	ENSG00000140650.2	"Phosphomannomutase 2 (EC 5.4.2.8) (PMM 2). [Source:SWISSPROT;Acc:O15305]"	opposite	41855	14.8225
16	ENSG00000141037.3	"Tubulin beta-4 chain (Tubulin beta-III). [Source:SWISSPROT;Acc:Q13509]"	same	41840	5.55224
16	ENSG00000152910.4	"Contactin associated protein-like 4 precursor (Cell recognition molecule Caspr4). [Source:SWISSPROT;Acc:Q9C0A0]"	opposite	42116	4.58585
16	ENSG00000155718.4	(blank)	opposite	41897	19.9941
16	ENSG00000161980.2	"DNA-directed RNA polymerases III 12.5 kDa polypeptide (EC 2.7.7.6) (RNA polymerase III C11 subunit) (HsC11p) (hRPC11) (My010 protein). [Source:SWISSPROT;Acc:Q9Y2Y1]"	opposite	41559	7.27511
16	ENSG00000166501.2	Protein kinase C	same	41598	6.50333
16	ENSG00000166669.4	"activating transcription factor 7 interacting protein 2. [Source:RefSeq;Acc:NM_024997]"	opposite	41863	4.53923
16	ENSG00000166743.2	"acetyl-Coenzyme A synthetase 3; medium-chain acyl-CoA synthetase; Butyrate CoA ligase. [Source:RefSeq;Acc:NM_052956]"	opposite	41587	3.7821
16				41588	4.97073
16				41591	13.2549
16	ENSG00000167411.5	"Zinc finger protein 267 (Zinc finger protein HZF2). [Source:SWISSPROT;Acc:Q14586]"	opposite	41921	19.29

Chr	Ensembl id	Description	Strand	HERV id	HERV score
16	ENSG00000170537.3	"transmembrane channel-like 7. [Source:RefSeq;Acc:NM_024847]"	opposite	41880	5.78048
16				41881	11.6965
16	ENSG00000185984.3	"LAT1-3TM protein. [Source:RefSeq;Acc:NM_031211]"	opposite	41615	3.42924
17	ENSG00000005271.3	"Neurofibromin (Neurofibromatosis-related protein NF-1) [Contains: Neurofibromin truncated]. [Source:SWISSPROT;Acc:P21359]"	same	42216	4.95979
17	ENSG00000007171.4	Nitric oxide synthase	same	42445	19.0274
17			opposite	42205	9.43979
17				42206	15.2117
17				42207	18.8403
17	ENSG00000074755.2	zinc finger	same	42376	8.03147
17	ENSG00000125447.5	ADP-ribosylation factor binding protein GGA3 (Golgi-localized	opposite	42364	10.8174
17	ENSG00000129657.2	"SEC14-like protein 1. [Source:SWISSPROT;Acc:Q92503]"	opposite	42603	7.74055
17	ENSG00000132142.5	"Acetyl-CoA carboxylase 1 (EC 6.4.1.2) (ACC-alpha) [Includes: Biotin carboxylase (EC 6.3.4.14)]. [Source:SWISSPROT;Acc:Q13085]"	opposite	42251	30.5029
17	ENSG00000133195.1	solute carrier family 39 (metal ion transporter)	opposite	42355	3.21131
17	ENSG00000141198.4	"TOM1-like 1 protein (Target of myb-like 1 protein) (Src activating and signaling molecule protein). [Source:SWISSPROT;Acc:O75674]"	opposite	42560	2.98401
17	ENSG00000161583.4	"TBC1 domain family member 3 (Rab GTPase-activating protein PRC17) (Prostate cancer gene 17 protein) (TRE17 alpha protein). [Source:SWISSPROT;Acc:Q8IZP1]"	opposite	42249	6.98371
17				42250	7.42877
17	ENSG00000166260.1	Cytochrome c oxidase assembly protein COX11	same	42560	2.98401
17	ENSG00000166263.2	"syntaxin binding protein 4. [Source:RefSeq;Acc:NM_178509]"	opposite	42563	17.054
17	ENSG00000167080.1	UDP-GalNAc:Neu5Acalpha2-3Galbeta-R beta1	opposite	42502	2.34744
17	ENSG00000175711.2	(blank)	opposite	42371	4.17354
17	ENSG00000176658.4	"Myosin Id. [Source:SWISSPROT;Acc:O94832]"	opposite	42225	4.5055
17	ENSG00000176927.2	(blank)	same	42213	4.77594
17				42214	3.12674
17			opposite	42458	20.3021
17	ENSG00000178743.2	(blank)	same	42492	4.10566
17	ENSG00000182205.1	(blank)	opposite	42370	12.7882
17	ENSG00000182352.1	(blank)	same	42359	8.04227
18	ENSG00000053747.3	"Laminin alpha-3 chain precursor (Epiligrin 170 kDa subunit) (E170) (Nicein alpha subunit). [Source:SWISSPROT;Acc:Q16787]"	opposite	43369	4.70302
18	ENSG00000067900.1	Rho-associated	same	43365	6.28499
18	ENSG00000078043.3	"protein inhibitor of activated STAT X isoform alpha. [Source:RefSeq;Acc:NM_173206]"	opposite	42990	17.964
18	ENSG00000132204.4	(blank)	opposite	42614	5.33716
18	ENSG00000134759.2	"elongator protein 2; signal transducer and activator of transcription interacting protein 1. [Source:RefSeq;Acc:NM_018255]"	opposite	43499	1.69598
18	ENSG00000134769.4	"Dystrobrevin alpha (Dystrobrevin-alpha). [Source:SWISSPROT;Acc:Q9Y4J8]"	opposite	43493	6.69582
18	ENSG00000141384.1	"Transcription initiation factor TFIID 105 kDa subunit (TAFII-105) (TAFII105) (Fragment). [Source:SWISSPROT;Acc:Q92750]"	same	42710	3.07916
18			opposite	43386	23.8059
18	ENSG00000141404.2	Guanine nucleotide-binding protein G(olf)	opposite	43339	4.045
18	ENSG00000150477.1	(blank)	opposite	43502	8.08561
18	ENSG00000152214.1	"GTP-binding protein Rit2 (Ras-like protein expressed in neurons) (Ras-like without CAAX protein 2). [Source:SWISSPROT;Acc:Q99578]"	opposite	42942	15.6531
18	ENSG00000152234.1	ATP synthase alpha chain	same	43600	0.907923
18	ENSG00000166401.2	"Plasminogen activator inhibitor-2 precursor (PAI-2) (Placental plasminogen activator inhibitor) (Monocyte Arg-serpin) (Urokinase inhibitor). [Source:SWISSPROT;Acc:P05120]"	opposite	43705	6.95798
18	ENSG00000166960.4	(blank)	opposite	42828	7.85805
18	ENSG00000167216.4	(blank)	opposite	43602	6.47439

Chr	Ensembl id	Description	Strand	HERV id	HERV score
18	ENSG00000171791.5	"Apoptosis regulator Bcl-2. [Source:SWISSPROT;Acc:P10415]"	same	43693	3.81945
18	ENSG00000177150.2	(blank)	same	43343	3.81154
18	ENSG00000183287.2	(blank)	opposite	43058	2.77878
18	ENSG00000187323.1	"Tumor suppressor protein DCC precursor (Colorectal cancer suppressor). [Source:SWISSPROT;Acc:P43146]"	opposite	43620	6.91324
18				43621	7.16478
19	ENSG00000011485.2	"Serine/threonine protein phosphatase 5 (EC 3.1.3.16) (PP5) (Protein phosphatase T) (PP-T) (PPT). [Source:SWISSPROT;Acc:P53041]"	same	44176	3.04274
19	ENSG00000081665.2	"Zinc finger protein 93 (Zinc finger protein HTF34) (Fragment). [Source:SWISSPROT;Acc:P35789]"	same	43981	4.28313
19	ENSG00000083844.1	"Zinc finger protein 264. [Source:SWISSPROT;Acc:O43296]"	same	44229	3.87975
19			opposite	44590	7.62708
19	ENSG00000089335.5	"Zinc finger protein 302 (ZNF135-like) (ZNF140-like). [Source:SWISSPROT;Acc:Q9NR11]"	same	44134	9.21229
19	ENSG00000104804.1	"Tubby related protein 2 (Tubby-like protein 2). [Source:SWISSPROT;Acc:O00295]"	opposite	44180	0.772488
19	ENSG00000104823.1	Delta3	opposite	44142	13.8371
19	ENSG00000105708.3	"Zinc finger protein 14 (Zinc finger protein KOX6) (Gonadotropin inducible transcription repressor-4) (GIOT-4). [Source:SWISSPROT;Acc:P17017]"	same	44292	7.2751
19				44297	2.18992
19			opposite	43979	4.69775
19				43980	4.10481
19				43981	4.28313
19				43982	20.3657
19				43983	4.6997
19	ENSG00000105750.3	"Zinc finger protein 85 (Zinc finger protein HPF4) (HTF1). [Source:SWISSPROT;Acc:Q03923]"	same	44009	4.33461
19	ENSG00000118613.4	"Zinc finger protein 43 (Zinc protein HTF6) (Zinc finger protein KOX27). [Source:SWISSPROT;Acc:P17038]"	same	44358	17.8516
19			opposite	44028	19.2179
19				44030	5.70205
19	ENSG00000120777.4	"zinc finger protein 565. [Source:RefSeq;Acc:NM_152477]"	same	44451	16.168
19				44454	19.0334
19			opposite	44138	5.81728
19	ENSG00000120782.4	"Zinc finger protein 268 (Zinc finger protein HZF3). [Source:SWISSPROT;Acc:Q14587]"	same	44465	17.9989
19			opposite	44139	0.974575
19	ENSG00000127903.5	"Zinc finger imprinted 2. [Source:SWISSPROT;Acc:Q9NZV7]"	opposite	44219	3.21057
19				44223	9.14407
19	ENSG00000130607.4	"Cytochrome P450 2B6 (EC 1.14.14.1) (CYPIIB6) (P450 IIB1). [Source:SWISSPROT;Acc:P20813]"	same	44150	11.1901
19			opposite	44474	5.33944
19	ENSG00000153896.4	(blank)	same	44441	15.5296
19				44443	6.89948
19			opposite	44134	9.21229
19	ENSG00000153902.2	"Leucine-rich repeat LGI family member 4 precursor (Leucine-rich glioma-inactivated protein 4) (LGI1-like protein 3) (UNQ6515/PRO21485). [Source:SWISSPROT;Acc:Q8N135]"	same	44446	1.62204
19	ENSG00000160321.3	"Zinc finger protein 257 (Bone marrow zinc finger 4) (BMZF-4). [Source:SWISSPROT;Acc:Q9Y2Q1]"	same	44306	10.1881
19				44307	2.4557
19				44311	1.62587
19				44312	16.8309
19				44313	18.3195
19			opposite	44000	1.63676

Chr	Ensembl id	Description	Strand	HERV id	HERV score
19				44001	1.34151
19				44002	6.21861
19	ENSG00000160336.4	"Similar to zinc finger protein 268 (Fragment). [Source:SPTREMBL;Acc:Q96IR2]"	same	44207	22.8665
19				44208	14.0258
19			opposite	44567	1.67014
19				44568	5.59592
19				44570	19.357
19				44571	15.5196
19	ENSG00000161241.4	"F-box only protein 26. [Source:SWISSPROT;Acc:Q96EF6]"	same	44467	19.3027
19	ENSG00000161640.2	"Sialic acid binding Ig-like lectin 11 precursor (Siglec-11) (Sialic acid-binding lectin 11) (UNQ9222/PRO28718). [Source:SWISSPROT;Acc:Q96RL6]"	opposite	44183	4.88562
19	ENSG00000166704.3	(blank)	opposite	44234	16.6277
19	ENSG00000167232.4	"Zinc finger protein 91 (Zinc finger protein HTF10) (HPF7). [Source:SWISSPROT;Acc:Q05481]"	same	44394	4.60459
19				44395	5.15455
19				44396	14.5704
19				44401	2.87694
19			opposite	44083	14.8783
19				44084	7.39788
19				44086	19.3404
19				44088	19.4834
19				44089	3.62957
19	ENSG00000167584.4	"Cytochrome P450 2A6 (EC 1.14.14.1) (CYPIIA6) (Coumarin 7-hydroxylase) (IIA3) (CYP2A3) (P450(I)). [Source:SWISSPROT;Acc:P11509]"	same	44473	0.956594
19				44474	5.33944
19			opposite	44149	0.965386
19				44150	11.1901
19	ENSG00000167637.5	"Zinc finger protein 345 (Zinc finger protein HZF10). [Source:SWISSPROT;Acc:Q14585]"	opposite	44463	21.2112
19	ENSG00000167766.4	"Zinc finger protein 432. [Source:SWISSPROT;Acc:O94892]"	same	44556	22.3998
19				44557	16.4288
19				44559	15.8594
19				44560	12.8129
19				44561	22.0824
19				44562	18.8392
19			opposite	44194	13.8978
19				44195	7.86036
19				44196	7.22816
19				44197	5.17791
19	ENSG00000171032.1	"zinc finger protein 350; zinc-finger protein ZBRK1. [Source:RefSeq;Acc:NM_021632]"	opposite	44188	8.16138
19	ENSG00000171124.1	"Galactoside 3(4)-L-fucosyltransferase (EC 2.4.1.65) (Blood group Lewis alpha-4-fucosyltransferase) (Lewis FT) (Fucosyltransferase 3) (FUCT- III). [Source:SWISSPROT;Acc:P21217]"	opposite	43922	6.85232
19	ENSG00000171295.2	"Zinc finger protein 440. [Source:SWISSPROT;Acc:Q8IYI8]"	same	43958	4.10025
19			opposite	44276	13.4425
19				44277	17.7986
19	ENSG00000171817.4	"zinc finger protein 540. [Source:RefSeq;Acc:NM_152606]"	opposite	44465	17.9989

Chr	Ensembl id	Description	Strand	HERV id	HERV score
19	ENSG00000172687.3	"Zinc finger protein 431. [Source:SWISSPROT;Acc:Q8TF32]"	same	44014	10.8908
19				44015	21.198
19				44016	10.8448
19			opposite	44336	17.274
19				44342	20.7367
19				44345	13.3304
19				44346	10.7842
19	ENSG00000173875.2	"Zinc finger protein 136. [Source:SWISSPROT;Acc:P52737]"	same	43962	25.0493
19			opposite	44279	18.9661
19				44280	8.36646
19	ENSG00000174652.5	"Zinc finger protein 266 (Zinc finger protein HZF1) (Fragment). [Source:SWISSPROT;Acc:Q14584]"	same	44257	23.7984
19				44258	4.93282
19				44260	8.86384
19				44261	17.6809
19				44264	51.056
19				44265	17.558
19				44266	21.0332
19			opposite	43944	3.40306
19				43946	16.7445
19				43947	7.38769
19				43948	23.0357
19				43949	5.41783
19				43951	22.466
19				43952	7.75645
19				43954	7.78534
19				43955	26.5737
19				43956	7.1718
19	ENSG00000176024.4	"zinc finger protein 613. [Source:RefSeq;Acc:NM_024840]"	same	44188	8.16138
19			opposite	44555	3.66598
19				44556	22.3998
19				44557	16.4288
19				44559	15.8594
19				44560	12.8129
19	ENSG00000176293.5	"Zinc finger protein 135. [Source:SWISSPROT;Acc:P52742]"	same	44234	16.6277
19			opposite	44595	4.8468
19	ENSG00000176379.5	"Zinc finger protein ZFP-36 (Fragment). [Source:SWISSPROT;Acc:P16415]"	same	44276	13.4425
19				44277	17.7986
19				44278	6.84685
19			opposite	43958	4.10025
19				43959	19.5812
19	ENSG00000179847.2	"Similar to zinc finger protein 137 (Clone pHZ-30). [Source:SPTREMBL;Acc:Q9BVV0]"	same	44207	22.8665
19				44208	14.0258
19			opposite	44567	1.67014
19				44568	5.59592

Chr	Ensembl id	Description	Strand	HERV id	HERV score
19	ENSG00000180081.4	"Similar to zinc finger protein 254 (Fragment). [Source:SPTREMBL;Acc:Q86Y87]"	same	44080	17.4921
19				44081	17.8708
19				44083	14.8783
19				44084	7.39788
19				44086	19.3404
19				44088	19.4834
19				44089	3.62957
19				44090	11.4519
19				44092	6.46375
19				44093	5.7488
19			opposite	44388	11.938
19				44389	8.11275
19				44390	13.9793
19				44391	7.58913
19				44394	4.60459
19				44395	5.15455
19				44396	14.5704
19				44401	2.87694
19				44404	17.7179
19				44406	17.423
19				44407	9.63777
19	ENSG00000180855.5	"Zinc finger protein 442. [Source:SWISSPROT;Acc:Q9H7R0]"	same	44280	8.36646
19			opposite	43962	25.0493
19	ENSG00000181515.1	(blank)	opposite	43979	4.69775
19	ENSG00000182797.1	(blank)	opposite	44485	6.74025
19	ENSG00000183668.3	"Pregnancy-specific beta-1-glycoprotein 9 precursor (PSBG-9) (Pregnancy-specific glycoprotein 9) (Pregnancy-specific beta-1 glycoprotein B) (PS-beta-B) (PS34) (Pregnancy-specific glycoprotein 7) (PSG7). [Source:SWISSPROT;Acc:Q00887]"	same	44515	3.29563
19	ENSG00000184635.3	"Zinc finger protein 208. [Source:SWISSPROT;Acc:O43345]"	same	44373	4.53969
19				44374	6.72006
19				44375	9.56732
19				44381	19.5562
19			opposite	44045	6.83316
19				44054	4.19621
19				44055	8.06392
19				44057	17.9076
19				44058	16.9326
19				44063	15.7701
19				44066	3.79144
19				44069	6.28429
19				44070	8.13274
19	ENSG00000188114.1	"Pregnancy-specific beta-1-glycoprotein 5 precursor (PSBG-5) (Fetal liver non-specific cross-reactive antigen-3) (FL-NCA-3). [Source:SWISSPROT;Acc:Q15238]"	same	44510	5.95232
19	ENSG00000188629.1	"Zinc finger protein 177. [Source:SWISSPROT;Acc:Q13360]"	opposite	44258	4.93282
19	ENSG00000189329.1	"Pregnancy-specific beta-1-glycoprotein 2 precursor (PSBG-2) (Pregnancy-specific beta-1 glycoprotein E) (PS-beta-E).	same	44505	6.44603

Chr	Ensembl id	Description	Strand	HERV id	HERV score
		[Source:SWISSPROT;Acc:P11465]"			
20	ENSG00000101080.5	"Zinc finger protein 335. [Source:SWISSPROT;Acc:Q9H4Z2]"	same	44856	6.3276
20	ENSG00000101280.1	"Angiopoietin-4 precursor (ANG-4) (ANG-3). [Source:SWISSPROT;Acc:Q9Y264]"	opposite	44603	6.89256
20	ENSG00000101292.1	"Prokineticin receptor 2 (PK-R2) (G protein-coupled receptor 73-like 1) (GPR73b) (GPRg2). [Source:SWISSPROT;Acc:Q8NFJ6]"	opposite	44610	21.0559
20	ENSG00000101310.3	"Protein transport protein Sec23B (SEC23-related protein B). [Source:SWISSPROT;Acc:Q15437]"	opposite	44794	19.0194
20	ENSG00000124177.5	"Chromodomain-helicase-DNA-binding protein 5 (CHD-5). [Source:SWISSPROT;Acc:Q8TD26]"	opposite	44696	5.85452
20	ENSG00000177410.1	(blank)	opposite	44860	5.56973
20	ENSG00000182621.4	1-phosphatidylinositol-4	same	44622	5.04029
21	ENSG00000155258.5	(blank)	same	44889	13.9354
21	ENSG00000159082.4	Synaptojanin 1 (EC 3.1.3.36) (Synaptic inositol-1	opposite	45112	22.4513
21	ENSG00000159113.1	"Interleukin-10 receptor beta chain precursor (IL-10R-B) (IL-10R2) (Cytokine receptor class-II CRF2-4). [Source:SWISSPROT;Acc:Q08334]"	opposite	45420	4.05077
21	ENSG00000174496.3	"C21ORF34 (Fragment). [Source:SPTREMBL;Acc:Q8TDA7]"	opposite	45167	11.9833
21	ENSG00000182240.3	"Beta secretase 2 precursor (EC 3.4.23.45) (Beta-site APP-cleaving enzyme 2) (Aspartyl protease 1) (Asp 1) (ASP1) (Membrane-associated aspartic protease 1) (Memapsin-1) (Down region aspartic protease). [Source:SWISSPROT;Acc:Q9Y5Z0]"	opposite	45440	2.3051
21				45441	7.26775
21	ENSG00000183570.1	"Poly(rC)-binding protein 3 (Alpha-CP3). [Source:SWISSPROT;Acc:P57721]"	opposite	45457	1.26542
21	ENSG00000188992.1	"membrane-associated phospholipase A1 beta. [Source:RefSeq;Acc:NM_198996]"	opposite	44895	20.5765
22	ENSG00000077942.5	"Fibulin-1 precursor. [Source:SWISSPROT;Acc:P23142]"	opposite	45647	5.78333
22	ENSG00000100055.5	"Cytohesin 4. [Source:SWISSPROT;Acc:Q9UIA0]"	opposite	45638	3.74213
22	ENSG00000100208.5	"Ig lambda chain C regions. [Source:SWISSPROT;Acc:P01842]"	same	45492	5.86162
22				45493	6.82621
22			opposite	45601	3.24553
22				45606	5.24321
22	ENSG00000176177.4	(blank)	opposite	45538	3.0208
22	ENSG00000184470.4	Thioredoxin reductase 2	opposite	45479	10.4055
22	ENSG00000185686.4	"Melanoma antigen preferentially expressed in tumors (Preferentially expressed antigen of melanoma) (OPA-interacting protein 4) (OIP4). [Source:SWISSPROT;Acc:P78395]"	same	45595	3.79978
22				45599	4.46853
22				45600	7.85685
22				45601	3.24553
22			opposite	45492	5.86162
X	ENSG00000008056.4	"Synapsin I (Brain protein 4.1). [Source:SWISSPROT;Acc:P17600]"	opposite	407	4.17495
X	ENSG00000072315.1	"Short transient receptor potential channel 5 (TrpC5) (Htrp-5) (Htrp5). [Source:SWISSPROT;Acc:Q9UL62]"	same	3178	4.58449
X	ENSG00000080561.1	"Midline 2 protein (Midline defect 2) (Tripartite motif protein 1) (Midin 2). [Source:SWISSPROT;Acc:Q9UJV3]"	opposite	3145	24.2944
X	ENSG00000083750.2	"Ras-related GTP binding B short isoform; GTP-binding protein ragB. [Source:RefSeq;Acc:NM_006064]"	opposite	2315	4.67583
X	ENSG00000089472.3	"hephaestin isoform a. [Source:RefSeq;Acc:NM_138737]"	opposite	2431	10.2696
X	ENSG00000101844.5	"autophagy-related cysteine endopeptidase 2 isoform a; autophagy-related cysteine endopeptidase 2; AUT-like 2"	opposite	3149	3.21757
X				3150	5.42306
X	ENSG00000101871.2	"Midline 1 protein (Tripartite motif protein 18) (Putative transcription factor XPRF). [Source:SWISSPROT;Acc:O15344]"	same	1926	8.82493
X	ENSG00000102207.5	(blank)	same	2298	5.24227
X	ENSG00000102290.5	"protocadherin 11 X-linked isoform b precursor; protocadherin X; protocadherin-S. [Source:RefSeq;Acc:NM_032967]"	opposite	2913	3.04468
X				2914	13.1611
X				2916	11.9347

Chr	Ensembl id	Description	Strand	HERV id	HERV score
X	ENSG00000102383.4	"Zinc finger DHHC domain containing protein 15. [Source:SWISSPROT;Acc:Q96MV8]"	same	2516	3.01119
X			opposite	637	3.61227
X				638	6.14862
X	ENSG00000120498.4	"testis expressed sequence 11. [Source:RefSeq;Acc:NM_031276]"	opposite	594	1.08609
X	ENSG00000126733.5	"dachshund 2. [Source:RefSeq;Acc:NM_053281]"	same	841	7.13371
X			opposite	2713	7.66591
X				2715	6.04782
X				2718	3.77357
X	ENSG00000132438.5	"Dystrophin. [Source:SWISSPROT;Acc:P11532]"	opposite	283	10.2764
X	ENSG00000133124.5	"insulin receptor substrate 4. [Source:RefSeq;Acc:NM_003604]"	same	3153	6.89557
X			opposite	1265	8.30485
X	ENSG00000146950.2	"Apical-like protein (APXL protein). [Source:SWISSPROT;Acc:Q13796]"	opposite	1925	6.05979
X	ENSG00000147044.5	"Peripheral plasma membrane protein CASK (EC 2.7.1.-) (hCASK) (Calcium/calmodulin-dependent serine protein kinase) (Lin-2 homolog). [Source:SWISSPROT;Acc:O14936]"	opposite	375	7.51318
X				376	2.0577
X	ENSG00000147050.3	"Ubiquitously transcribed X chromosome tetratricopeptide repeat protein (Ubiquitously transcribed TPR protein on the X chromosome). [Source:SWISSPROT;Acc:O15550]"	opposite	2202	6.02879
X	ENSG00000147082.4	"cyclin B3 isoform 1. [Source:RefSeq;Acc:NM_033670]"	opposite	2248	28.3141
X	ENSG00000147117.3	"Zinc finger protein 157 (HZF22). [Source:SWISSPROT;Acc:P51786]"	same	407	4.17495
X			opposite	2225	9.07868
X				2231	6.06989
X				2232	1.86465
X				2233	5.24723
X	ENSG00000147246.1	"5-hydroxytryptamine 2C receptor (5-HT-2C) (Serotonin receptor) (5HT-1C). [Source:SWISSPROT;Acc:P28335]"	opposite	3203	12.4367
X	ENSG00000156920.2	"G-protein coupled receptor 112. [Source:RefSeq;Acc:NM_153834]"	opposite	3431	3.43992
X	ENSG00000157502.5	(blank)	opposite	3123	8.62633
X	ENSG00000157562.5	"Collagen alpha 5(IV) chain precursor. [Source:SWISSPROT;Acc:P29400]"	same	1265	8.30485
X			opposite	3153	6.89557
X	ENSG00000157625.3	"TAK1-binding protein 3 isoform 1; NFkB activating protein 1. [Source:RefSeq;Acc:NM_152787]"	same	2068	3.44309
X	ENSG00000158311.1	(blank)	opposite	3082	5.95367
X	ENSG00000158813.3	"Ectodysplasin A (Ectodermal dysplasia protein) (EDA protein). [Source:SWISSPROT;Acc:Q92838]"	opposite	2456	12.3885
X				2462	5.76595
X	ENSG00000165509.2	"Melanoma-associated antigen C3 (MAGE-C3 antigen) (Hepatocellular carcinoma-associated antigen 2). [Source:SWISSPROT;Acc:Q8TD91]"	same	1596	5.99934
X			opposite	3485	9.87526
X	ENSG00000165591.2	(blank)	opposite	2340	18.4101
X				2341	5.70138
X	ENSG00000166389.3	(blank)	same	1214	3.88217
X			opposite	3073	2.11847
X	ENSG00000169093.3	"N-acetylserotonin O-methyltransferase-like protein (ASMTL). [Source:SWISSPROT;Acc:O95671]"	opposite	55	3.43196
X	ENSG00000169306.2	"X-linked interleukin-1 receptor accessory protein-like 1 precursor (IL1RAPL-1) (Oligophrenin-4) (Three immunoglobulin domain-containing IL-1 receptor-related 2) (TIGIRR-2). [Source:SWISSPROT;Acc:Q9NZN1]"	opposite	2056	16.4133
X	ENSG00000171478.1	DJ54B20.3 (Novel protein similar to lysozyme C (1	same	414	2.19003
X	ENSG00000173971.2	(blank)	opposite	3432	5.80481

Chr	Ensembl id	Description	Strand	HERV id	HERV score
X	ENSG00000174016.2	(blank)	opposite	2579	7.54324
X	ENSG00000183035.1	"Cylicin I (Multiple-band polypeptide I) (Fragment). [Source:SWISSPROT;Acc:P35663]"	opposite	2673	4.85149
X	ENSG00000184368.3	(blank)	opposite	175	5.73738
X	ENSG00000185010.1	"coagulation factor VIII isoform b precursor; coagulation factor VIIIc; procoagulant component; Factor VIII F8B. [Source:RefSeq;Acc:NM_019863]"	opposite	1747	3.82785
X	ENSG00000186376.3	"Zinc finger protein 75. [Source:SWISSPROT;Acc:P51815]"	opposite	1539	5.59539
X	ENSG00000189108.1	X-linked interleukin-1 receptor accessory protein-like 2 precursor (IL1RAPL-2 related protein) (Interleukin-1 receptor 9) (IL-1R9) (IL-1 receptor accessory protein-like 2) (Three immunoglobulin domain- containing IL-1 receptor-related 1) (TIGIRR-1). [Sour	same	1236	3.8766
X				1237	10.7855
X				1238	13.3859
X			opposite	3104	3.23388
X				3118	8.84552
Y	ENSG00000092377.3	Transducin beta-like 1Y protein (Transducin-beta-like 1	same	3806	5.82141
Y			opposite	4424	17.0534
Y				4426	7.05606
Y	ENSG00000099715.5	"protocadherin 11 Y-linked isoform a; protocadherin Y; protocadherin 22. [Source:RefSeq;Acc:NM_032971]"	opposite	4376	3.04068
Y				4377	12.6966
Y	ENSG00000114374.3	Probable ubiquitin carboxyl-terminal hydrolase FAF-Y (EC 3.1.2.15) (Ubiquitin thiolesterase FAF-Y) (Ubiquitin-specific processing protease FAF-Y) (Deubiquitinating enzyme FAF-Y) (Fat facets protein related	opposite	4533	4.73427
Y	ENSG00000131003.5	RNA binding motif protein	same	4167	1.99414
Y	ENSG00000169953.3	Heat shock transcription factor	opposite	4059	16.187
Y				4060	14.4185
Y	ENSG00000172468.3	Heat shock transcription factor	opposite	4659	13.2314
Y				4660	16.123
Y	ENSG00000180910.4	"Transcript Y 11 protein. [Source:SWISSPROT;Acc:Q9BZ99]"	same	4470	6.33369
Y				4471	9.74092
Y				4472	3.17832
Y				4474	4.91826
Y				4476	3.51396
Y				4477	12.6566
Y				4479	13.6137
Y				4480	20.8664
Y			opposite	3835	23.9626
Y				3836	7.33468
Y				3838	16.7162
Y				3839	29.4245
Y				3843	18.2014
Y				3844	12.7059
Y				3845	8.78368
Y				3846	4.92463
Y				3851	4.97209
Y				3853	4.89907
Y				3854	10.9767
Y	ENSG00000183878.3	"Ubiquitously transcribed Y chromosome tetratricopeptide repeat protein (Ubiquitously transcribed TPR protein on the Y chromosome).	opposite	3906	19.1938

Chr	Ensembl id	Description	Strand	HERV id	HERV score
		[Source:SWISSPROT;Acc:O14607]"			
Y				3908	6.70549
Y				3909	8.3788
Y				3914	18.7425
Y				3915	15.95
