# Supplementary material for: Identification of endogenous retroviral reading frames in the human genome
Source: Retrovirology. 2004 Oct 11;1:32. doi: 10.1186/1742-4690-1-32 (PMC524368; doi:10.1186/1742-4690-1-32)
Supplement: Additional File 2 — Table 2. ESTs matching HERVs containing a long viral ORF. ESTs were compared to HERVs using megaBLAST. Only ESTs that best matched the target HERV were kept. Finally, ESTs mapping conclusively to the same genomic regions as the target HERV were kept. EST library information (organ and tissue) was parsed from Genbank. The positions are in NCBI35 coordinates due to overly stringent settings of EST mappings in the NCBI34 mapping at UCSC. HERV positions were lifted to NCBI35 coordinates using the "lift genome annotations" tool at [file 1742-4690-1-32-S2.pdf]

Additional File 2

Table 2. ESTs matching HERVs containing a long viral ORF.

ESTs were compared to HERVs using megablast. Only ESTs that best matched the target HERV were kept. Finally, ESTs mapping conclusively to the same genomic regions as the target HERV were kept. EST library information (Organ and Tissue) were parsed from Genbank. The positions are in NCBI35 coordinates due to overly stringent settings of EST mappings in the NCBI34 mapping at UCSC. HERV positions were lifted to NCBI35 coordinates using the "lift genome annotations" tool at http://genome.ucsc.edu.

ESTid	HERV id	Organ	Tissue	HERV position	EST position
AI379210	3143	0	B-cell,_chronic_lymphotic_leukemia	chrX:105067535-105070015	chrX:106101476-106101909
AI570707	3143	brain	anaplastic_oligodendroglioma	chrX:105067535-105070015	chrX:106101499-106101924
AI598135	3143	brain	anaplastic_oligodendroglioma	chrX:105067535-105070015	chrX:106101499-106101989
AW812038	5201	stomach	0	chr1:75266332-75270814	chr1:75558716-75559243
BI062752	5201	uterus_tumor	0	chr1:75266332-75270814	chr1:75559344-75559458
BQ304053	5201	breast	0	chr1:75266332-75270814	chr1:75559909-75560340
AI352469	7261	0	pooled_germ_cell_tumors	chr1:152814156-152821460	chr1:152410866-152411356
AI818326	7261	pancreas	adenocarcinoma	chr1:152814156-152821460	chr1:152412558-152413216
AL704166	7261	0	0	chr1:152814156-152821460	chr1:152413728-152414443
AU123401	7261	0	0	chr1:152814156-152821460	chr1:152409880-152410525
AW814393	7261	stomach	0	chr1:152814156-152821460	chr1:152410455-152410939
BE061583	7261	breast	0	chr1:152814156-152821460	chr1:152411168-152411625
BE061602	7261	breast	0	chr1:152814156-152821460	chr1:152411122-152411555
BE061603	7261	breast	0	chr1:152814156-152821460	chr1:152410538-152411053
BE061604	7261	breast	0	chr1:152814156-152821460	chr1:152411111-152411555
BG433950	7261	kidney	0	chr1:152814156-152821460	chr1:152411346-152412156
BG501905	7261	testis	embryonal_carcinoma	chr1:152814156-152821460	chr1:152409770-152420468
BG501920	7261	testis	embryonal_carcinoma	chr1:152814156-152821460	chr1:152410129-152420468
BM910699	7261	brain	astrocytoma_grade_IV,_cell_line	chr1:152814156-152821460	chr1:152410232-152431394
BQ291855	7261	amnion_normal	0	chr1:152814156-152821460	chr1:152410390-152420340

BQ681015	7261	skin	melanotic_melanoma,_cell_line	chr1:152814156-152821460	chr1:152410607-152410977
BX481216	7261	0	0	chr1:152814156-152821460	chr1:152414078-152414677
BX483767	7261	0	0	chr1:152814156-152821460	chr1:152411187-152411803
BX952321	7261	0	0	chr1:152814156-152821460	chr1:152413022-152413638
BX955229	7261	0	0	chr1:152814156-152821460	chr1:152412987-152413575
BX955997	7261	0	0	chr1:152814156-152821460	chr1:152413756-152414438
CB986115	7261	Pooled-Glandular	0	chr1:152814156-152821460	chr1:152410146-152420428
CD104832	7261	Pooled-Skin	0	chr1:152814156-152821460	chr1:152410277-152431408
CD518953	7261	0	White_Matter	chr1:152814156-152821460	chr1:152409941-152431387
T05808	7261	0	0	chr1:152814156-152821460	chr1:152411196-152411539
AI536854	12121	uterus	moderately-differentiated_endometrial_adenocarcinoma,_3	chr3:102732607-102739837	chr3:102893882-102894312
AI669530	12121	prostate	0	chr3:102732607-102739837	chr3:102894229-102894312
AL535594	12121	brain	FETAL_BRAIN	chr3:102732607-102739837	chr3:102898765-102899674
AL698108	12121	0	0	chr3:102732607-102739837	chr3:102896227-102897120
AU119631	12121	0	whole_embryo,_mainly_head	chr3:102732607-102739837	chr3:102895664-102896492
AV727670	12121	0	Hypothalamus	chr3:102732607-102739837	chr3:102900070-102900693
AW614963	12121	lung	carcinoid	chr3:102732607-102739837	chr3:102898605-102898851
BE217830	12121	lung	carcinoid	chr3:102732607-102739837	chr3:102898356-102898851
BF767553	12121	colon_normal	0	chr3:102732607-102739837	chr3:102899853-102900119
BF887722	12121	testis_normal	0	chr3:102732607-102739837	chr3:102899990-102900745
BI004756	12121	head_normal	0	chr3:102732607-102739837	chr3:102898565-102899006
BM310825	12121	Pancreas	Purified_pancreatic_islet	chr3:102732607-102739837	chr3:102895860-102896500
BM310867	12121	Pancreas	Purified_pancreatic_islet	chr3:102732607-102739837	chr3:102896161-102896755
BQ342232	12121	nervous_normal	0	chr3:102732607-102739837	chr3:102898791-102898932
BU853400	12121	testis	0	chr3:102732607-102739837	chr3:102900585-102901319
BX451254	12121	brain	FETAL_BRAIN	chr3:102732607-102739837	chr3:102899493-102899749
BX470668	12121	0	0	chr3:102732607-102739837	chr3:102894726-102895354
BX481275	12121	0	0	chr3:102732607-102739837	chr3:102894696-102895243
BX491192	12121	0	0	chr3:102732607-102739837	chr3:102894803-102895413
CF143144	12121	0	lymph	chr3:102732607-102739837	chr3:102895070-102895724

CF227259	12121	0	embryonic_stem_cells,_cell lines_H1,_H7,_and_H9	chr3:102732607-102739837	chr3:102899498-102900173
CN279095	12121	0	embryonic_stem_cells,_cell lines_H1,_H7,_and_H9	chr3:102732607-102739837	chr3:102895722-102896365
H92683	12121	pineal_gland	0	chr3:102732607-102739837	chr3:102898173-102898519
BU196456	14677	skin	melanotic_melanoma,_cell_line	chr3:186602171-186609460	chr3:186763126-186763288
AI633818	22748	0	pooled_germ_cell_tumors	chr5:156066524-156073823	chr5:156021839-156022427
AI636743	22748	0	pooled_germ_cell_tumors	chr5:156066524-156073823	chr5:156021839-156022452
BQ360868	22748	ovary	0	chr5:156066524-156073823	chr5:156019851-156020177
CF227253	22748	0	embryonic_stem_cells,_cell lines_H1,_H7,_and_H9	chr5:156066524-156073823	chr5:156019794-156020416
CN345079	22748	0	embryonic_stem_cells,_cell lines_H1,_H7,_and_H9	chr5:156066524-156073823	chr5:156024956-156025642
BG004978	24232	placenta_normal	0	chr6:11211667-11219905	chr6:11211552-11212112
BG011064	24232	placenta_normal	0	chr6:11211667-11219905	chr6:11212146-11212501
BQ011017	24232	Placenta	Placenta	chr6:11211667-11219905	chr6:11212683-11213130
BX326241	24232	0	PLACENTA_COT_25-NORMALIZED	chr6:11211667-11219905	chr6:11212303-11213141
BX327880	24232	0	PLACENTA_COT_25-NORMALIZED	chr6:11211667-11219905	chr6:11212604-11213519
BX334611	24232	0	PLACENTA_COT_25-NORMALIZED	chr6:11211667-11219905	chr6:11212957-11213850
BX365962	24232	0	PLACENTA_COT_25-NORMALIZED	chr6:11211667-11219905	chr6:11213221-11213850
BX367795	24232	0	PLACENTA_COT_25-NORMALIZED	chr6:11211667-11219905	chr6:11212383-11213247
BX371758	24232	0	PLACENTA_COT_25-NORMALIZED	chr6:11211667-11219905	chr6:11211474-11212328
BX387071	24232	0	PLACENTA_COT_25-NORMALIZED	chr6:11211667-11219905	chr6:11212122-11213024
BX395188	24232	0	NEUROBLASTOMA_COT_50-NORMALIZED	chr6:11211667-11219905	chr6:11211346-11220034
CB990542	24232	placenta	Human_Placenta	chr6:11211667-11219905	chr6:11213158-11219948
CF529929	24232	Placenta	Placenta	chr6:11211667-11219905	chr6:11212683-11213106
CF995131	24232	placenta	Human_Placenta	chr6:11211667-11219905	chr6:11213357-11219948
T95945	24232	Liver_and_Spleen	0	chr6:11211667-11219905	chr6:11212141-11212320
W33061	24232	parathyroid_gland	parathyroid_tumor	chr6:11211667-11219905	chr6:11211583-11219941
AI970376	26658	0	pooled_germ_cell_tumors	chr7:4367317-4374898	chr7:4400188-4400573
BQ899216	26658	skin	melanotic_melanoma,_cell_line	chr7:4367317-4374898	chr7:4396094-4396938
BU157610	26658	skin	melanotic_melanoma,_cell_line	chr7:4367317-4374898	chr7:4404752-4412455

BU157610	26659	skin	melanotic_melanoma,_cell_line	chr7:4375821-4383401	chr7:4404752-4412455
AA027781	27110	0	placenta	chr7:63862984-63871411	chr7:63895957-63896388
AI032838	27110	Liver_and_Spleen	0	chr7:63862984-63871411	chr7:63896392-63896699
AV702610	27110	0	Adrenal_gland	chr7:63862984-63871411	chr7:63895302-63896048
AV705656	27110	0	Adrenal_gland	chr7:63862984-63871411	chr7:63895418-63896001
AV706536	27110	0	Adrenal_gland	chr7:63862984-63871411	chr7:63895295-63895995
AW194552	27110	kidney	2_pooled_Wilms'_tumors,_one_primary_and_one_metastatic_to	chr7:63862984-63871411	chr7:63896167-63896300
AW955895	27110	0	0	chr7:63862984-63871411	chr7:63895421-63895847
BE222728	27110	brain	oligodendroglioma	chr7:63862984-63871411	chr7:63896157-63896723
BF882678	27110	lung_tumor	0	chr7:63862984-63871411	chr7:63897393-63897869
BG698660	27110	skin	0	chr7:63862984-63871411	chr7:63895007-63895829
BG741949	27110	skin	0	chr7:63862984-63871411	chr7:63895430-63896266
BM354183	27110	Pancreas	Islets_of_Langerhans	chr7:63862984-63871411	chr7:63897543-63911202
BX361372	27110	0	PLACENTA_COT_25-NORMALIZED	chr7:63862984-63871411	chr7:63897411-63911190
BX389809	27110	0	PLACENTA_COT_25-NORMALIZED	chr7:63862984-63871411	chr7:63896664-63897525
CF994104	27110	placenta	Human_Placenta	chr7:63862984-63871411	chr7:63897413-63911224
CF994831	27110	placenta	Human_Placenta	chr7:63862984-63871411	chr7:63897478-63903969
R85001	27110	brain	0	chr7:63862984-63871411	chr7:63895951-63896097
AA426511	27286	mixed_(see_below)	Pooled_human_melanocyte,_fetal_heart,_and_pregnant_uterus	chr7:91710047-91718755	chr7:91742411-91742908
AA776439	27286	Liver_and_Spleen	0	chr7:91710047-91718755	chr7:91743098-91743525
AA781423	27286	0	0	chr7:91710047-91718755	chr7:91742344-91742833
AI128496	27286	placenta	0	chr7:91710047-91718755	chr7:91742345-91742761
AI128526	27286	placenta	0	chr7:91710047-91718755	chr7:91742344-91742758
AU138097	27286	0	placenta	chr7:91710047-91718755	chr7:91743017-91743714
AU138405	27286	0	placenta	chr7:91710047-91718755	chr7:91743065-91743783
BE019603	27286	placenta	choriocarcinoma	chr7:91710047-91718755	chr7:91743058-91743644
BE732673	27286	placenta	choriocarcinoma	chr7:91710047-91718755	chr7:91742345-91742785
BE734284	27286	placenta	choriocarcinoma	chr7:91710047-91718755	chr7:91742906-91743624
BF990707	27286	placenta_normal	0	chr7:91710047-91718755	chr7:91742990-91743340
BF990710	27286	placenta_normal	0	chr7:91710047-91718755	chr7:91743092-91743350
BF990902	27286	placenta_normal	0	chr7:91710047-91718755	chr7:91742990-91743307

BF997514	27286	placenta_normal	0	chr7:91710047-91718755	chr7:91742718-91751486
BG000981	27286	placenta_normal	0	chr7:91710047-91718755	chr7:91744348-91744851
BG004247	27286	placenta_normal	0	chr7:91710047-91718755	chr7:91743051-91743350
BG004254	27286	placenta_normal	0	chr7:91710047-91718755	chr7:91742950-91743342
BG572445	27286	placenta	0	chr7:91710047-91718755	chr7:91742866-91743605
BG984671	27286	colon_normal	0	chr7:91710047-91718755	chr7:91743703-91743797
BI052559	27286	placenta_normal	0	chr7:91710047-91718755	chr7:91743556-91743929
BI052561	27286	placenta_normal	0	chr7:91710047-91718755	chr7:91743556-91743929
BI052566	27286	placenta_normal	0	chr7:91710047-91718755	chr7:91743556-91743924
BI052567	27286	placenta_normal	0	chr7:91710047-91718755	chr7:91743556-91743929
BI052569	27286	placenta_normal	0	chr7:91710047-91718755	chr7:91743556-91743936
BI052570	27286	placenta_normal	0	chr7:91710047-91718755	chr7:91743556-91743929
BI052571	27286	placenta_normal	0	chr7:91710047-91718755	chr7:91743556-91743929
BI052572	27286	placenta_normal	0	chr7:91710047-91718755	chr7:91743556-91743929
BI053219	27286	placenta_normal	0	chr7:91710047-91718755	chr7:91742873-91743159
BI053220	27286	placenta_normal	0	chr7:91710047-91718755	chr7:91742846-91743159
BI053385	27286	placenta_normal	0	chr7:91710047-91718755	chr7:91743556-91743888
BI053386	27286	placenta_normal	0	chr7:91710047-91718755	chr7:91743551-91743884
BI055456	27286	placenta_normal	0	chr7:91710047-91718755	chr7:91743556-91743856
BI055458	27286	placenta_normal	0	chr7:91710047-91718755	chr7:91743551-91743863
BI055459	27286	placenta_normal	0	chr7:91710047-91718755	chr7:91743554-91743859
BI056077	27286	placenta_normal	0	chr7:91710047-91718755	chr7:91743556-91743856
BI056079	27286	placenta_normal	0	chr7:91710047-91718755	chr7:91743551-91743863
BI056080	27286	placenta_normal	0	chr7:91710047-91718755	chr7:91743554-91743859
BI056589	27286	placenta_normal	0	chr7:91710047-91718755	chr7:91743556-91743929
BI056590	27286	placenta_normal	0	chr7:91710047-91718755	chr7:91743654-91743930
BI056592	27286	placenta_normal	0	chr7:91710047-91718755	chr7:91743554-91743898
BI056593	27286	placenta_normal	0	chr7:91710047-91718755	chr7:91743556-91743930
BI056595	27286	placenta_normal	0	chr7:91710047-91718755	chr7:91743554-91743929
BI056596	27286	placenta_normal	0	chr7:91710047-91718755	chr7:91743556-91743871
BI059387	27286	placenta_normal	0	chr7:91710047-91718755	chr7:91743703-91743797
BI087886	27286	cervix	0	chr7:91710047-91718755	chr7:91742344-91743101

BQ022458	27286	Placenta	Placenta	chr7:91710047-91718755	chr7:91744333-91744566
BQ024998	27286	Placenta	Placenta	chr7:91710047-91718755	chr7:91742344-91744593
BQ365139	27286	placenta_normal	0	chr7:91710047-91718755	chr7:91742990-91743338
BQ365143	27286	placenta_normal	0	chr7:91710047-91718755	chr7:91743013-91743342
BQ365206	27286	placenta_normal	0	chr7:91710047-91718755	chr7:91743043-91743342
BQ365207	27286	placenta_normal	0	chr7:91710047-91718755	chr7:91742839-91743319
BQ365241	27286	placenta_normal	0	chr7:91710047-91718755	chr7:91742849-91743078
BQ365242	27286	placenta_normal	0	chr7:91710047-91718755	chr7:91742849-91743077
BQ365279	27286	placenta_normal	0	chr7:91710047-91718755	chr7:91742775-91751468
BQ366047	27286	placenta_normal	0	chr7:91710047-91718755	chr7:91743686-91743791
BQ368374	27286	placenta_normal	0	chr7:91710047-91718755	chr7:91743302-91743411
BX326300	27286	0	PLACENTA_COT_25-NORMALIZED	chr7:91710047-91718755	chr7:91743814-91744556
BX326647	27286	0	PLACENTA_COT_25-NORMALIZED	chr7:91710047-91718755	chr7:91743194-91744069
BX337769	27286	0	PLACENTA_COT_25-NORMALIZED	chr7:91710047-91718755	chr7:91742375-91743368
BX337770	27286	0	PLACENTA_COT_25-NORMALIZED	chr7:91710047-91718755	chr7:91743930-91751648
BX347111	27286	0	PLACENTA_COT_25-NORMALIZED	chr7:91710047-91718755	chr7:91743007-91743851
BX347314	27286	0	PLACENTA_COT_25-NORMALIZED	chr7:91710047-91718755	chr7:91743531-91744459
BX347619	27286	0	PLACENTA_COT_25-NORMALIZED	chr7:91710047-91718755	chr7:91742969-91743836
BX347952	27286	0	PLACENTA_COT_25-NORMALIZED	chr7:91710047-91718755	chr7:91743172-91744121
BX357208	27286	0	PLACENTA_COT_25-NORMALIZED	chr7:91710047-91718755	chr7:91742373-91743133
BX365066	27286	0	PLACENTA_COT_25-NORMALIZED	chr7:91710047-91718755	chr7:91742392-91743403
BX367907	27286	0	PLACENTA_COT_25-NORMALIZED	chr7:91710047-91718755	chr7:91743413-91744238
BX368078	27286	0	PLACENTA_COT_25-NORMALIZED	chr7:91710047-91718755	chr7:91742836-91743879
BX378303	27286	0	PLACENTA_COT_25-NORMALIZED	chr7:91710047-91718755	chr7:91742414-91743394
BX380176	27286	0	PLACENTA_COT_25-NORMALIZED	chr7:91710047-91718755	chr7:91742380-91743347
BX388766	27286	0	PLACENTA_COT_25-NORMALIZED	chr7:91710047-91718755	chr7:91742959-91743783
BX388917	27286	0	PLACENTA_COT_25-NORMALIZED	chr7:91710047-91718755	chr7:91742974-91743440
BX388918	27286	0	PLACENTA_COT_25-NORMALIZED	chr7:91710047-91718755	chr7:91742969-91743372
BX388919	27286	0	PLACENTA_COT_25-NORMALIZED	chr7:91710047-91718755	chr7:91742970-91743238
BX389656	27286	0	PLACENTA_COT_25-NORMALIZED	chr7:91710047-91718755	chr7:91743159-91744081
BX389657	27286	0	PLACENTA_COT_25-NORMALIZED	chr7:91710047-91718755	chr7:91743140-91744081
BX391741	27286	0	PLACENTA_COT_25-NORMALIZED	chr7:91710047-91718755	chr7:91743188-91744122

BX408733	27286	0	PLACENTA	chr7:91710047-91718755	chr7:91743436-91744318
BX408734	27286	0	PLACENTA	chr7:91710047-91718755	chr7:91743513-91744318
BX408752	27286	0	PLACENTA	chr7:91710047-91718755	chr7:91743487-91744395
BX409035	27286	0	PLACENTA	chr7:91710047-91718755	chr7:91743184-91743666
BX409328	27286	0	PLACENTA	chr7:91710047-91718755	chr7:91743486-91744395
BX430050	27286	0	PLACENTA	chr7:91710047-91718755	chr7:91743185-91743806
BX430055	27286	0	PLACENTA	chr7:91710047-91718755	chr7:91743157-91743813
BX439636	27286	0	PLACENTA	chr7:91710047-91718755	chr7:91742425-91743340
CN272394	27286	0	embryonic_stem_cell,_retinoic_acid_and_ mitogen-treated_hES	chr7:91710047-91718755	chr7:91742356-91743113
D78692	27286	0	placenta	chr7:91710047-91718755	chr7:91743860-91744239
H01372	27286	placenta	0	chr7:91710047-91718755	chr7:91742673-91743057
N41878	27286	placenta	0	chr7:91710047-91718755	chr7:91742581-91743000
N53177	27286	Liver_and_Spleen	0	chr7:91710047-91718755	chr7:91742344-91742768
N58614	27286	Liver_and_Spleen	0	chr7:91710047-91718755	chr7:91742787-91743153
N77302	27286	Liver_and_Spleen	0	chr7:91710047-91718755	chr7:91742718-91743149
R27388	27286	placenta	0	chr7:91710047-91718755	chr7:91742729-91743056
R37932	27286	placenta	0	chr7:91710047-91718755	chr7:91743264-91743525
R68740	27286	placenta	0	chr7:91710047-91718755	chr7:91743319-91743734
R76086	27286	placenta	0	chr7:91710047-91718755	chr7:91742361-91742821
R76136	27286	placenta	0	chr7:91710047-91718755	chr7:91742491-91742858
R77278	27286	placenta	0	chr7:91710047-91718755	chr7:91742357-91742846
R78828	27286	placenta	0	chr7:91710047-91718755	chr7:91742641-91743057
T39519	27286	placenta	0	chr7:91710047-91718755	chr7:91743634-91743762
AA584891	27728	0	pheochromocytoma	chr7:152498159-152502575	chr7:152545820-152546208
AA704457	33127	Liver_and_Spleen	0	chr10:101246096-101252303	chr10:101577201-101577664
AW818206	34052	stomach	0	chr11:101104479-101112064	chr11:101077996-101078661
CF227263	34052	0	embryonic_stem_cells,_cell_lines_H1,_H 7,_and_H9	chr11:101104479-101112064	chr11:101075795-101076356
CF227264	34052	0	embryonic_stem_cells,_cell_lines_H1,_H 7,_and_H9	chr11:101104479-101112064	chr11:101075795-101076445
CF227266	34052	0	embryonic_stem_cells,_DMSO- treated_H9_cell_line	chr11:101104479-101112064	chr11:101077858-101078518

CF227268	34052	0	embryonic_stem_cells,_cell_lines_H1,_H7,_and_H9	chr11:101104479-101112064	chr11:101071894-101079622
CF227270	34052	0	embryonic_stem_cells,_cell_lines_H1,_H7,_and_H9	chr11:101104479-101112064	chr11:101071930-101077833
AI480345	34720	kidney	0	chr11:61918339-61924761	chr11:61900592-61900896
AW665466	34720	pooled	0	chr11:61918339-61924761	chr11:61901012-61901603
BE178095	43997	head_neck	0	chr19:20334642-20343232	chr19:20342836-20343355
BE774029	43997	prostate_tumor	0	chr19:20334642-20343232	chr19:20343060-20343469
BQ932595	43997	0	sciatic_nerve	chr19:20334642-20343232	chr19:20341415-20342266
BQ941761	43997	0	sciatic_nerve	chr19:20334642-20343232	chr19:20341254-20342117
CN288807	43997	0	embryonic_stem_cells,_DMSO-treated_H9_cell_line	chr19:20334642-20343232	chr19:20341894-20342616
CB989495	44200	placenta	Human_Placenta	chr19:58202512-58209273	chr19:58202584-58204665
CF593888	44200	placenta	Human_Placenta	chr19:58202512-58209273	chr19:58202584-58204982
CF994266	44200	placenta	Human_Placenta	chr19:58202512-58209273	chr19:58202584-58204696
AI434519	44205	lymph_node	lymphoma,_follicular_mixed_small_and_large_cell	chr19:58245133-58246051	chr19:58245755-58246192
BF999026	44205	placenta_normal	0	chr19:58245133-58246051	chr19:58244814-58244940
CA417098	44205	0	Chondrosarcoma_Cell_line	chr19:58245133-58246051	chr19:58245685-58246008
AA218808	45477	brain	neuroepithelial_cells	chr22:17301017-17308997	chr22:17302724-17303077
AA580921	45477	0	bulk_germ_cell_seminoma	chr22:17301017-17308997	chr22:17308127-17308527
AA922960	45477	0	pooled_germ_cell_tumors	chr22:17301017-17308997	chr22:17302169-17302520
AI632501	45477	0	pooled_germ_cell_tumors	chr22:17301017-17308997	chr22:17301903-17302572
AI635368	45477	0	pooled_germ_cell_tumors	chr22:17301017-17308997	chr22:17301989-17302590
AU124350	45477	0	0	chr22:17301017-17308997	chr22:17308055-17308833
AW206802	45477	0	0	chr22:17301017-17308997	chr22:17304925-17305380
AW812040	45477	stomach	0	chr22:17301017-17308997	chr22:17307469-17307998
AW812041	45477	stomach	0	chr22:17301017-17308997	chr22:17307471-17307998
BE465971	45477	0	pooled_germ_cell_tumors	chr22:17301017-17308997	chr22:17302093-17302590
BE466095	45477	0	pooled_germ_cell_tumors	chr22:17301017-17308997	chr22:17301927-17302590
BE504736	45477	0	pooled_germ_cell_tumors	chr22:17301017-17308997	chr22:17302033-17302566
BE550633	45477	0	pooled_germ_cell_tumors	chr22:17301017-17308997	chr22:17304714-17305584
BF773517	45477	epid_tumor	0	chr22:17301017-17308997	chr22:17306457-17306768

BX955734	45477	0	0	chr22:17301017-17308997	chr22:17301562-17302022
BX956279	45477	0	0	chr22:17301017-17308997	chr22:17301562-17302049
CF227254	45477	0	embryonic_stem_cells,_cell_lines_H1,_H7,_and_H9	chr22:17301017-17308997	chr22:17307036-17307566
CF227255	45477	0	embryonic_stem_cells,_cell_lines_H1,_H7,_and_H9	chr22:17301017-17308997	chr22:17306572-17307218
CF227256	45477	0	embryonic_stem_cells,_DMSO-treated_H9_cell_line	chr22:17301017-17308997	chr22:17306464-17307001
CF227258	45477	0	embryonic_stem_cells,_cell_lines_H1,_H7,_and_H9	chr22:17301017-17308997	chr22:17306465-17307155
CF227260	45477	0	embryonic_stem_cells,_cell_lines_H1,_H7,_and_H9	chr22:17301017-17308997	chr22:17306543-17307068
CF227261	45477	0	embryonic_stem_cells,_cell_lines_H1,_H7,_and_H9	chr22:17301017-17308997	chr22:17305726-17306267
CF227265	45477	0	embryonic_stem_cells,_cell_lines_H1,_H7,_and_H9	chr22:17301017-17308997	chr22:17306679-17307332
CF227267	45477	0	embryonic_stem_cells,_cell_lines_H1,_H7,_and_H9	chr22:17301017-17308997	chr22:17307179-17307846
CF227269	45477	0	embryonic_stem_cells,_cell_lines_H1,_H7,_and_H9	chr22:17301017-17308997	chr22:17307246-17307879
CF227271	45477	0	embryonic_stem_cells,_cell_lines_H1,_H7,_and_H9	chr22:17301017-17308997	chr22:17305577-17306261
CF227272	45477	0	embryonic_stem_cells,_cell_lines_H1,_H7,_and_H9	chr22:17301017-17308997	chr22:17307044-17307768
CF227273	45477	0	embryonic_stem_cells,_cell_lines_H1,_H7,_and_H9	chr22:17301017-17308997	chr22:17306156-17306744
CF227274	45477	0	embryonic_stem_cells,_cell_lines_H1,_H7,_and_H9	chr22:17301017-17308997	chr22:17305571-17306104
CF227275	45477	0	embryonic_stem_cells,_DMSO-treated_H9_cell_line	chr22:17301017-17308997	chr22:17307091-17307391
CN311414	45477	0	embryonic_stem_cells,_cell_lines_H1,_H7,_and_H9	chr22:17301017-17308997	chr22:17307008-17309113
CN311415	45477	0	embryonic_stem_cells,_cell_lines_H1,_H7,_and_H9	chr22:17301017-17308997	chr22:17308700-17309389
CN345078	45477	0	embryonic_stem_cells,_cell_lines_H1,_H7,_and_H9	chr22:17301017-17308997	chr22:17301571-17302221

CN345081	45477	0	embryonic_stem_cells,_cell_lines_H1,_H7,_and_H9	chr22:17301017-17308997	chr22:17301562-17302218
CN345082	45477	0	embryonic_stem_cells,_cell_lines_H1,_H7,_and_H9	chr22:17301017-17308997	chr22:17301533-17302154
CN418483	45477	0	embryonic_stem_cells,_cell_lines_H1,_H7,_and_H9	chr22:17301017-17308997	chr22:17303765-17304400
CN418484	45477	0	embryonic_stem_cells,_cell_lines_H1,_H7,_and_H9	chr22:17301017-17308997	chr22:17303077-17303932
AA196978	45501	0	0	chr22:22205467-22213364	chr22:22205544-22205939
AI623315	45501	0	pooled_germ_cell_tumors	chr22:22205467-22213364	chr22:22205737-22206181
AI917742	45501	0	pooled_germ_cell_tumors	chr22:22205467-22213364	chr22:22205848-22206181
BF858540	45501	prostate_tumor	0	chr22:22205467-22213364	chr22:22206612-22206936
BG980069	45501	colon_normal	0	chr22:22205467-22213364	chr22:22203867-22205937
BG981831	45501	colon_normal	0	chr22:22205467-22213364	chr22:22203767-22205676
BX956126	45501	0	0	chr22:22205467-22213364	chr22:22203631-22205601
CN270291	45501	0	embryonic_stem_cells,_DMSO-treated_H9_cell_line	chr22:22205467-22213364	chr22:22206596-22207078
CN276660	45501	0	embryonic_stem_cells,_DMSO-treated_H9_cell_line	chr22:22205467-22213364	chr22:22207203-22207361
CN279094	45501	0	embryonic_stem_cells,_embryoid_bodies_derived_from_H1,_H7	chr22:22205467-22213364	chr22:22206775-22207390
CN413597	45501	0	embryonic_stem_cells,_cell_lines_H1,_H7,_and_H9	chr22:22205467-22213364	chr22:22203682-22205955
